# Rigid Supramolecular Aramid Nanotubes as Catalyst Supports

**DOI:** 10.1002/adma.202510143

**Published:** 2025-09-22

**Authors:** Yukio Cho, Kiera Y. Tai, Guillaume Lamour, Ty Christoff‐Tempesta, Debaditya Bose Sinha, Yu‐Jin Choi, Rebecca Meacham, Dechen T. Rota, Siyu Wu, Xiaobing Zuo, Julia H. Ortony

**Affiliations:** ^1^ Department of Materials Science and Engineering Massachusetts Institute of Technology Cambridge Massachusetts 02139 USA; ^2^ Université Paris‐Saclay Univ Evry CY Cergy Paris Université CNRS LAMBE Evry‐Courcouronnes 91025 France; ^3^ Department of Chemistry and Biochemistry University of California San Diego La Jolla California 92093 USA; ^4^ X‐ray Science Division Advanced Photon Source Argonne National Laboratory Lemont Illinois 60439 USA; ^5^ Applied Energy Division SLAC National Accelerator Laboratory Menlo Park California 94025 USA; ^6^ Present address: Department of Chemical Engineering Stanford University Stanford California 94305 USA; ^7^ Present address: Department of Materials Science and Engineering University of California Irvine Irvine California 92697 USA; ^8^ Present address: Advanced Functional Polymers Research Center Korea Research Institute of Chemical Technology (KRICT) Yuseong Daejeon 34114 Republic of Korea

**Keywords:** 1D nanomaterials, aramid amphiphiles, heterogeneous catalysis, molecular self‐assembly, nanotubes

## Abstract

Solution‐phase heterogeneous catalysts benefit from nanoscale dimensions, which maximize specific surface area and enhance catalytic activity. However, the ease of recovering such nanocatalysts depends on the design of the support materials, which are often particle‐like. Rigid 1D nanomaterials are proposed as supports that can enhance separability while offering high volumetric specific surface area for greater catalyst loading and activity. Here, aramid amphiphiles (AAs) are designed to spontaneously self‐assemble in water into high‐aspect‐ratio supramolecular nanotubes with tunable surface chemistry. These AA nanotubes exhibit high persistence lengths (*P* = 750 ± 340 µm) and mechanical stiffnesses (3 N/m). Incorporating surface thiol groups enables immobilization of catalytic gold nanoparticles. The resulting AA nanotube‐gold nanoparticle complexes exhibit high catalytic activity, efficient recoverability via simple microfiltration, and sustained reusability over ten reaction cycles. This study demonstrates the utility of molecular self‐assembled 1D nanomaterials as versatile scaffolds for the reuse and recovery of nanoscale catalysts.

## Graphical abstract

Molecular nanotube self‐assemblies with high aspect‐ratios and stiffness are demonstrated as nanocatalyst supports. The extended dimensions and tunable surface chemistries of the nanotubes enable tethered gold nanoparticle catalysts to remain dispersed in solution and to be easily recovered through simple filtration. This study establishes self‐assembled 1D nanomaterials as versatile scaffolds for the reuse and recovery of next‐generation nanocatalysts.

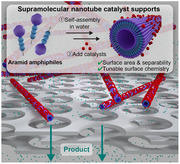

## 1 Introduction

Solution‐phase heterogeneous catalysts and their supports have trended toward progressively smaller dimensions, from the nanoscale to the subnanoscale and even to the single‐atom level. The small dimensions have led to greater efficiency, reduced costs, and superior selectivity.^[^ ^1^, ^2^ ^]^ Recent reports have demonstrated a broad range of nanoscale catalyst designs, including well‐defined metal nanoparticles,^[^ ^3^ ^]^ single‐atom metal complexes,^[^ ^4^ ^]^ enzyme‐like nanomaterials,^[^ ^5^ ^]^ and diverse nanostructures such as nanowires^[^ ^6^ ^]^ and nanofoams.^[^ ^7^ ^]^ However, while size reduction offers advantages such as increased specific surface area and excellent dispersibility, it often comes with the risk of agglomeration and the drawback of reduced recoverability.^[^ ^8^, ^9^ ^]^

Separating and recovering nanoscale catalysts for reuse from solutions that contain reactants and products is a critical step toward circularity and economic feasibility. In general, smaller catalyst supports, particularly those with physical dimensions approaching the sub‐nanometer range, require more energy‐intensive and lower‐throughput separation processes for recovery, such as nanofiltration or ultracentrifugation.^[^ ^10^, ^11^ ^]^ To address this challenge, many designer catalyst supports have been developed, including those with finely tuned mesoporous nano‐ and microstructures,^[^ ^12^ ^]^ open‐framework chemistries to achieve exceptionally high specific surface areas,^[^ ^13^ ^]^ and paramagnetic materials that enable efficient magnetic separation.^[^ ^14^ ^]^ Despite these advances, the morphologies of nanoscale catalyst supports have remained largely limited to conventional particle‐like forms, leaving other architectures relatively underexplored.

1D nanomaterials with high aspect ratios and stiffness offer greater volumetric specific surface area and excellent dispersibility.^[^ ^15^ ^]^ We hypothesize that these structural features can also enhance catalyst separability by allowing for retention through size‐based filtration, compared to conventional particle‐like scaffolds (details and derivation in Note S1, Supporting Information). A variety of 1D nanomaterials have been explored as catalyst supports, leveraging their unique physicochemical properties to improve catalytic performance. For example, carbon nanofibers and nanotubes offer excellent thermal and electrical conductivity, along with mesoporous architectures that provide large, easily accessible surface areas, helping to mitigate mass‐transfer limitations in liquid‐phase reactions.^[^ ^16^, ^17^ ^]^ Similarly, the high aspect‐ratios of electrospun nanofibers lead to enhanced electron confinement effects, which can modulate surface energy and promote catalytic activity.^[^ ^18^, ^19^ ^]^ Nanofibers derived from natural materials, particularly nanocellulose, are not only abundant and biodegradable, but also rich in surface functional groups, allowing for facile modification with metal species to tailor catalytic centers, including in biphasic or multiphase systems.^[^ ^20^, ^21^ ^]^

Despite these advantages, the role of geometry—particularly aspect‐ratio and rigidity—as a design parameter to improve catalyst recovery and retention has received comparatively less attention than surface chemistry of nanocatalysts and their supports.^[^ ^22^, ^23^ ^]^ Moreover, developing 1D nanomaterial platforms that simultaneously combine high aspectratios, mechanical stiffness, solution stability, and tunable surface chemistries remains an outstanding challenge. Herein, we demonstrate the use of molecular self‐assembly to create nanostructures that fulfill these criteria.

Molecular self‐assembly occurs when amphiphilic molecules, possessing both hydrophilic and hydrophobic regions, interact with their aqueous environment to spontaneously form well‐defined nanostructures with precise molecular arrangement.^[^ ^24^ ^]^ By tailoring amphiphile design and self‐assembly conditions, the morphology, dimensions, and dynamics of molecular self‐assemblies can be controlled.^[^ ^25^, ^26^, ^27^ ^]^ Additionally, the surface chemistries of self‐assembled nanostructures can be engineered through the molecular design of the constituent amphiphiles’ hydrophilic domain^[^ ^28^ ^]^. This versatility enables potential applications as catalyst supports, such as immobilizing catalytic metal nanoparticles,^[^ ^29^ ^]^ incorporating catalytic functional groups,^[^ ^30^ ^]^ and facilitating photocatalytic activity.^[^ ^31^ ^]^

Historically, a key challenge with the use of small‐molecule self‐assembly as catalyst supports is their dynamic nature. For example, molecules comprising amphiphilic assemblies can rapidly exchange between nanostructures and solution.^[^ ^32^ ^]^ This instability risks catalytic moieties detaching from the nanostructure surface and diffusing into the solution. To suppress the dynamic character typical to molecular assemblies, we developed a *Kevlar*‐inspired, aramid amphiphile (AA) self‐assembly platform.^[^ ^33^ ^]^ AA nanostructures leverage collective hydrogen bonding and π‐π stacking to surpass molecular exchange, ensuring structural stability. These high‐aspect‐ratio nanostructures exhibit exceptional mechanical robustness, chemical stability, and tunable surface chemistry, making them promising candidates for catalyst support applications.

**Figure 1:**
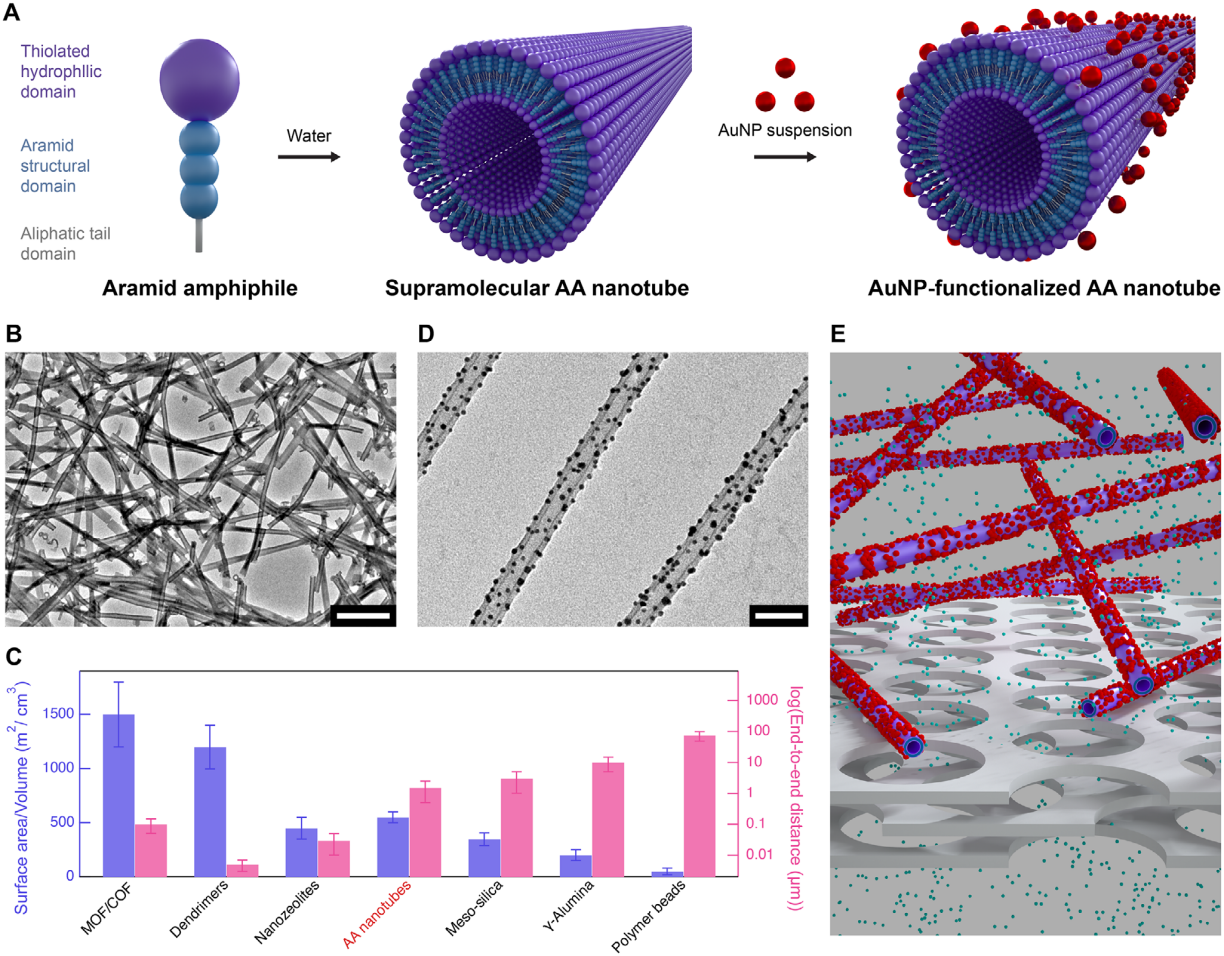
Kevlar‐inspired AAs self‐assemble into nanotubes, demonstrated in this study as catalyst supports for highly active and easily recoverable nanocatalysts in solution‐phase catalysis. a) AAs with a thiolated hydrophilic domain, an aramid structural domain, and an aliphatic tail domain are designed to self‐assemble into supramolecular nanotubes in water. These AA nanotubes subsequently immobilize catalytically active AuNPs on their surfaces through simple suspension mixing. b) Representative TEM image showing a high population of high‐aspect‐ratio supramolecular AA nanotubes (scale bar: 300 nm). c) Comparison of surface‐area‐to‐volume and end‐to‐end distance among commonly used nanocatalyst supports and the AA nanotubes developed in this study.^[^ ^34^, ^35^, ^36^, ^37^, ^38^, ^39^, ^40^ ^]^ The error bars represent the range of values typically reported across literature sources. d) Representative TEM image of AA nanotubes after anchoring AuNPs to their surfaces (scale bar: 50 nm). e) Schematic illustrating how AA nanotube‐supported AuNPs can be easily separated and recovered from reactants and products (shown as small green spheres) through microfiltration, despite their nanoscale dimensions being significantly smaller than the membrane's pore size.

In this work, we design a 1D nanomaterial to demonstrate the concept of high surface‐area, easily separable nanocatalyst supports by leveraging the strong internal cohesion, mechanical robustness, and chemical stabilities of the AA motif. Specifically, we designed AAs that self‐assemble into high aspect‐ratio, rigid nanotubes with thiolated surfaces capable of tethering catalytic gold nanoparticles (AuNPs) on their surface as a model system (**Figure** **1**a). AA nanotubes spontaneously form in water and exhibit micrometer‐scale lengths and nanometer‐scale diameters with a narrow distribution (Figure 1b). Compared to several well‐studied nanocatalyst supports, AA nanotubes uniquely exhibit both high surface‐area‐to‐volume (*SA*/*V*) ratios exceeding 500 m^2^ cm^−3^ and long end‐to‐end distances (*L_E_*) exceeding one micrometer, which enables retention by microfiltration (Figure 1c).^[^ ^34^, ^35^, ^36^, ^37^, ^38^, ^39^, ^40^ ^]^ Simply mixing the suspension of AA nanotubes with AuNPs allows Au─S covalent bonds to form between nanotubes and AuNPs, anchoring the AuNPs onto the surface of the nanotubes (Figure 1d). The resulting AA nanotube‐supported AuNPs catalyze water‐based reactions and can be easily separated and recovered from reactants and products via microfiltration, despite their nanoscale dimensions being significantly smaller than the membrane's pore size (Figure 1e). This study demonstrates the use of 1D nanomaterials derived from molecular self‐assembly as versatile scaffolds that combine high catalytic activity with enhanced separability, offering a promising approach to improving the reuse and recovery of advanced nanocatalysts.

## 2 Results

### 2.1 Self‐Assembled Nanotubes With High Persistence Length and Stiffness

**Figure 2:**
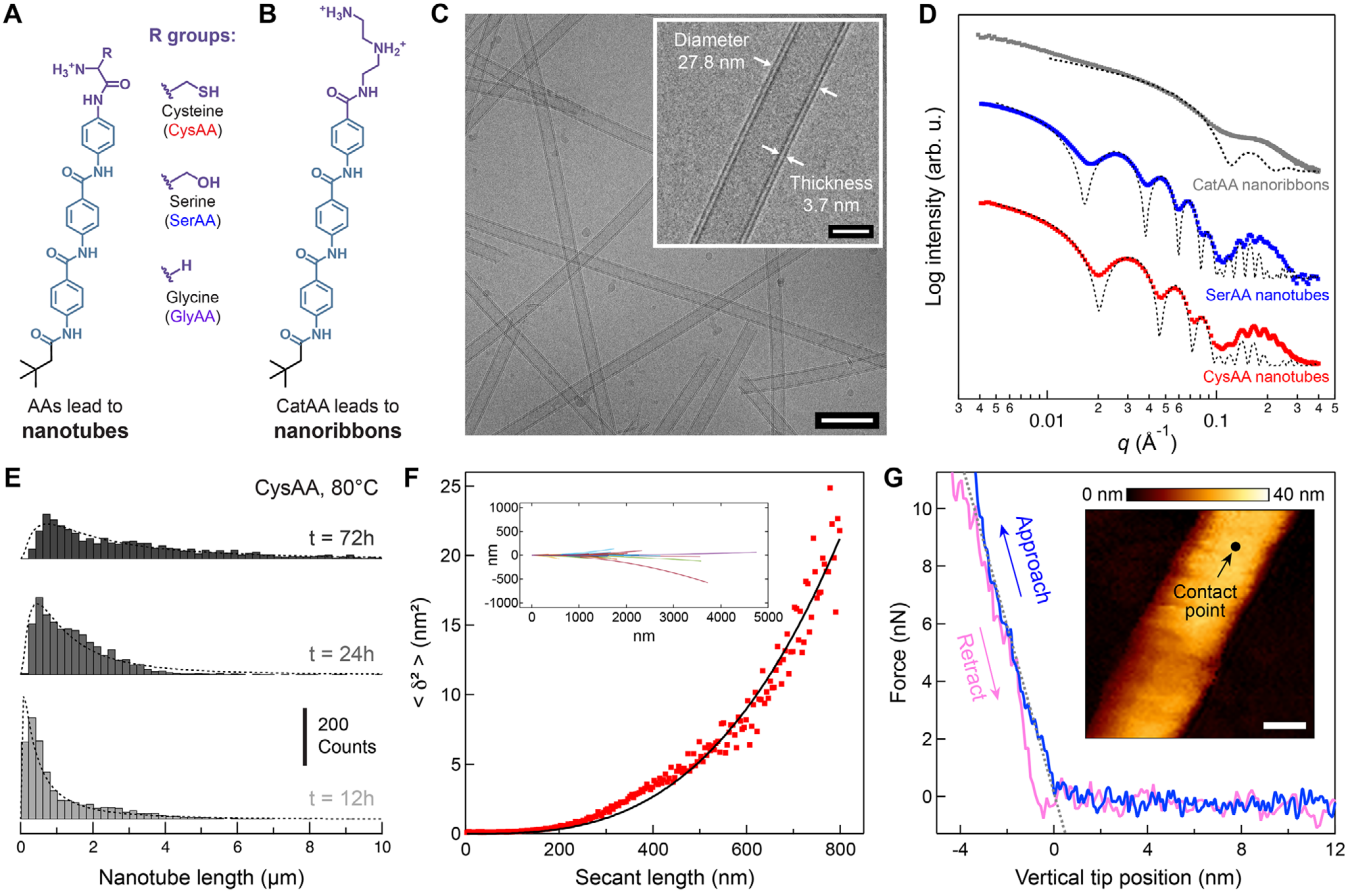
AA nanotubes exhibit high aspect ratios and well‐defined dimensions, characterized by substantial longitudinal persistence length, transverse stiffness, and consistent cross‐section dimensions. a) Chemical structures of AAs with N‐terminal cysteine (CysAA), serine (SerAA), and glycine (GlyAA) head groups, which self‐assemble into nanotubes, and b) a triazaheptane‐based cationic head group (CatAA) as a control sample, which self‐assembles into planar nanoribbons. c) Representative cryo‐TEM image of CysAA nanotubes (scale bar, 100 nm, inset scale bar, 20 nm). d) SAXS profiles of CysAA and SerAA nanotubes and CatAA nanoribbons in suspension. Profiles of nanotubes and nanoribbons are best fit to a hollow cylinder model and a rectangular model, respectively. e) Statistical analysis of the length distribution for CysAA nanotubes with fits to a log‐normal distribution model. f) Least‐squares fitting of midpoint deviations δ of contour traces is used to calculate the persistence length of CysAA nanotubes. <δ^2^> denotes the mean square of δ; inset: contours used for statistical topographical analysis, acquired by tracing the AFM profiles of 60 CysAA nanotubes. g) Representative force‐displacement curve from the QI™ mode of the AFM. (Inset: Force‐displacement measurement is performed at the center of the nanotube, as shown in the representative AFM image. Scale bar, 30 nm.).

We synthesized and purified three AAs with distinct surface chemistries that can self‐assemble to form nanotubes in water (**Figure** **2**a; Figures S3–S22, Supporting Information). All three AA designs feature an aliphatic tail domain with a six‐carbon neopentyl tail group and an aramid structural domain with three aramid repeating units. These features are an analogous to the chemical structure of *Kevlar* (poly(*p*‐phenylene terephthalamide)) and are strategically designed to promote self‐assembly and enhance stability through collective interchain hydrogen bonding and *π‐π* stacking. The three AA designs differ in their head group design, comprising an N‐terminal amino acid that is either cysteine (CysAA), serine (SerAA), or glycine (GlyAA). Compared to a previous AA design with a cationic triazaheptane head group that self‐assembles into a flexible nanoribbon (Figure 2b),^[^ ^33^, ^41^ ^]^ CysAAs, SerAAs, and GlyAAs all form nanotubes upon suspension in water. We hypothesize this difference is attributable to 1) the smaller and less hydrophilic head group domains, and 2) the reversed amide bond between the headgroup and the aramid structural domain (Figure 2a,b). Notably, because the starting chemicals for the head group components in CysAA, SerAA, and GlyAA are widely used in peptide synthesis and are readily available,^[^ ^42^ ^]^ these AAs hold significant potential for scalability and cost efficiency.

Using negative‐stained transmission electron microscopy (TEM), we observe CysAAs, SerAAs, and GlyAAs self‐assemble into high‐aspect‐ratio nanotubes at elevated temperature (Figure S23, Supporting Information). In contrast, the self‐assembly of CatAAs results in the formation of nanoribbons regardless of temperature, consistent with previous reports.^[^ ^33^ ^]^ While a minor focus of this work, we discovered that the self‐assembled morphologies of these AAs are strongly correlated to temperature and pH and show negligible effects from concentration and surface chirality of N‐terminal amino acid head group, as exemplified by CysAA (Figures S24–S32, Supporting Information). Employing high‐resolution cryogenic TEM (cryo‐TEM) further reveals that the CysAA and SerAA nanotubes possess well‐defined dimensions and slightly different diameters. Based on cryo‐TEM images, the exterior diameter of CysAA and SerAA nanotubes is 27.8 and 35.2 nm, respectively, with similar nanotube wall thicknesses of 3.7 and 3.5 nm (Figure 2c; Figure S33, Supporting Information). Although the dimensions obtained from conventional TEM images are less accurate than those from cryo‐TEM, the exterior diameter of GlyAA nanotubes appears larger(≈50 nm), and they also exhibit greater flexibility (Figure S23, Supporting Information). These observations suggest that the packing motifs of CysAA and SerAA nanotubes likely resemble each other, resulting in similar wall thicknesses. However, the differing non‐covalent interactions at the surface of the nanotubes likely lead to variations in the preferred curvature of the nanotubes, and consequently, different diameters and flexibility.

The contrast in geometry and packing motifs between nanotubes and nanoribbons is also evident in X‐ray scattering patterns. Small angle X‐ray scattering (SAXS) profiles of CysAA and SerAA nanotubes, which best fit a hollow cylinder, differ significantly from the profile obtained for CatAA nanoribbons, which best fit a lamellar bilayer model (Figure 2d).^[^ ^43^, ^44^ ^]^ Through fitting, we obtain exterior diameters of 29.4 and 34.7 nm and wall thicknesses of 5.7 and 5.9 nm for CysAA and SerAA nanotubes, respectively (Figure S34, Supporting Information). These dimensions are consistent with the results from cryo‐TEM images (Figure 2c; Figure S33, Supporting Information). The SAXS profile of GlyAA nanotubes shows features similar to a hollow cylinder model but could not be accurately fit, likely due to the substantial flexibility of the nanotubes, which complicates the form factor for SAXS analysis (Figure S35, Supporting Information). Wide angle X‐ray scattering (WAXS) profiles of CysAA and SerAA nanotubes exhibit two distinct peaks at *d* = 5.1 and *d* = 5.0 Å. In contrast, CatAA nanoribbons show only a single peak at *d* = 5.0 Å, indicative of their hydrogen bonding network (Figure S36, Supporting Information). This result suggests that the molecular arrangement in rigid AA nanotubes involves two types of hydrogen bonding interactions, distinct from those in flexible AA nanoribbons. Given the structural similarity among the synthesized AAs in this study, we selected CysAA as a representative model to further characterize nanotube growth behavior and mechanical properties.

Interestingly, we observed that the exterior diameter and wall thickness of nanotubes remain constant when the suspension is held at 80 °C, but their length exhibits a clear time dependence. We performed a statistical analysis of CysAA nanotubes using TEM images, taken after various equilibration times (Figure 2e; Figure S37, Supporting Information). We observed that the length distributions of CysAA nanotubes follow a log‐normal function, which is a common probability distribution for grain sizes in crystalline materials.^[^ ^45^ ^]^ Extending the equilibration time from 12 to 24, and then to 72 h increases all central measures of CysAA nanotube length (Table S3, Supporting Information); for instance, the median length rises from 0.54 to 1.27 and 2.43 µm, respectively. This log‐normal distribution of nanotube lengths suggests that nanotube formation proceeds through random nucleation, followed by growth at the nanotube edges—analogous to the random nucleation and growth mechanism observed in crystalline materials.^[^ ^46^ ^]^ This hypothesis would explainwhy longer tubes grow more slowly, because there is less possibility of AAs appearing near the edge in longer tubes. Additionally, elongation of the AA nanotubes was observed by attenuated total reflectance Fourier‐transform infrared spectroscopy (ATR‐FTIR). The FT‐IR spectra show two peaks at 1653 and 1632 cm^−1^ that emerge upon annealing at 80°C, and the peaks become more pronounced as the annealing time increases (Figure S38, Supporting Information). These two peaks are attributed to two distinct modes of carbonyl (C═O) amide I stretching, indicating the evolution of two types of hydrogen bonding, as observed in previous WAXS profiles (Figure S36, Supporting Information).

A unique feature of self‐assembled AA nanostructures is their remarkable mechanical properties due to the strong intermolecular cohesion.^[^ ^33^ ^]^ For 1D nanostructures, such as nanofibers, nanowires, and nanotubes, persistence length (*P*) is a typical mechanical parameter used to quantify bending stiffness along the longitudinal direction.^[^ ^47^, ^48^, ^49^ ^]^ We characterized the persistence length of CysAA nanotubes using a statistical topographical analysis of atomic force microscope (AFM) images. After equilibrating the nanotubes in water on a glass surface, parametric splines to the contours were traced from 60 CysAA nanotubes (Figure S39, Supporting Information). A persistence length of *P* = 750 ± 340 µm was determined through least‐squares fitting to the midpoint deviations δ of contour traces (Figure 2f).^[^ ^50^ ^]^ Although persistence length is not an intrinsic parameter and depends on cross‐section dimensions, its magnitude is two orders higher than that of most reported 1D nanomaterials composed of small molecules, such as amyloid fibrils and organic nanotubes.^[^ ^51^, ^52^ ^]^ The high persistence length of CysAA nanotubes evidences their remarkable rigidity, approaching the level of inorganic materials such as carbon nanotubes.^[^ ^53^ ^]^ Additionally, we evaluated stiffness along the transverse direction of CysAA by capturing force‐displacement curves at the center of the nanotube using AFM. We observed that CysAA nanotubes remained intact under up to 10 nN of transverse loading and ≈10% of transverse strain (Figure 2g). Furthermore, the overlap between approach and retraction traces of the force‐displacement curves confirms the hysteresis of this measurement is low, indicating that the nanotubes restore most of their elastic energy upon release of the load. The linear slopes of the approach curves allow for the calculation of stiffness as 3 N m^−1^ for CysAA nanotubes (Figure S40, Supporting Information), and nanotube stiffness remains unchanged even after two years of storage in deionized water at room temperature (Figure S41, Supporting Information). We also confirmed that a 24 h treatment of the nanotubes in 2 m NaCl, 1 m NaOH, or absolute ethanol did not substantially affect either the nanotube stiffness or morphology (Figure S42, Supporting Information). In addition, exposure to acidic, oxidizing, and reducing conditions did not affect the nanotube structures (Figure S44, details in Note S12, Supporting Information). AA nanotube stiffness is significantly greater than that of biomimetic tubes, which typically exhibit stiffness on the order of 0.01 N m^−1^, underscoring the rigidity of the AA nanotubes.^[^ ^54^ ^]^ Notably, this value approaches carbon nanotube moduli – one of the stiffest materials known – that are typically on the order of 10 to 100 N/m.^[^ ^55^ ^]^

### 2.2 Nanotube‐Supported AuNPs With High Catalytic Performance and Separability

**Figure 3:**
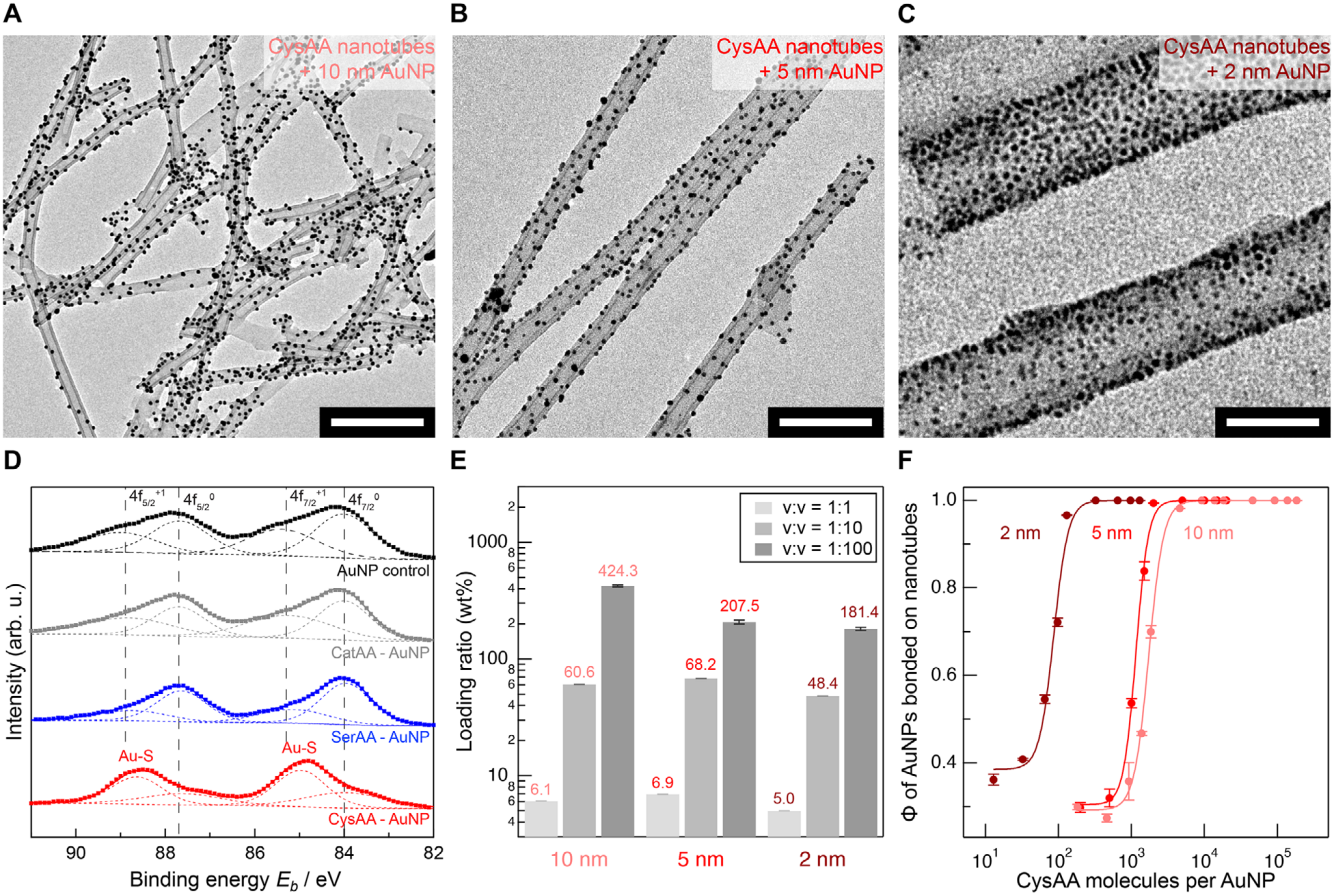
Thiolated CysAA nanotube surfaces enable uniform immobilization of AuNPs of various diameters. Representative conventional TEM images are shown: a) CysAA nanotubes with 10 nm diameter AuNPs, b) CysAA nanotubes with 5 nm diameter AuNPs, and c) CysAA nanotubes with 2 nm diameter AuNPs. Scale bars correspond to 200, 100, and 30 nm, respectively. d) Au4f XPS spectra of CysAA‐supported AuNPs, SerAA and CatAA complexed with AuNPs, and AuNPs control group. All powder samples were lyophilized from aqueous suspensions. e) Loading ratios of three AuNP sizes on CysAA nanotubes as a function of the volumetric mixing ratio of CysAA suspension (1 mg/ml) to AuNP solution in its as‐received concentration. f) Binding isotherms of AuNPs of varying sizes as a function of the molar ratio of CysAA molecules per AuNP.

After characterizing the morphologies and rigidity of the AA nanostructures, we examined their ability to support AuNPs. CysAA nanotubes were mixed with AuNPs of mean diameters 10, 5, and 2 nm, and in each case, the nanoparticles were evenly distributed and selectively anchored on the nanotube surfaces (**Figure** **3**a–c). In contrast, AuNPs mixed with the SerAA and CatAA nanostructures without thiolated surface chemistries tend to aggregate (Figure S45, Supporting Information). Although the surface chemistries of amine and hydroxyl functional groups in these AA nanostructures are also expected to interact electrostatically with AuNPs, these observations underscore that the thiol functional group is crucial for supporting AuNPs on AA nanostructures.^[^ ^56^ ^]^ Analysis of TEM images suggests that AuNPs primarily anchor on the outer surfaces of CysAA nanotubes, although some AuNPs may reach the tube interiors, which we expect are geometrically more difficult to access (Figure S46, Supporting Information).

To further verify the Au─S covalent bonding in CysAA nanotube‐supported AuNPs, X‐ray photoelectron spectroscopy (XPS) was carried out.^[^ ^57^ ^]^ The Au 4f XPS spectrum of lyophilized CysAA nanotube‐supported AuNPs displays distinct Au chemical states compared to the control of AuNPs dried directly, with aggregated AuNPs (Figure 3d). The majority of Au 4f peaks for CysAA nanotube‐supported AuNPs were attributed to Au─S covalent bonding.^[^ ^58^ ^]^ In contrast, AuNPs mixed with control AA nanostructures display a spectrum identical to the AuNP control, corroborating the aggregation observed by TEM.^[^ ^59^ ^]^ The binding energies of the Au 4f peaks have been corrected using the carbon 1s XPS as the internal standard (Figure S47, Supporting Information).

The high surface density of anchoring sites on CysAA nanotubes is attributed to the thiol functional group present on every constituent CysAA, which is exposed on the nanostructure surface. To evaluate the extent of catalytic site exposure, we conducted a quantitative analysis of AuNP loading on CysAA nanotubes. By adjusting the mixing ratio between nanotubes and AuNPs in the suspension, the loading of AuNPs onto the nanotubes can be easily tuned, ranging from a few wt.% to several hundred wt.% (Figure 3e; Figure S48, Supporting Information). Under AuNP‐saturated conditions, the loading ratios for 10, 5, and 2 nm AuNPs were 424, 208, and 181 wt.%, respectively. For comparison, the theoretical mass ratio per particle for these sizes is ≈125:16:1, indicating disproportionately higher loadings for smaller AuNPs. This trend suggests that smaller nanoparticles can attach more densely on the nanotube surface, as corroborated by the TEM images, and may also have a greater likelihood of reaching geometrically difficult‐to‐access interior sites. Unlike approaches that directly grow sub‐nanometer metal nanoparticles on supports, our strategy decouples AuNP synthesis from their immobilization, enabling the tethering of AuNPs of arbitrary size to the nanotube surface with tunable coverage.^[^ ^60^ ^]^

Isotherm data collected after mixing CysAA nanotube suspensions with 10, 5, and 2 nm AuNPs reveal the minimum molar ratios of CysAA required to fully immobilize AuNPs from solution onto the nanotube surface for reusable catalysis (Figure 3f). These ratios are ≈5,000, 3,000, and 250 mol CysAA: mol AuNP for 10, 5, and 2 nm AuNPs, respectively. These values correspond to mass ratios of 0.4, 2, and 5 g CysAA: g AuNP for 10, 5, and 2 nm AuNPs, respectively. Fitting the CysAA‐AuNP binding isotherm with the Hill equation yields apparent dissociation constants of 10–100 µm, indicating a moderately strong gold‐thiol affinity comparable to values reported for peptide–AuNP conjugates (Table S5, Supporting Information).^[^ ^61^ ^]^ The Hill coefficient of ≈4 to 5 suggests cooperative binding, where the attachment of one thiol group to an AuNP facilitates the binding of neighboring thiol groups to the same particle, likely due to a sterically favorable arrangement on the nanotube surface.

**Figure 4:**
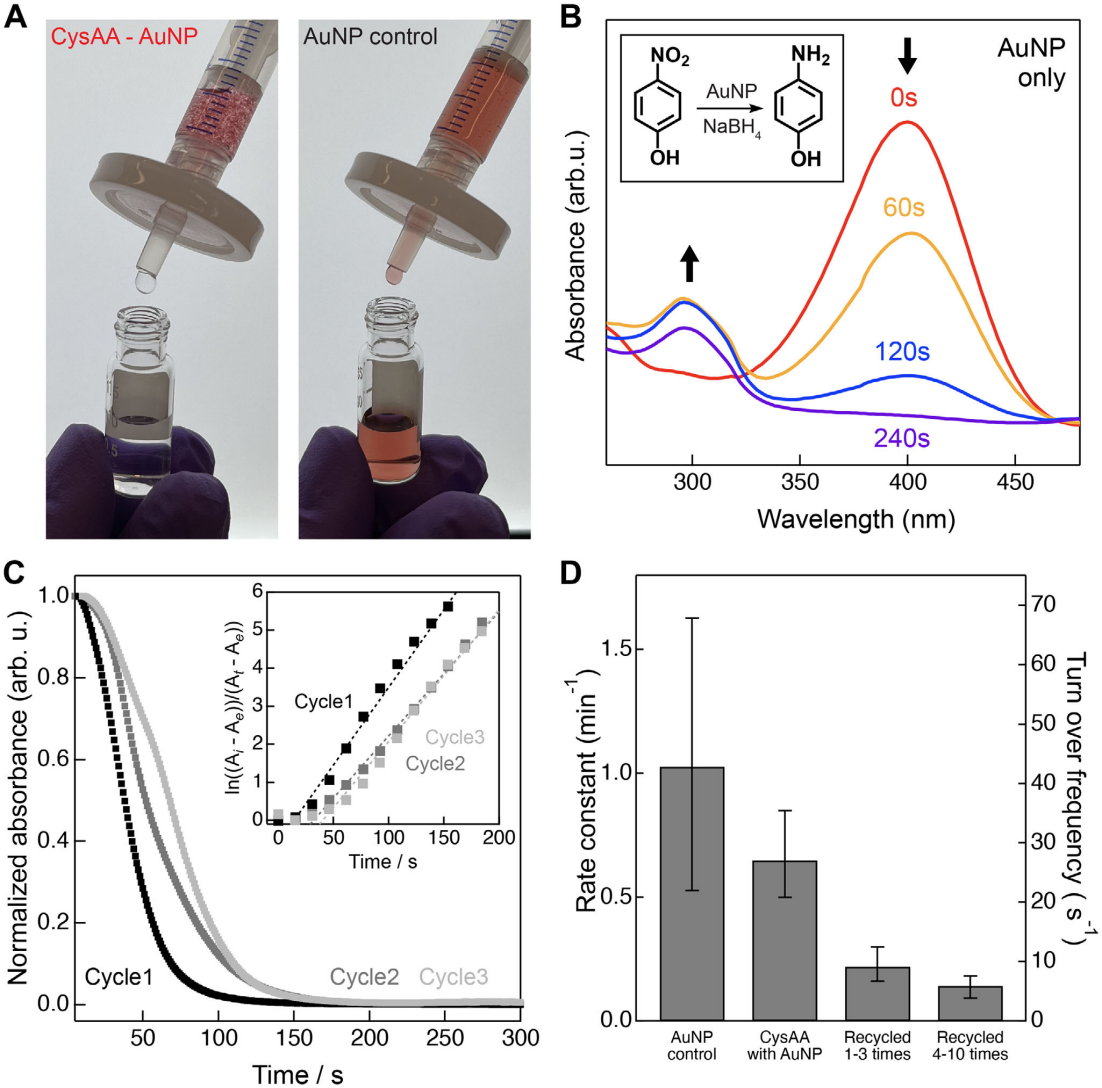
CysAA nanotube‐supported AuNPs are highly active and reusable nanocatalysts. a) AuNPs supported by CysAA nanotubes can be easily separated from solution via simple microfiltration, in contrast to the control containing only AuNPs, which passes through the filter membrane due to the significant size difference between the nanoparticles and the pores. b) UV–vis absorbance spectra over time during the reduction of 4‐NP using AuNPs as catalysts in the presence of NaBH_4_. c) Normalized absorbance at 400 nm during the reduction of 4‐NP for the first three cycles of recovering 10 nm AuNPs supported by CysAA nanotubes from the previous reaction. (Inset: First‐order plots normalized from the time‐dependent absorbance. Here, A_i_ represents the initial absorbance intensity, and A_e_ denotes the lowest absorbance value, assumed to be the endpoint of the reaction.) d) Rate constant and calculated turnover frequency of the CysAA nanotube‐supported 10 nm AuNPs over multiple recovery cycles.

Next, we demonstrate the retention of AA nanotube‐supported AuNPs through microfiltration. While AuNPs with sub‐10 nm diameters cannot be separated from solution by common filtration methods, supporting AuNPs onto rigid and high‐aspect‐ratio CysAA nanotubes enables their retention by simple separation using readily available filtration systems. We examined the separation of CysAA nanotube‐AuNP systems from aqueous solutions using syringe filters with a 0.2 µm pore size (**Figure** **4**a). Once the solution has passed through the filter, the nanotubes can be easily recovered into a new suspension by backwashing the filter with water. We confirmed that the filtrate was colorless, and the UV‐visible (UV–vis) spectrum of the filtrate did not show any absorption from AuNPs (Figure S49, Supporting Information). We also carried out inductively coupled plasma‐mass spectroscopy (ICP‐MS) analysis to quantitatively determine the amount of AuNPs that passed through the filter. Beside the initial filtrate, which exhibited ≈0.5 ppm of gold likely attributed to the short nanotubes, we found that the filtrate contained an undetectable level of gold even after recovering the nanotubes ten times (Figure S50, Supporting Information). The ability to both distribute and separate AA nanotube‐supported AuNPs in and from water ‐ maximizing the kinetics of the catalytic reaction and enabling separation by facile filtration ‐indicates their potential as quasi‐homogeneous catalysts.

Finally, we demonstrated the catalytic performance and reusability of CysAA nanotube‐supported AuNPs. We chose the reduction reaction of 4‐nitrophenol (4‐NP) to 4‐aminophenol (4‐AP) with sodium borohydride (NaBH_4_) as a model reaction because its kinetics can be quantified by monitoring the absorption intensity of 400 nm light using UV–vis spectroscopy (Figure 4b).^[^ ^62^, ^63^ ^]^ The time dependence of absorbance at 400 nm remained nearly consistent over the first three reaction cycles, during which the CysAA supported 10 nm AuNPswere separated from the reacted solution by filtration, recovered in fresh deionized water, and reused in subsequent reactions (Figure 4c). By normalizing the time dependence of absorbance, a first‐order plot was obtained, from which a rate constant can be extracted. The rate constant for CysAA‐supported AuNPs was approximately half that of an AuNP control. This is a reasonable outcome given the expected decrease in the surface‐to‐volume ratio of the AuNPs following immobilization. While a gradual decline in catalytic activity is observed over repeated use, the CysAA nanotube‐supported AuNPs retain the majority of their catalytic performance after ten reaction cycles, indicating potential of reusability and operational stability (Figure 4d). After these cycles, we also verified that the nanostructures of the CysAA nanotube‐supported AuNPs remained intact (Figure S51, Supporting Information). The turnover frequency (TOF), calculated based on the known particle concentration of AuNPs, yields 21.5 s^−1^ for the first cycle and 6.0 s^−1^ after 10 cycles, conservatively assuming the reaction completeness to be 80%. These values are comparable to TOFs reported for catalysts used in industrial applications, which typically range from 10^−2^‐10^2^ s^−1^.^[^ ^64^ ^]^ The observed reduction in reaction rate over cycles may be optimized in future studies. It is likely due to a partial side reaction between NaBH_4_ and CysAA nanotubes, consistent with the detection of ≈1 to 2 ppm in the filtrate by ICP‐MS (Figure S52, Supporting Information). Additionally, a small number of short CysAA nanotube‐supported AuNPs were able to pass through the filter in later cycles (Figure S53, Supporting Information). We also tested the conventional method of centrifugation to separate the AuNP‐supported nanotubes from the solution. While centrifugation effectively removed the AuNP component from the solution, it caused significant agglomeration of the nanotube–AuNP complexes, leading to poor redispersibility in fresh solvent during subsequent cycles (Figure S54, Supporting Information). In total, these results demonstrate that stiff 1D nanotubes serve as a promising scaffold for balancing reusability with catalytic activity of nanoscale catalysts.

## 3 Conclusion

In summary, we propose that harnessing the rigidity and high aspect ratio of 1D nanomaterials as supports can enhance the separability of nanocatalysts without compromising their nanoscale advantages. In this work, we leveraged molecular self‐assembly to construct surface‐tunable and robust nanotubular catalyst supports. Our aramid amphiphile platform selectively self‐assembles into high‐aspect‐ratio nanotubes, enabling three distinct surface chemistries, CysAA, SerAA, and GlyAA, while the aramid motif provides chemical stability and mechanical robustness. We confirmed that CysAA nanotubes exhibit well‐defined dimensions in water, with a substantial persistence length of P = 750 ± 340 um and a high transverse stiffness of ∽3 N/m. The thiolated surface of CysAA nanotubes facilitates the formation of Au–S covalent bonds, enabling uniform immobilization of AuNPs across the nanotube surface, where the loading ratio can be tuned from a few wt.% to several hundred wt.%. In contrast, AA nanosturctures without thiolated surface chemistries, such as SerAA nanotubes and CatAA nanoribbons, rely on weaker electrostatic interactions, which proved  insufficient to stably support AuNPs without aggregation. Importantly, AA nanotube‐supported AuNPs could be separated from the solution by a simple microfiltration process. Nanotube‐supported AuNPs demonstrated high catalytic activity for the reduction of 4‐NP and maintained performance across ten cycles of recovery and reuse. Estimated TOFs of 21.5 s^−1^ for the first cycle and 6.0 s^−1^ after 10 cycles are comparable to values reported for industrial catalysts. The versatility of this approach suggests its promise for broader potential applications of self‐assembled 1D nanomaterials as scaffolds for molecular catalysts, enzymes, and co‐assembled catalysts. Altogether, this work lays the groundwork for leveraging the unique geometric features of 1D nanomaterials to create nanocatalyst supports with enhanced separability, paving the way for scalable, high‐performance, and reusable nanocatalysts through molecular self‐assembly.

## 4 Experimental Section

### General Procedure

The synthesis details of various AAs, including (*S*)‐*N*‐(4‐(2‐amino‐3‐mercaptopropanamido)phenyl)‐4‐(4‐(3,3‐dimethylbutanamido)benzamido)benzamide (**D‐CysAA**), (*R*)‐*N*‐(4‐(2‐amino‐3‐mercaptopropanamido)phenyl)‐4‐(4‐(3,3‐dimethylbutanamido)benzamido)benzamide (**L‐CysAA**), (*R*)‐*N*‐(4‐(2‐amino‐3‐hydroxypropanamido)phenyl)‐4‐(4‐(3,3‐dimethylbutanamido)benzamido)benzamide (**D‐SerAA**), and (*S*)‐*N*‐(4‐(2‐amino‐3‐hydroxypropanamido)phenyl)‐4‐(4‐(3,3‐dimethylbutanamido)benzamido)benzamide (**L‐SerAA**) are detailed in the (Note S2, Supporting Information). The self‐assembly of AA nanotubes was achieved by suspending AAs in DI water and treating the solutions at specified temperatures for predetermined time periods. Lyophilized AA nanostructures were obtained by using a LABCONCO FreeZone benchtop freeze dryer. Synthesis details of CatAA control and nanoribbon preparation are described elsewhere.^[^ ^41^ ^]^AA nanotube‐supported AuNPs were prepared by vortexing AA nanostructure suspensions with AuNPs, followed by shaking on a platform shaker for 5 min by to ensure homogeneity. The concentrations of AA nanostructures were maintained at 1 mg mL^−1^, and AuNPs were used as received. A 1:10 volumetric ratio (AA nanosturctures:AuNP suspension) was used throughout the study unless noted. The vendor‐reported concentration of AuNPs used in this study was 6.0 × 10^12^, 5.5 × 10^13^, and 8.5 × 10^14^ particles mL^−1^ for 10, 5, and 2 nm AuNPs, respectively.

### Chemical Characterization

Proton (^1^H) and carbon (^13^C) nuclear magnetic resonance (NMR) spectra were captured on a Bruker Avance III DPX 400. ≈20 mg of each sample was dissolved in 500 µL of deuterated dimethylsulfoxide (DMSO‐*d*_6_) for analysis, and chemical shifts were measured in parts per million (ppm) downfield. UV‐visible absorption spectra, including time‐dependent UV–vis measurements, were collected using a PerkinElmer LAMBDA 850+ UV/Vis spectrophotometer. These measurements were conducted in rectangular quartz cells with a path length of 10.0 mm. The concentration of all AA suspensions for UV–vis absorption spectra was consistently set at 0.05 mm. Circular dichroism (CD) spectra were recorded on a Jasco J‐1500 CD spectrometer after diluting the AA suspension to 0.1 mm. ATR‐FTIR spectra of CysAA nanotubes over various annealing times were acquired using a Bruker ALPHA II ATR‐FTIR spectroscope, with the AA concentration fixed at 10 mg mL^−1^ in deuterated water (D_2_O). D_2_O was specifically chosen over water to minimize interference in the infrared region of interest. X‐ray photoelectron spectroscopy (XPS) spectra were recorded on a PHI VersaProbe II X‐ray Photoelectron Spectrometer using a mono‐energetic Al Kα X‐rays source. CasaXPS software was employed to fit the XPS core level spectra with peak calibration to the C 1s peak. The gold concentrations in filtrates were analyzed using an Agilent 7900 inductively coupled plasma‐mass spectrometer (ICP‐MS). Prior to ICP‐MS measurement, each sample was diluted by 50–90% with an aqueous solution containing 2 % hydrochloric acid and 2 % nitric acid. Concentration calibration curves were constructed using the original AuNP suspension with a known concentration obtained from Sigma‐Aldrich.

### TEM and Cryo‐TEM

Transmission electron microscopy (TEM) images were obtained using an FEI Tecnai G2 Spirit TWIN microscope, operating at an accelerating voltage of 120 kV. TEM sample grids were prepared by depositing a 1 mg mL^−1^ AA suspension onto a carbon grid (Electron Microscopy Sciences, 200 mesh, copper) for 30 s. Excess solution was blotted off, followed by the application of a 1% phosphotungstic acid solution (Electron Microscopy Sciences) onto the grid, with subsequent blotting to remove excess stain. Cryogenic‐TEM (cryo‐TEM) images of AA nanotubes were captured using an FEI Tecnai Arctica microscope at an accelerating voltage of 200 kV. Cryo‐TEM grids were prepared using an FEI Vitrobot Mark IV. Holey carbon grids (Electron Microscopy Sciences, Quantifoil R1.2/1.3 300 mesh, copper) were glow‐discharged, and then 3.0 µ of a 0.5 mg mL^−1^ AA nanotube suspension was applied onto these grids in a chamber maintained at 95% humidity. Following a blotting time of 6 s, the grids were rapidly plunged into liquid ethane (C_2_H_6_), then transferred to liquid nitrogen (N_2_) and subsequently loaded into the microscope for imaging. The defocus during image collection was set at ‐5.0 µm.

### Transmission X‐ray Scattering

Small‐angle (SAXS) and wide‐angle X‐ray scattering (WAXS) measurements of AA suspensions were conducted at Beamline 12‐ID‐B of the Advanced Photon Source at Argonne National Laboratory. The experiments utilized an X‐ray radiation energy of 13.3 keV. A DECTRIS PILATUS 300 K detector and a PILATUS 2M detector were used for SAXS and WAXS measurements, respectively. Following self‐assembly, AA suspensions were transferred into 2‐mm‐diameter quartz capillary tubes (Hampton Research). Background signals from water and capillary tubes were subtracted to isolate X‐ray scattering specifically attributable to AA nanostructures. The fitting of SAXS data was carried out using SasView software and is described in greater detail in Note S6 (Supporting Information).

### AFM Imaging and Mechanical Characterization

Atomic force microscopy (AFM) imaging was conducted using a Bruker/JPK Nanowizard 4 atomic force microscope, equipped with BL‐AC40‐TS cantilevers (Olympus, nominal spring constant of 0.1 N m^−1^). The AFM images were captured in fast‐QI™ mode,^[^ ^65^ ^]^ at a resolution of 512 pixels × 512 pixels, using a scanning speed corresponding to a vertical tip velocity of 220 µm s^‒1^. The fluctuations observed in the nanotube shapes from AFM images were statistically processed by the Easyworm software.^[^ ^44^ ^]^ For direct indentation of the nanotubes, the approach and retract curves were collected using QI™ mode with OTESPA cantilevers (Olympus, nominal spring constant of 33 N m^−1^). The cantilevers were driven vertically at a speed of 12 µm s^−1^, with the maximum force applied on the nanotube set at 10 nN before retraction. The sensitivity and spring constant of the cantilevers were calibrated by thermal tuning. More comprehensive details about sample preparation and experimental procedures are available in Note S11 (Supporting Information).

### Catalysis Characterization and Recovery

AA nanotube‐supported AuNPs, prepared per the general procedure, were added to rectangular quartz cells with a path length of 10.0 mm. The suspensions were then diluted with DI water to a fixed volume of 2.1 mL, followed by the addition of 50 µL of 1 mmol 4‐NP to the quartz cell. After incorporating 50 µL of freshly prepared 50 mmol NaBH_4_, the absorbance of the UV–vis spectrum at 400 nm was recorded over time. The reaction was considered complete once the 400 nm absorbance peak reached a steady value. The separation and recovery of the AA nanotube‐supported AuNPs were conducted by transferring the mixture into a 5 mL syringe and filtering the liquid through a syringe filter (25 mm diameter, VWR, PTFE, 0.22 µm pore size). The AA nanotube‐supported AuNPs, identifiable by their red color, were observed to be retained on the filter during the separation process. Subsequently, the syringe filter was backwashed by drawing 2.1 mL of deionized (DI) water through it while attached to the syringe, resulting in the red‐colored nanotubes being redispersed in the DI water within the syringe. The recovered AA nanotube‐supported AuNPs, now dispersed in 2.1 mL of DI water, were then reintroduced to the quartz cell for reuse in the next catalytic cycle.

## Conflict of Interest

The authors declare that a patent (US National Phase Application No. 18/992,892) has been filed based on the work described in this manuscript. The authors have no other competing interests to declare.

## Author Contributions

Y.C. conceived of and designed the experiments; Y.C., Y‐J.C., K.T., and D.T.R. synthesized materials and prepared samples; Y.C. and Y‐J.C. performed NMR; Y.C., K.T., D.T.R., and R.M. performed UV‐vis; R.M. performed circular dichroism; Y.C. performed conventional TEM, cryo‐TEM, ATR‐FTIR, and XPS; Y.C. and K.T. performed statistical analysis of length distribution; S.W. and X.Z. Performed synchrotron X‐ray scattering; G.L. performed AFM imaging, statistical topographical analyses, AFM‐based mechanical test, and analysis of data; Y.C. and K.T. performed catalysis characterization; T. C.‐T. and D.B.S performed ICP‐MS; Y.C. wrote and completed formal analysis and presentation of the data in the manuscript; J.H.O., T. C.‐T. and Y.C. edited the manuscript; J.H.O. provided project administration, funding acquisition, and supervision.

## Supporting information



Supporting Information

## Data Availability

The data that support the findings of this study are available from the corresponding author upon reasonable request.
