# Supplementary material for: Rigid Supramolecular Aramid Nanotubes as Catalyst Supports
Source: Adv Mater. 2025 Sep 22;38(6):e10143. doi: 10.1002/adma.202510143 (PMC12848653; doi:10.1002/adma.202510143)
Supplement: Supplementary file 1 — Supporting Information [file ADMA-38-e10143-s001.docx]

Supplementary Information

Rigid Supramolecular Aramid Nanotubes as Catalyst Supports

Yukio Cho^1†#*^, Kiera Y. Tai^1^, Guillaume Lamour^2^, Ty Christoff-Tempesta^1‡^, Debaditya Bose Sinha^3^, Yu-Jin Choi^1^, Rebecca Meacham^1^, Dechen T. Rota^4^, Siyu Wu^5^, Xiaobing Zuo^5^, Julia H. Ortony^1,3*^

^1^ Department of Materials Science and Engineering, Massachusetts Institute of Technology, Cambridge, Massachusetts 02139, United States

^2^ LAMBE, Université Paris-Saclay, University of Evry, CNRS, Evry-Courcouronnes, France

^3^ Department of Chemistry and Biochemistry, University of California San Diego, La Jolla, California 92093, United States

^4^ Department of Chemical Engineering, Massachusetts Institute of Technology, Cambridge, Massachusetts 02139, United States

^5^ X-ray Science Division, Advanced Photon Source, Argonne National Laboratory, Lemont, Illinois 60439, United States

^†^ Current Address: Department of Chemical Engineering, Stanford University, Stanford, California 94305, United States

^#^ Current Address: Applied Energy Division, SLAC National Accelerator Laboratory, Menlo Park, California 94025, United States

^‡^ Current Address: Department of Materials Science and Engineering, University of California Irvine, Irvine, California 92697, United States

*Correspondence to: [yukiocho@stanford.edu](mailto:yukiocho@stanford.edu), j[ortony@ucsd.edu](mailto:ortony@ucsd.edu)

**This PDF file includes:**

**Supplementary Note 1:** Analytical formulation of nanostructures’ volumetric specific surface area and end-to-end distance ································································································································ **Pg. 02**

**Supplementary Note 2:** Materials and synthesis ············································································ **Pg. 07**

**Supplementary Note 3:** TEM of AA nanostructures ····································································· **Pg. 22**

**Supplementary Note 4:** Influence of temperature on CysAA self-assembly ···························· **Pg. 23**

**Supplementary Note 5:** Influence of pH on CysAA self-assembly ············································ **Pg. 24**

**Supplementary Note 6:** Influence of concentration on CysAA self-assembly ························· **Pg. 25**

**Supplementary Note 7:** Influence of surface chirality on CysAA self-assembly ····················· **Pg. 27**

**Supplementary Note 8:** cryo-TEM image of SerAA nanotubes ·················································· **Pg. 28**

**Supplementary Note 9:** Small- and wide- angle X-ray scattering ··············································· **Pg. 29**

**Supplementary Note 10:** Time dependence of CysAA self-assembly at 80°C ························· **Pg. 31**

**Supplementary Note 11:** AFM images and mechanical analysis ················································ **Pg. 33**

**Supplementary Note 12:** CysAA nanotube stability under various conditions ························ **Pg. 35 Supplementary Note 13:** AuNP aggregation on non-thiolated AA nanostructures ·················· **Pg. 37**

**Supplementary Note 14:** XPS correction ························································································ **Pg. 37**

**Supplementary Note 15:** Tunable AuNP loading on CysAA naotubes ······································ **Pg. 38**

**Supplementary Note 16:** Retention of AuNP-functionalized AA nanotubes ···························· **Pg. 40**

**Supplementary Note 1:** Analytical formulation of nanostructures’ volumetric specific surface area and end-to-end distance

**Overview**

We propose that one-dimensional (1D) nanomaterials with high aspect-ratios and stiffness offer greater volumetric specific surface area, and we hypothesize that their separability is enhanced compared to conventional particle-like scaffolds.^[1]^ For spherical nanoparticles, the surface area ($SA$) normalized by volume ($V$) is inversely proportional to their average radius (Supplementary Table 1). In contrast, for 1D nanomaterials such as nanofibers and nanotubes, the $SA/V$ approaches a constant when their length ($L$) is significantly greater than their radius, with this constant determined by the outer ($R$) and inner ($r$) radii. Thus, regardless of their length, nanofibers and nanotubes maintain a high volumetric specific surface area.^[2]^

**Supplementary Table 1 |** Volumetric specific surface area ($SA/V$) for spherical nanoparticles, semi-flexible nanofibers, and rigid nanotubes, expressed as a function of their volume and diameters.

| Spherical nanoparticles | Semi-flexible nanofibers | Rigid nanotubes |
| --- | --- | --- |
| $\frac{SA}{V}\propto\frac{1}{R}$ | $\frac{SA}{V}\propto\frac{1}{L}+\frac{1}{R}$ | $\frac{SA}{V}\propto\frac{1}{L}+\frac{1}{R-r}$ |

Another geometric feature that favors rigid nanotubes for enhanced separability is their substantially longer end-to-end distance ($L_{E}$), compared to other geometries with the same nanostructure volume. $L_{E}$ refers to the maximum possible distance between two points on the nanostructure.^[3]^ Maximizing $L_{E}$ can simplifying the retention of catalysts and their supports post-reaction by increasing the membrane pore size required to separate these nanostructures from a solution by filtration. For spherical nanoparticles, $L_{E}$ is simply the average diameter of the particle, which scales with $\sqrt[3]{V}$ and is inversely proportional to $SA/V$ (Supplementary Table 2). In contrast, the $L_{E}$ of 1D nanomaterials is significantly higher than spherical architectures. For semi-flexible nanofibers, in which $L$ is much greater than the persistence length ($P$), $L_{E}$ can be estimated using the worm-like chain model for semi-flexible polymers, which scales with $\sqrt[2]{V}$.^[4,5]^ For rigid nanotubes, the $L_{E}$ is even longer and is expected to have a linear relationship with the $V$ of the nanotube. Although the surface chemistry of nanocatalysts and their supports undeniably plays a significant role in surface reactivity and catalytic activity,^[6,7]^ the unique geometric features of rigid nanotubes, compared to flexible nanofibers and spherical particles, present an opportunity to simultaneously maximize size-based separability (as a function of $L_{E}$) and catalytic activity (as a function of $SA/V$).

**Supplementary Table 2 |** End-to-end distance ($L_{E}$) for spherical nanoparticles, semi-flexible nanofibers, and rigid nanotubes, expressed as a function of their volume.

| Spherical nanoparticles | Semi-flexible nanofibers^*^ | Rigid nanotubes |
| --- | --- | --- |
| $L_{E}\propto\sqrt[3]{V}$ | $L_{E}\propto\sqrt[2]{V}$ | $L_{E}\propto V$ |

* When the nanofiber length $L$ is much greater than the persistence length ($P$).

**Volumetric specific surface area**

For nanoparticles assuming a spherical geometry, the surface area ($SA$) and volume ($V$) can be expressed in terms of the average radius ($R$) as follows:

$$SA=4\pi R^{2}, V=\frac{4}{3}\pi R^{3}$$

The volumetric specific surface area ($SA/V$) in terms of radius is given by:

$$\frac{SA}{V}=\frac{3}{R}$$

Rewriting this expression in terms of volume:

$$\frac{SA}{V}=\sqrt[3]{\frac{36\pi}{V}}$$

This shows that the volumetric specific surface area for nanoparticles is inversely proportional to the cube root of volume:

$$\frac{SA}{V}\propto\frac{1}{\sqrt[3]{V}}$$

For semi-flexible nanofibers, the surface area ($SA$) and volume ($V$) can be expressed in terms of the average radius ($R$) of nanofiber’s cross-section and its length ($L$) as follows:

$$SA=2\pi R^{2}+2\pi LR, V=\pi{LR}^{2}$$

The volumetric specific surface area ($SA/V$) in terms of radius and length is:

$$\frac{SA}{V}=\frac{2}{L}+\frac{2}{R}$$

For nanofibers with a high aspect-ratio ($L\gg R)$, this expression simplifies to:

$$\lim_{L\gg R} \frac{SA}{V}=\frac{2}{R}$$

This result indicates that for high-aspect-ratio semi-flexible nanofibers, the volumetric specific surface area becomes independent of length and is solely determined by the cross-section radius:

$$\lim_{L\gg R} \frac{SA}{V}=Const$$

Similarly, for rigid nanotubes, the surface area ($SA$) and volume ($V$) can be expressed in terms of the nanotube’s outer radius ($R$), inner radius ($r$), and its length ($L$) as follows:

$$SA=2\pi\left( R^{2}-r^{2} \right)+2\pi L\left( R+r \right), V=\pi L\left( R^{2}-r^{2} \right)$$

The volumetric specific surface area ($SA/V$) in terms of radii and length is:

$$\frac{SA}{V}=\frac{2}{L}+\frac{2}{R-r}$$

For nanotubes with a high aspect-ratio ($L\gg R, L\gg r)$, this expression simplifies to:

$$\lim_{L\gg R} \frac{SA}{V}=\frac{2}{R-r}$$

This result indicates that for high-aspect-ratio rigid nanotubes, the volumetric specific surface area becomes independent of length and is solely determined by the cross-section dimensions:

$$\lim_{L\gg R} \frac{SA}{V}=Const$$

Supplementary Figure 1 presents the numerical simulation of the volumetric specific surface area as a function of nanostructure volume for three different types of nanostructures.

**Supplementary Figure 1 |** The volumetric specific surface area for rigid nanotubes, semi-flexible nanofibers, and spherical nanoparticles as functions of the nanostructure’s volume. In the numerical simulations presented here, the nanotube is modeled with a fixed 30 nm diameter and 5 nm wall thickness, and the nanofiber is simulated with a fixed 20 nm diameter.

**End-to-end distance**

Here, we define the end-to-end distance ($L_{E}$) as the maximum possible distance between two points on a nanostructure. For spherical nanoparticles, $L_{E}$ corresponds to the diameter of the particle and can be expressed as:

$$L_{E}=2R$$

Rewriting this expression in terms of volume:

$$L_{E}=\sqrt[3]{\frac{6}{\pi}V}$$

This shows that the end-to-end distance for nanoparticles is proportional to the cube root of volume:

$$L_{E}\propto\sqrt[3]{V}$$

For semi-flexible nanofibers, we use the worm-like chain model to describe end-to-end distance $(L_{E})$.^[4,8]^ The mean square end-to-end distance is given by:

$$\left\langle{L_{E}}^{2} \right\rangle=2PL\left[ 1-\frac{P}{L}\left( 1-e^{-L/P} \right) \right]$$

Where $P$ is the persistence length, and $L$ is the total length of the nanofiber. This leads to expression of:

$$L_{E}=\sqrt{2PL-2P^{2}\left( 1-e^{-L/P} \right)}$$

For high aspect-ratio and semi-flexible nanofibers, where the total length is much greater than the persistence length ($L\gg P)$, the exponential term approaches zero:

$$\lim_{L\gg P} e^{-L/P}=0$$

Thus, this expression simplifies to:

$$\lim_{L\gg P} L_{E}=\sqrt{2PL-2P^{2}}$$

Rewriting this expression in terms of volume:

$$\lim_{L\gg P} L_{E}=\sqrt{\frac{2P}{\pi R^{2}}V-2P^{2}}$$

This result indicates that for high-aspect-ratio semi-flexible nanofibers, when the persistence length is much smaller than total length ($L\gg P)$, and both $P$ and $R$ remain constant (i.e., the mechanical properties do not change), the end-to-end distance is proportional to the square root of volume:

$$\lim_{L\gg P} L_{E}\propto\sqrt{V}$$

For rigid nanotubes, we now consider the case where the persistence length is much greater than the total nanotube length ($P\gg L)$, the exponential term in the expression of $L_{E}$ can be approximated using a Taylor series expansion:

$$\lim_{P\gg L} e^{-L/P}\approx1-\frac{L}{P}+\frac{L^{2}}{2P^{2}}$$

Substituting this approximation back into the expression of $L_{E}$:

$$\lim_{P\gg L} L_{E}=\sqrt{2PL-2P^{2}\left( 1-1+\frac{L}{P}-\frac{L^{2}}{2P^{2}} \right)}$$

This expression simplifies to:

$$\lim_{P\gg L} L_{E}=\sqrt{2PL-2PL+L^{2}}$$

Which further reduces to:

$$\lim_{P\gg L} L_{E}=L$$

This result indicates that for high-aspect-ratio rigid nanotubes, when the persistence length is much greater than the total length ($P\gg L)$, and $P$, $R$, and $r$ remain constant (i.e., the mechanical properties do not change), the end-to-end distance is directly proportional to the volume:

$$L_{E}\propto V$$

Supplementary Figure 2 presents the numerical simulation of the end-to-end distance as a function of nanostructure volume for three different types of nanostructures.

**Supplementary Figure 2 |** The end-to-end distance for rigid nanotubes, semi-flexible nanofibers, and spherical nanoparticles as functions of the nanostructure’s volume. In the numerical simulations presented here, the nanotube is modeled with a fixed 30 nm diameter and 5 nm wall thickness, and the nanofiber is simulated with a worm-like chain model with a consistent 20 nm diameter and a persistence length of 500 nm.

**Supplementary Note 2:** Materials and synthesis

**Materials and overview**

Methyl 4-aminobenzoate (Sigma Aldrich, 98%), 3,3-dimethylbutyric acid (Sigma Aldrich, 98%), *N*-Boc-*p*-phenylenediamine (Sigma Aldrich, 97%), Boc-D-Cys(Trt)-OH (Ambeed, 97%), Boc-S-trityl-L-cysteine (Chem Impex, 99.5%), Boc-D-Ser(tBu)-OH (Ambeed, 95%), Fmoc-*O*-*tert*-butyl-L-serine (Chem Impex, 99.5%), Boc-Gly-OH (Sigma Aldrich, 99%), 1,4-bis-Boc-1,4,7-triazaheptane (BBT, Chem Impex, 100%), 1-ethyl-3-(3-dimethylaminopropyl)carbodiimide hydrochloride (EDC, TCI Chemicals, 98%), 4-dimethylaminopyridine (DMAP, TCI Chemicals, 99%), 1-hydroxybenzotriazole hydrate (HOBt, TCI Chemicals, 97%), *N*,*N*- Diisopropylethylamine (DIPEA, Alfa Aesar, 99%), triisopropylsilane (TIPS, Oakwood Chemicals, 98%), lithium hydroxide monohydrate (LiOH.H_2_O, Alfa Aesar, 98%), sodium bicarbonate (NaHCO_3_, Alfa Aesar, 99%), hydrochloric acid (HCl, Alfa Aesar, 36%), acetonitrile (Fisher Chemical, HPLC grade), methanol (Fisher Chemical, HPLC grade), dichloromethane (Fisher Chemical, HPLC grade), tetrahydrofuran (Fisher Chemical, HPLC grade), dimethylformamide (Fisher Chemical, ACS grade), diethyl ether (Fisher Chemical, ACS grade), trifluoroacetic acid (TFA, Alfa Aesar, 99%), and piperidine (Thermo Scientific Chemicals, 99%) were used as received without further purification. The gold nanoparticle suspension used in this study was obtained from Sigma-Aldrich for the 5 and 10 nm diameters (OD 1, stabilized suspension in 0.1 mM PBS, reactant-free), and the 2 nm diameter was obtained from Nanopartz Inc. (1.8 nm diameter, OD 1, without capping agent, suspended in DI water). 4-Nitrophenol (Sigma Aldrich, 99%), Sodium borohydride (NaBH_4_, Sigma Aldrich, 99%) and 25 mm diameter syringe filters (VWR, PTFE, 0.22 µm pore size) were used as received, without further purification, in the demonstration of the catalytic activity and separation by filtration.

This study relied on carbodiimide-mediated coupling reactions, deprotection of *tert*-butyloxycarbonyl (Boc) groups, fluorenylmethoxycarbonyl (Fmoc) groups, triphenylmethyl (trityl) groups, *tert*-butyl groups, and ester hydrolysis to synthesize free N-terminal cysteine aramid amphiphile (CysAA) and free N-terminal serine aramid amphiphile (SerAA) with D- and L-enantiomers of the amino acid head group, and free N-terminal glycine aramid amphiphile (GlyAA). The chemical composition of intermediates and products were confirmed through ^1^H and ^13^C nuclear magnetic resonance (NMR) and liquid chromatography-mass spectroscopy (LC-MS). The synthesis and NMR spectra of the control compound (CatAA) have been reported previously.^[9,10]^ The synthesis scheme, details of CysAA, SerAA and their intermediates, and the NMR spectra of the compounds newly synthesized in this study are given in Supplementary Figure 8 to 22.

**Synthesis of D-CysAA and L-CysAA**

******

**Supplementary Figure 3 |** Synthesis scheme to obtain the aramid amphiphile with D-chirality cysteine derivative head group (D-CysAA).

Methyl 4-(3,3-dimethylbutanamido)benzoate (**1)**: A solution of methyl 4-aminobenzoate (33 mmol), 3,3-dimethylbutyric acid (50 mmol), EDC (100 mmol), and DMAP (100 mmol) in dimethylformamide (150 mL) was stirred at 60°C for 24 h. After the reaction, DI water (2 L) was added to the solution to precipitate the crude product, which was then collected by filtration. The crude product was dissolved in methanol (30 mL) and precipitated by adding the solution to a 5wt% NaHCO_3_ aqueous solution (50 mL). The white precipitate that formed was collected by filtration, washed with a 5wt% NaHCO_3_ aqueous solution, and dried under vacuum to yield the product. (yield: 87%). ^1^H NMR (400 MHz, DMSO-*d*_6_): δ = 7.89 (d, 2H), 7.75 (d, 2H), 3.82 (s, 3H), 2.23 (s, 2H), 1.03 (s, 9H) ppm.

4-(3,3-dimethylbutanamido)benzoic acid (**2)**: LiOH·H_2_O (289 mmol) in deionized water (60 mL) was mixed with compound **1** (28.9 mmol) in tetrahydrofuran (240 mL) and methanol (120 mL). The solution was then refluxed for 24 h. After the reflux, the volatile components were removed under vacuum, and the mixture was then neutralized with a 5% aqueous HCl solution (300 mL). The white precipitate was obtained by filtration, washed with 5% aqueous HCl and deionized water, and dried under vacuum to yield the product (yield: 99%). ^1^H NMR (400 MHz, DMSO-*d*_6_): δ = 7.87 (d, 2H), 7.72 (d, 2H), 2.23 (s, 2H), 1.03 (s, 9H) ppm.

Methyl 4-(4-(3,3-dimethylbutanamido)benzamido)benzoate (**3**): A solution of methyl 4-aminobenzoate (45 mmol), compound **2** (30 mmol), EDC (90 mmol), and DMAP (90 mmol) in dimethylformamide (200 mL) was stirred at 60°C for 24h. After the reaction, DI water (2 L) was added to the solution to precipitate the crude product, which was collected by filtration. The crude product was then further washed with an excess of methanol and acetonitrile and dried under vacuum to obtain the product (yield: 69%). ^1^H NMR (400 MHz, DMSO-*d*_6_): δ = 7.95 (m, 6H), 7.77 (d, 2H), 3.84 (s, 3H), 2.24 (s, 2H), 1.04 (s, 9H) ppm. The impurity with δ = 3.17 (d), 2.89 (s), 2.74 (s) is carried to and removed in the next step.

4-(4-(3,3-dimethylbutanamido)benzamido)benzoic acid (**4**): LiOH·H_2_O (200 mmol) in DI water (80 mL) was mixed with compound **3** (20 mmol) in tetrahydrofuran (320 mL) and methanol (160 mL). The solution was then refluxed for 24 h. Afterwards, the volatile components were removed under vacuum, and the mixture was neutralized with a 5% aqueous HCl solution (300 mL). The pink crude product was obtained by filtration, dried sufficiently, and then added to methanol (500 mL). The resulting white precipitate was further obtained by filtration, washed with an excess of methanol and acetonitrile, and dried under vacuum to yield the product (yield: 99%). ^1^H NMR (400 MHz, DMSO-*d*_6_): δ = 7.93 (m, 6H), 7.76 (d, 2H), 2.24 (s, 2H), 1.04 (s, 9H) ppm.

*tert*-Butyl 4-(4-(4-(3,3-dimethylbutanamido)benzamido)benzamido)phenylcarbamate (**5**): A solution of *N*-Boc-*p*-phenylenediamine (36 mmol), compound **4** (18 mmol), EDC (54 mmol), and DMAP (54 mmol) in dimethylformamide (200 mL) was stirred at 60°C for 24 h. After the reaction, DI water (2 L) was added to the solution to precipitate the crude product, which was collected by filtration. The crude product was then washed with excess of methanol and dried under vacuum to obtain the product (yield: 90%).^1^H NMR (400 MHz, DMSO-*d*_6_): δ = 7.96 (m, 6H), 7.77 (d, 2H), 7.64 (d, 2H), 7.41 (d, 2H), 2.25 (s, 2H), 1.46 (s, 9H), 1.05 (s, 9H) ppm.

*N*-(4-(amino)phenyl)-4-(4-(3,3-dimethylbutanamido)benzamido)benzamide (**6**): TFA (20 mL) was slowly added to the solution of compound 5 (15 mmol) in dichloromethane (180 mL). After stirring the mixture overnight at room temperature, the volatiles were distilled off. The remaining mixture was washed with DI water and a 5wt% NaHCO_3_ aqueous solution. The solid precipitate was filtered and dried under vacuum (yield: 99%). ^1^H NMR (400 MHz, DMSO-*d*_6_): δ = 7.95 (m, 6H), 7.75 (d, 2H), 7.48 (d, 2H), 6.72 (d, 2H), 2.25 (s, 2H), 1.05 (s, 9H) ppm.

*tert*-butyl(*S*)-(1-((4-(4-(4-(3,3-dimethylbutanamido)benzamido)benzamido)phenyl)amino)-1-oxo-3-(tritylthio)propan-2-yl)carbamate (**7**): Compound **6** (0.71 mmol), Boc-D-Cys(Trt)-OH (2.16 mmol), EDC (2.16 mmol), HOBt (2.16 mmol), and DIPEA (2.16 mmol) were mixed in dimethylformamide (15 mL) and dichloromethane (15 mL) at room temperature for 24 h. After the reaction, the solvent was removed under vacuum, and DI water (500 mL) was added to precipitate the crude product, which was collected by filtration. The orange crude product was further dissolved into acetonitrile (400 mL), and the insoluble impurities were removed by vacuum filtration. The solvent in the filtrate, which contained the product, was removed under vacuum, and dichloromethane (400 mL) was added to precipitate the product. The solid precipitate was filtered and dried under vacuum (yield: 56%). ^1^H NMR (400 MHz, DMSO-*d*_6_): δ = 7.96 (m, 6H), 7.74 (q, 4H), 7.55 (d, 2H), 7.29 (m, 15H), 7.06 (d, 1H), 4.21 (m, 1H), 6.72 (d, 2H), 2.25 (s, 2H), 1.39 (s, 9H), 1.05 (s, 9H) ppm.

(*S*)-*N*-(4-(2-amino-3-mercaptopropanamido)phenyl)-4-(4-(3,3-dimethylbutanamido)benzamido)benzamide (**D-CysAA**): Compound **7** (0.4 mmol) and TIPS (1.2 mmol) were mixed in TFA (25 mL) and dichloromethane (25 mL). The solution was then refluxed at room temperature for 24 h. Afterwards, the volatile components were removed under vacuum, and the product was precipitated by adding diethyl ether (100 mL). The solid precipitate was filtered, washed with diethyl ether excessively, and dried under vacuum to yield the product (yield: 99%). ^1^H NMR (400 MHz, DMSO-*d*_6_): δ = 7.96 (m, 6H), 7.77 (d, 4H), 7.59 (d, 2H), 4.03 (t, 1H), 3.01 (m, 2H), 2.25 (s, 2H), 1.05 (s, 9H) ppm. ^13^C NMR (400 MHz, DMSO-*d*_6_): δ = 171.0, 166.7, 165.6, 165.2, 142.9, 142.8, 136.0, 134.1, 129.8, 129.2, 129.1, 128.9, 121.3, 120.3, 119.8, 118.8, 55.7, 50.1, 47.4, 44.6, 35.8, 31.3, 30.1, 26.2 ppm. MS (LC/MS) [M + H]^+^ m/z calculated 548.2; [M + H]^+^ found 547.2.

******

**Supplementary Figure 4 |** Synthesis scheme to obtain the aramid amphiphile with L-chirality cysteine derivative head group (L-CysAA).

*tert*-butyl(*R*)-(1-((4-(4-(4-(3,3-dimethylbutanamido)benzamido)benzamido)phenyl)amino)-1-oxo-3-(tritylthio)propan-2-yl)carbamate (**8**): Compound **6** (0.71 mmol), Boc-S-trityl-L-cysteine (2.16 mmol), EDC (2.16 mmol), HOBt (2.16 mmol), and DIPEA (2.16 mmol) were mixed in dimethylformamide (15 mL) and dichloromethane (15 mL) at room temperature for 24 h. After the reaction, the solvent was removed under vacuum, and DI water (500 mL) was added to precipitate the crude product, which was collected by filtration. The orange crude product was further dissolved into acetonitrile (400 mL), and the insoluble impurities were removed by vacuum filtration. The solvent in the filtrate, which contained the product, was removed under vacuum, and dichloromethane (400 mL) was added to precipitate the product. The solid precipitate was filtered and dried under vacuum (yield: 36%). ^1^H NMR (400 MHz, DMSO-*d*_6_): δ = 7.95 (m, 6H), 7.74 (q, 4H), 7.55 (d, 2H), 7.29 (m, 15H), 7.06 (d, 1H), 4.21 (m, 1H), 6.72 (d, 2H), 2.25 (s, 2H), 1.39 (s, 9H), 1.05 (s, 9H) ppm.

(*R*)-*N*-(4-(2-amino-3-mercaptopropanamido)phenyl)-4-(4-(3,3-dimethylbutanamido)benzamido)benzamide (**L-CysAA**): Compound **8** (0.4 mmol) and TIPS (1.2 mmol) were mixed in TFA (25 mL) and dichloromethane (25 mL). The solution was then refluxed at room temperature for 24 h. Afterwards, the volatile components were removed under vacuum, and the product was precipitated by adding diethyl ether (100 mL). The solid precipitate was filtered, washed with diethyl ether excessively, and dried under vacuum to yield the product (yield: 99%). ^1^H NMR (400 MHz, DMSO-*d*_6_): δ = 7.96 (m, 6H), 7.77 (d, 4H), 7.59 (d, 2H), 4.07 (t, 1H), 3.02 (m, 2H), 2.25 (s, 2H), 1.05 (s, 9H) ppm. ^13^C NMR (400 MHz, DMSO-*d*_6_): δ = 171.0, 166.3, 165.6, 165.2, 142.9, 142.8, 136.0, 134.0, 129.8, 129.2, 129.1, 128.9, 121.3, 120.3, 119.8, 118.8, 55.5, 50.1, 47.4, 44.6, 35.8, 31.3, 30.1, 25.9 ppm. MS (LC/MS) [M + H]^+^ m/z calculated 548.2; [M + H]^+^ found 547.2.

**Synthesis of D-SerAA and L-SerAA**

******

**Supplementary Figure 5 |** Synthesis scheme to obtain the aramid amphiphile with D-chirality serine derivative head group (D-SerAA).

*tert*-butyl(*R*)-(3-(*tert*-butoxy)-1-((4-(4-(4-(3,3-dimethylbutanamido)benzamido)benzamido)phenyl)amino)-1-oxopropan-2-yl)carbamate (**9**): Compound **6** (0.22 mmol), Boc-D-Ser(tBu)-OH (0.22 mmol), EDC (0.67 mmol), HOBT (0.67 mmol), and DIPEA (0.89 mmol, 0.087 g) were dissolved in dimethylformamide (6 mL) and dichloromethane (4 mL). The mixture was stirred at room temperature for 24 h. After evaporating the organic solvents, the remaining residue was washed with DI water and methanol. The product was the recrystallized in acetonitrile, collected by vacuum filtration to yield the product (yield: 62%). ^1^H NMR (400 MHz, DMSO-*d*_6_): δ = 7.96 (m, 6H), 7.73 (q, 4H), 7.57 (d, 2H), 6.74 (d, 1H), 4.20 (m, 1H), 3.52 (m, 2H), 2.25 (s, 2H), 1.39 (s, 9H), 1.11 (s, 9H), 1.05 (s, 9H) ppm.

(*R*)-*N*-(4-(2-amino-3-hydroxypropanamido)phenyl)-4-(4-(3,3-dimethylbutanamido)benzamido)benzamide (**D-SerAA**): TFA (11 mmol) and TIPS (1.36 mmol) were added to a stirred solution of Compound **9** (0.13 mmol) in dichloromethane (10 mL) and stirred at room temperature for 24 h. After the reaction, the organic solvents were removed under vacuum. The product was precipitated in diethyl ether. The precipitate was obtained by filtration and then washed excessively with diethyl ether. The product was dried under vacuum to yield the final product (yield: 65%). ^1^H NMR (400 MHz, DMSO-*d*_6_): δ = 8.23 (s, 1H), 7.96 (m, 6H), 7.76 (q, 4H), 7.59 (d, 2H), 5.60 (t, 1H), 3.99 (t, 1H), 3.85 (s, 2H), 2.25 (s, 2H), 1.05 (s, 9H) ppm. ^13^C NMR (400 MHz, DMSO-*d*_6_): δ = 171.0, 165.7, 165.6, 165.2, 143.0, 142.8, 135.9, 134.3, 129.8, 129.2, 129.1, 128.9, 121.3, 120.2, 119.8, 118.8, 60.8, 55.5, 50.1, 47.4, 44.6, 35.8, 31.4, 30.1 ppm. MS (LC/MS) [M + H]^+^ m/z calculated 532.3; [M + H]^+^ found 532.2.

******

**Supplementary Figure 6 |** Synthesis scheme to obtain the aramid amphiphile with L-chirality serine derivative head group (L-SerAA).

(9*H*-fluoren-9-yl)methyl(*S*)-(3-(*tert*-butoxy)-1-((4-(4-(4-(3,3-dimethylbutanamido)benzamido)benzamido)phenyl)amino)-1-oxopropan-2-yl)carbamate (**10**): Compound **6** (0.22 mmol), Fmoc-*O*-*tert*-butyl-L-serine (0.45 mmol), EDC (0.67 mmol), HOBT (0.67 mmol), and DIPEA (0.89 mmol) were dissolved in dimethylformamide (6 mL) and dichloromethane (4 mL). The mixture was stirred at room temperature for 24 h. After the reaction, the solvent was evaporated under vacuum. The crude product was washed with DI water and methanol. The product was then purified by reprecipitation from acetonitrile, collected by vacuum filtration to yield the product (yield: 75%). ^1^H NMR (400 MHz, DMSO-*d*_6_): δ = 7.93 (m, 10H), 7.73 (q, 6H), 7.57 (q, 4H), 7.37 (m, 2H) 6.74 (d, 1H), 4.27 (m, 1H), 3.56 (m, 2H), 2.25 (s, 2H), 1.31 (s, 9H), 1.05 (s, 9H) ppm.

(*S*)-*N*-(4-(2-amino-3-hydroxypropanamido)phenyl)-4-(4-(3,3-dimethylbutanamido)benzamido)benzamide (**L-SerAA**): A solution of Compound **10** (0.17 mmol) was prepared in dichloromethane (7 mL). Piperidine (3 mL) was dropwise into the solution, and the mixture was stirred at room temperature for 2 h. After the reaction, the solvent was evaporated under vacuum and the residue was recrystallized in diethyl ether. The recrystallized powder was then dissolved in dichloromethane (10 mL) again. TFA (13 mmol) and TIPS (1.36 mmol) was added to the solution, which was stirred at room temperature for 24 h. After the reaction, the final product was recrystallized in diethyl ether and collected by vacuum filtration to yield the product (yield: 60%). ^1^H NMR (400 MHz, DMSO-*d*_6_): δ = 8.22 (s, 1H), 7.96 (m, 6H), 7.75 (q, 4H), 7.59 (d, 2H), 5.59 (s, 1H), 3.98 (s, 1H), 3.85 (s, 2H), 2.25 (s, 2H), 1.05 (s, 9H) ppm. ^13^C NMR (400 MHz, DMSO-*d*_6_): δ = 171.0, 165.7, 165.6, 165.2, 143.0, 142.8, 135.9, 134.2, 129.8, 129.2, 129.1, 128.9, 121.3, 120.2, 119.8, 118.8, 60.7, 55.5, 50.1, 47.4, 44.6, 35.8, 31.4, 30.1 ppm. MS (LC/MS) [M + H]^+^ m/z calculated 532.3; [M + H]^+^ found 532.2.

**Synthesis of GlyAA**

**Supplementary Figure 7 |** Synthesis scheme to obtain the aramid amphiphile with glycine derivative head group (GlyAA).

*tert*-butyl (2-((4-(4-(4-(3,3-dimethylbutanamido)benzamido)benzamido)phenyl)amino)-2-oxoethyl)carbamate (**11**): Compound **6** (1.88 mmol), Boc-Gly-OH (5.71 mmol), EDC (5.71 mmol), HOBT (5.71 mmol), and DIPEA (5.71 mmol, 0.738 g) were dissolved in dimethylformamide (50 mL) and dichloromethane (50 mL). The mixture was stirred at room temperature for 24 h. After evaporating the organic solvents, the remaining residue was washed with DI water, dichloromethane and methanol, and collected by vacuum filtration to yield the product (yield: 69%). ^1^H NMR (400 MHz, DMSO-*d*_6_): δ = 7.96 (m, 6H), 7.73 (q, 4H), 7.56 (d, 2H), 7.04 (t, 1H), 3.72 (d, 2H), 2.25 (s, 2H), 1.41 (s, 9H), 1.05 (s, 9H) ppm.

2-((4-(4-(4-(3,3-dimethylbutanamido)benzamido)benzamido)phenyl)amino)-2-oxoethan-1-aminium (**GlyAA**): Compound **11** (1.30 mmol) and TIPS (13 mmol) were mixed in TFA (10 mL) and dichloromethane (90 mL). The solution was then refluxed at room temperature for 24 h. Afterwards, the volatile components were removed under vacuum, and the product was precipitated by adding diethyl ether (300 mL). The solid precipitate was filtered, washed with diethyl ether, and 50 mL dichloromethane, and then dried under vacuum to yield the product (yield: 87%). ^1^H NMR (400 MHz, DMSO-*d*_6_): δ = 7.96 (m, 6H), 7.76 (d, 4H), 7.57 (d, 2H), 3.79 (s, 2H), 2.25 (s, 2H), 1.05 (s, 9H) ppm. ^13^C NMR (400 MHz, DMSO-*d*_6_): δ = 171.0, 165.7, 165.2, 165.0, 142.9, 142.8, 135.8, 134.3, 129.8, 129.2, 129.1, 128.9, 121.5, 119.9, 118.8, 50.1, 41.5, 31.4, 30.1 ppm. MS (LC/MS) [M + H]^+^ m/z calculated 502.6; [M + H]^+^ found 502.3.


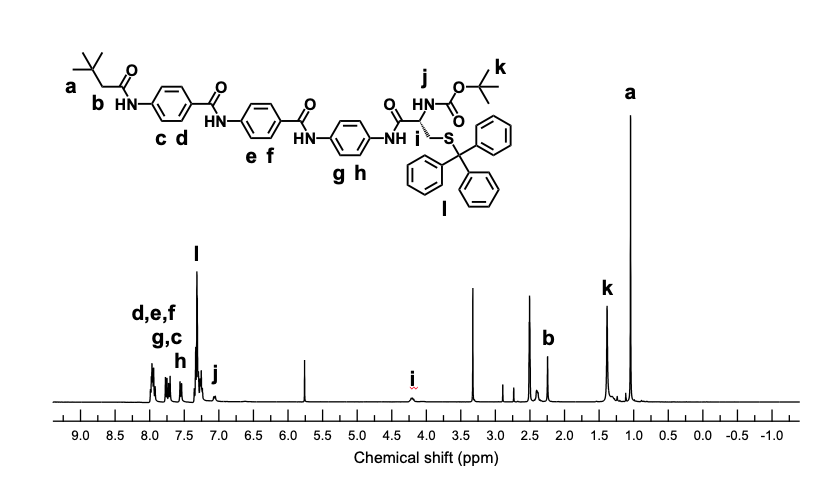


**Supplementary Figure 8 |** ^1^H NMR spectra of compound **7**.


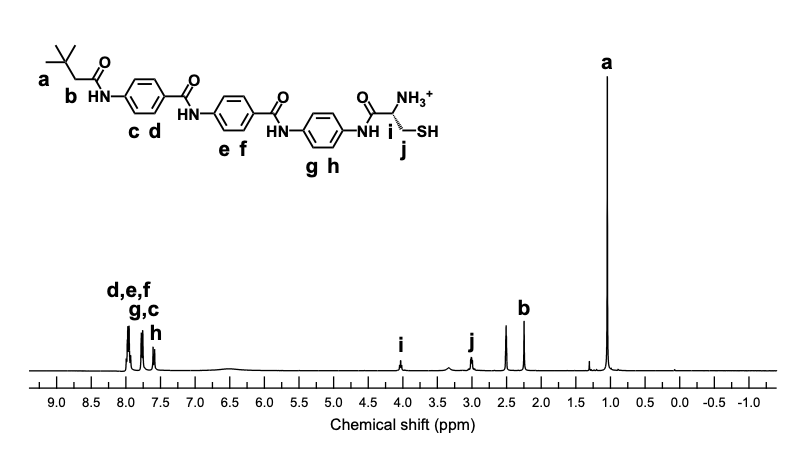


**Supplementary Figure 9 |** ^1^H NMR spectra of **D-CysAA** compound.

**
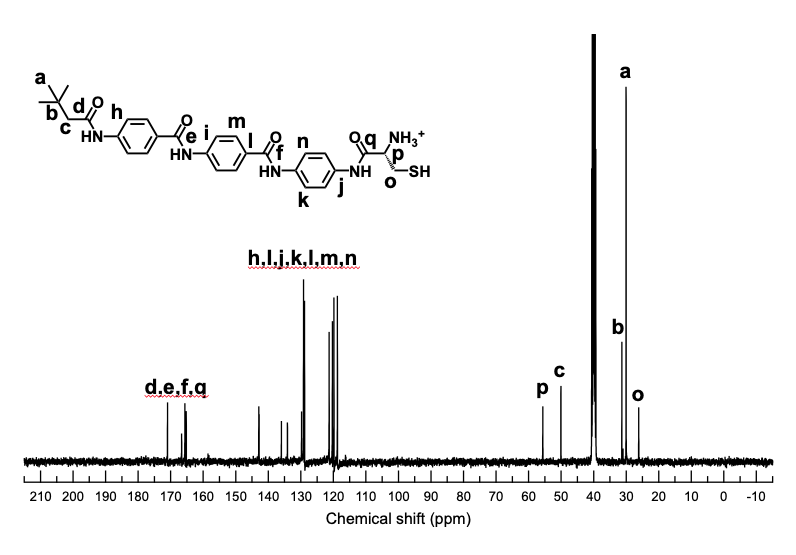
**

**Supplementary Figure 10 |** ^13^C NMR spectra of **D-CysAA** compound.

**
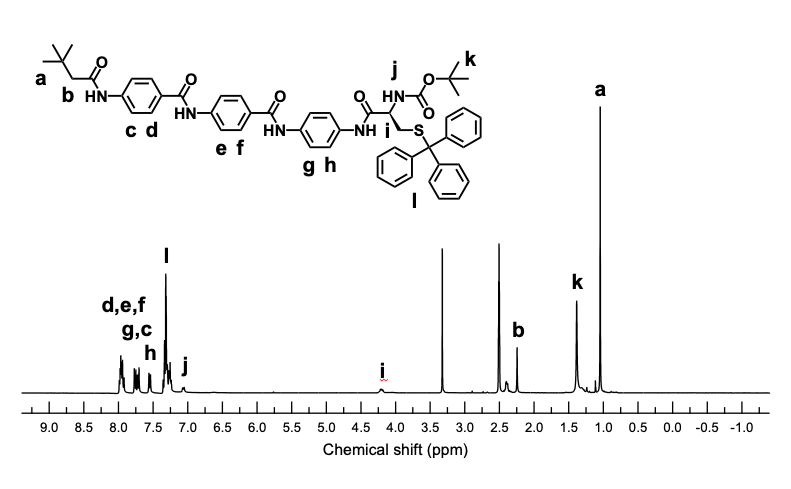
**

**Supplementary Figure 11 |** ^1^H NMR spectra of compound **8**.

**
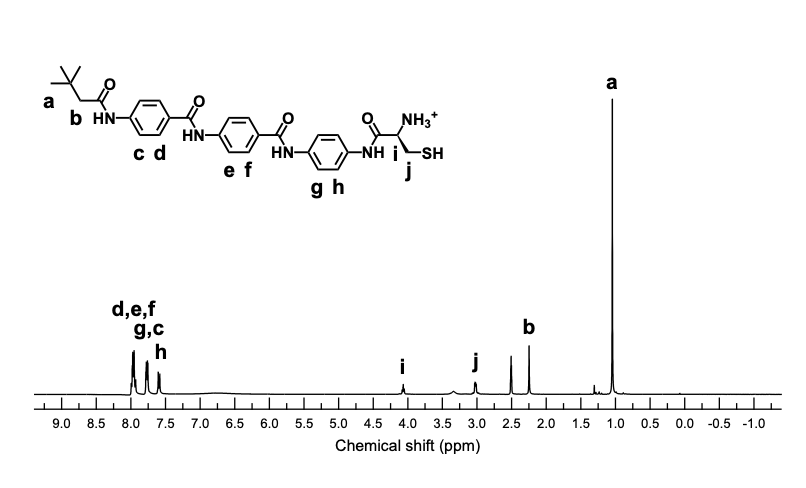
**

**Supplementary Figure 12 |** ^1^H NMR spectra of **L-CysAA** compound.

**
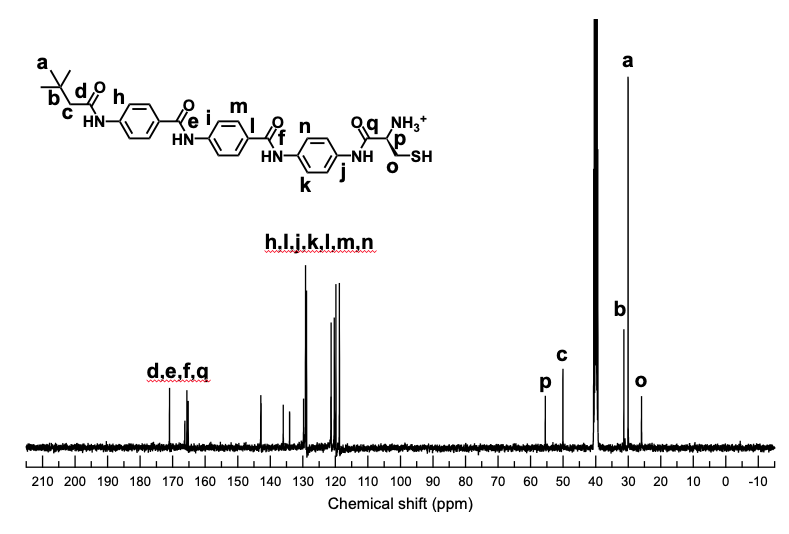
**

**Supplementary Figure 13 |** ^13^C NMR spectra of **L-CysAA** compound.

**
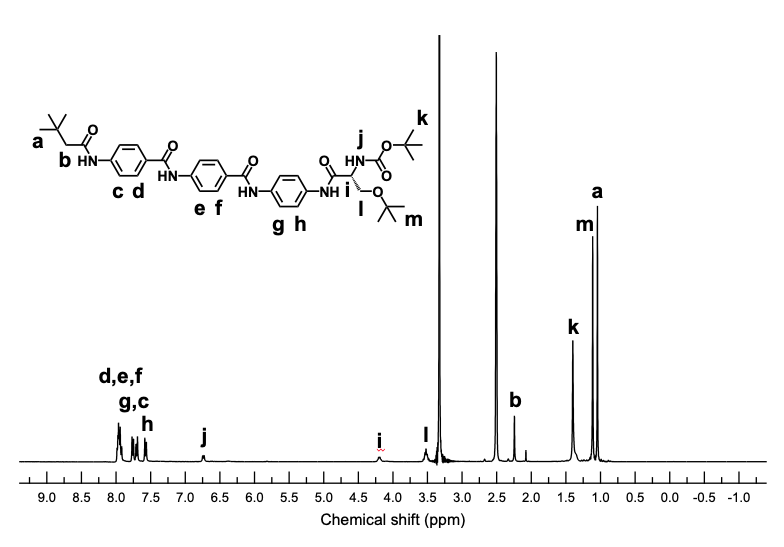
**

**Supplementary Figure 14 |** ^1^H NMR spectra of compound **9**.

**
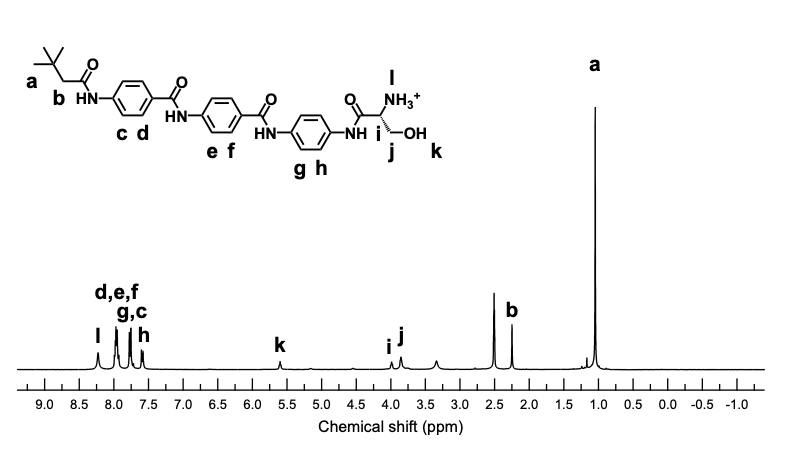
**

**Supplementary Figure 15 |** ^1^H NMR spectra of **D-SerAA** compound.

**
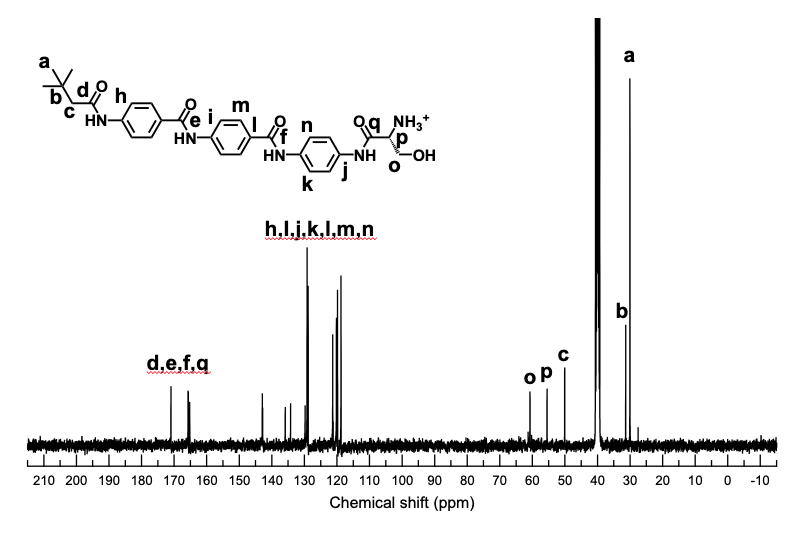
**

**Supplementary Figure 16 |** ^13^C NMR spectra of **D-SerAA** compound.

**
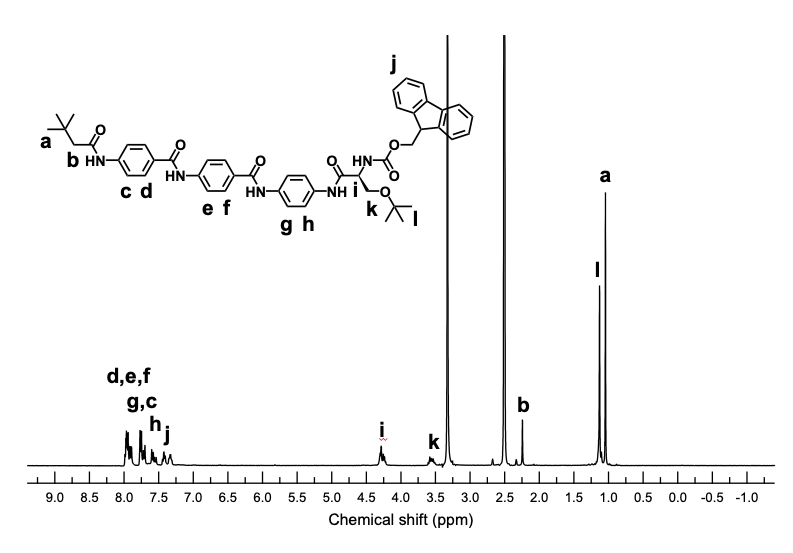
**

**Supplementary Figure 17 |** ^1^H NMR spectra of compound **10**.

**
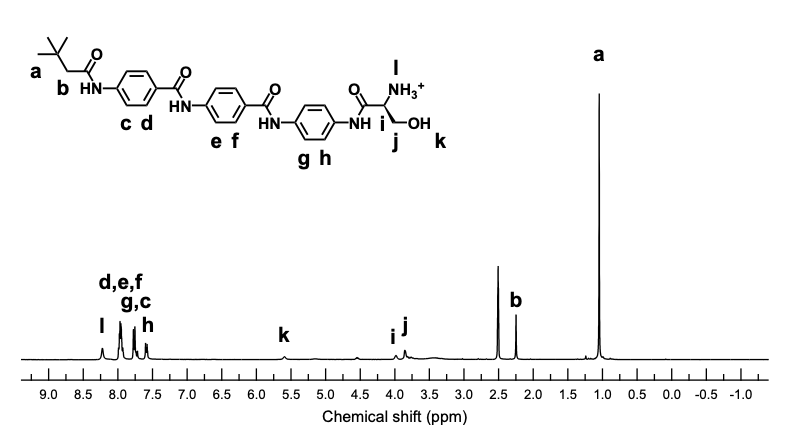
**

**Supplementary Figure 18 |** ^1^H NMR spectra of **L-SerAA** compound.


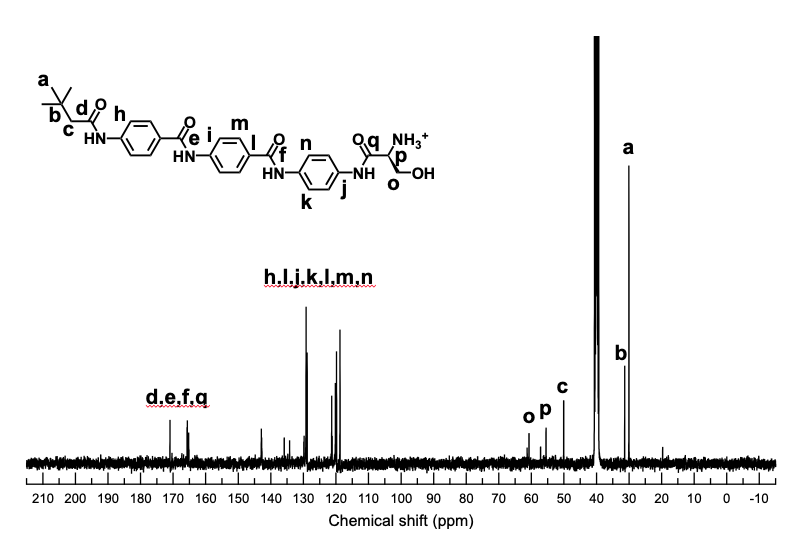


**Supplementary Figure 19 |** ^13^C NMR spectra of **L-SerAA** compound.

**
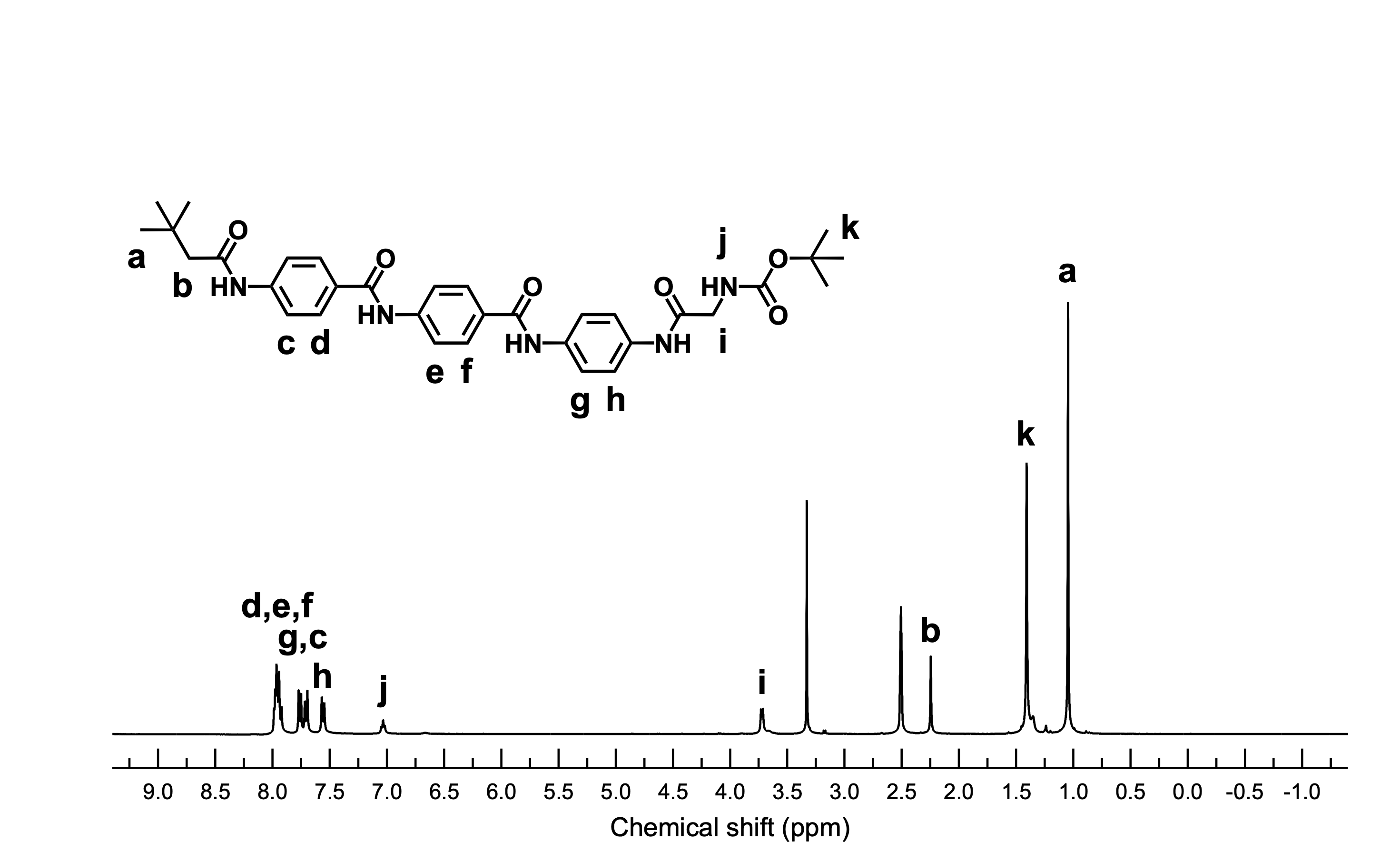
**

**Supplementary Figure 20 |** ^1^H NMR spectra of compound **11**.

**
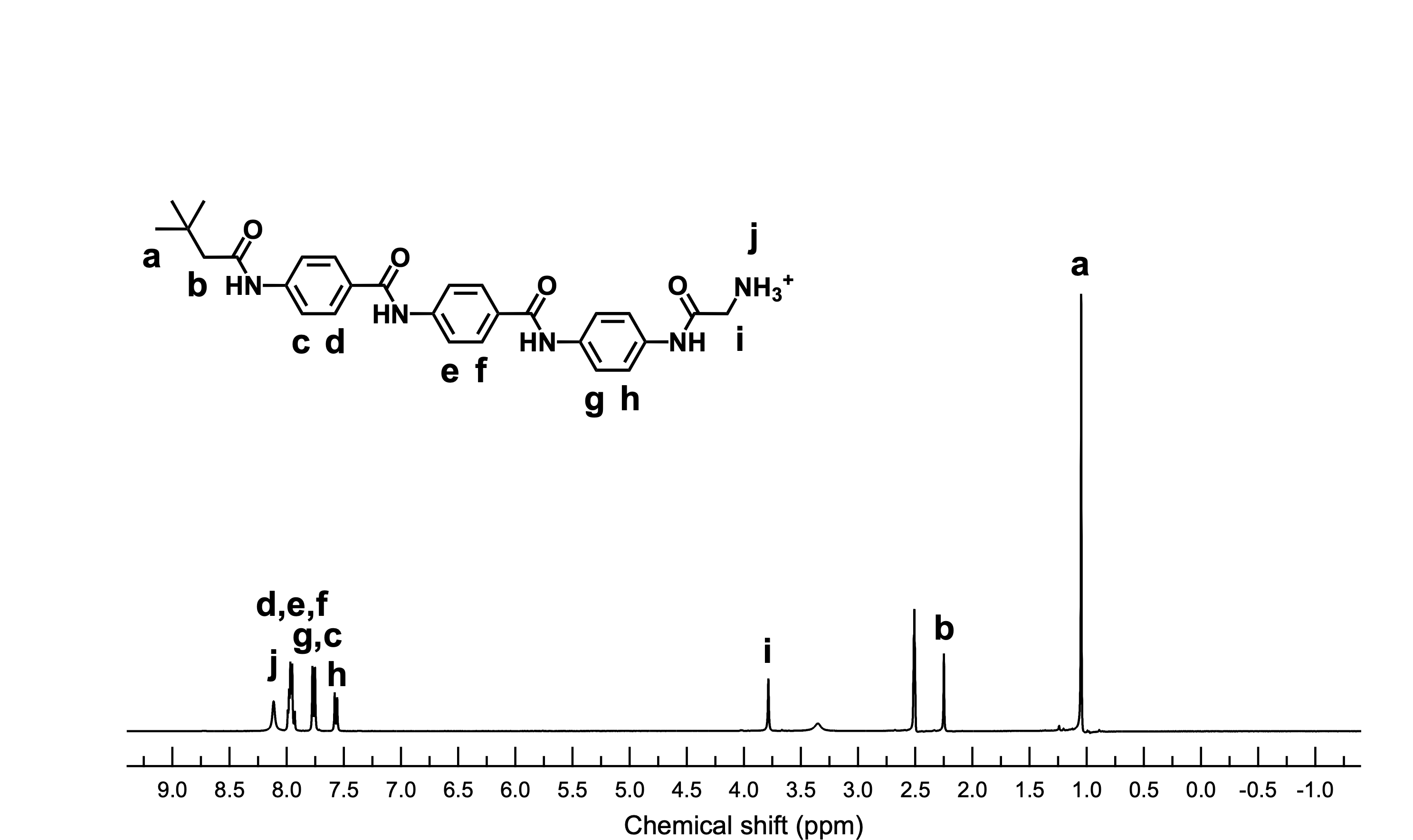
**

**Supplementary Figure 21 |** ^1^H NMR spectra of **GlyAA** compound.


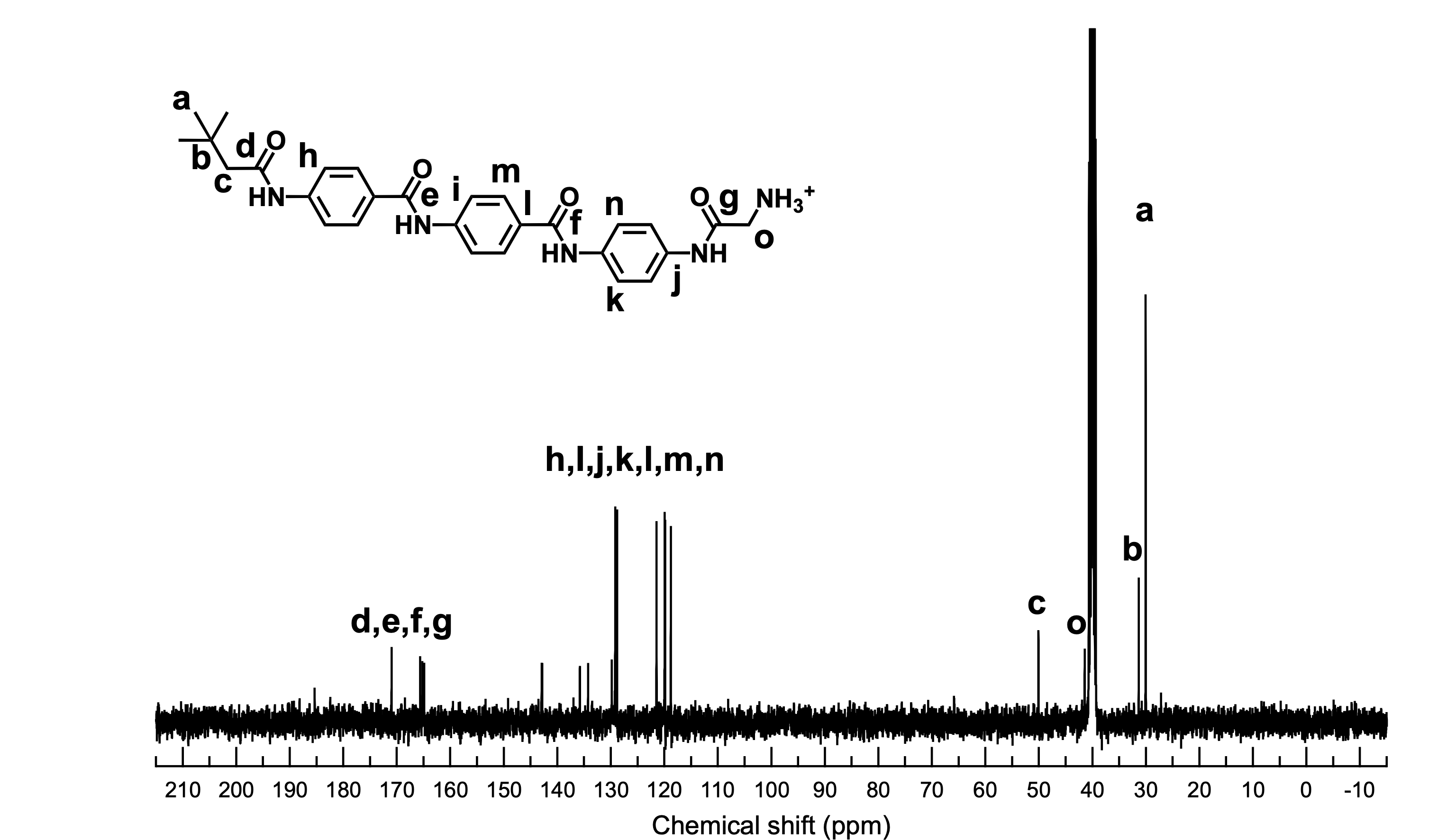


**Supplementary Figure 22 |** ^13^C NMR spectra of **GlyAA** compound.

**Supplementary Note 3:** TEM of AA nanostructures

**
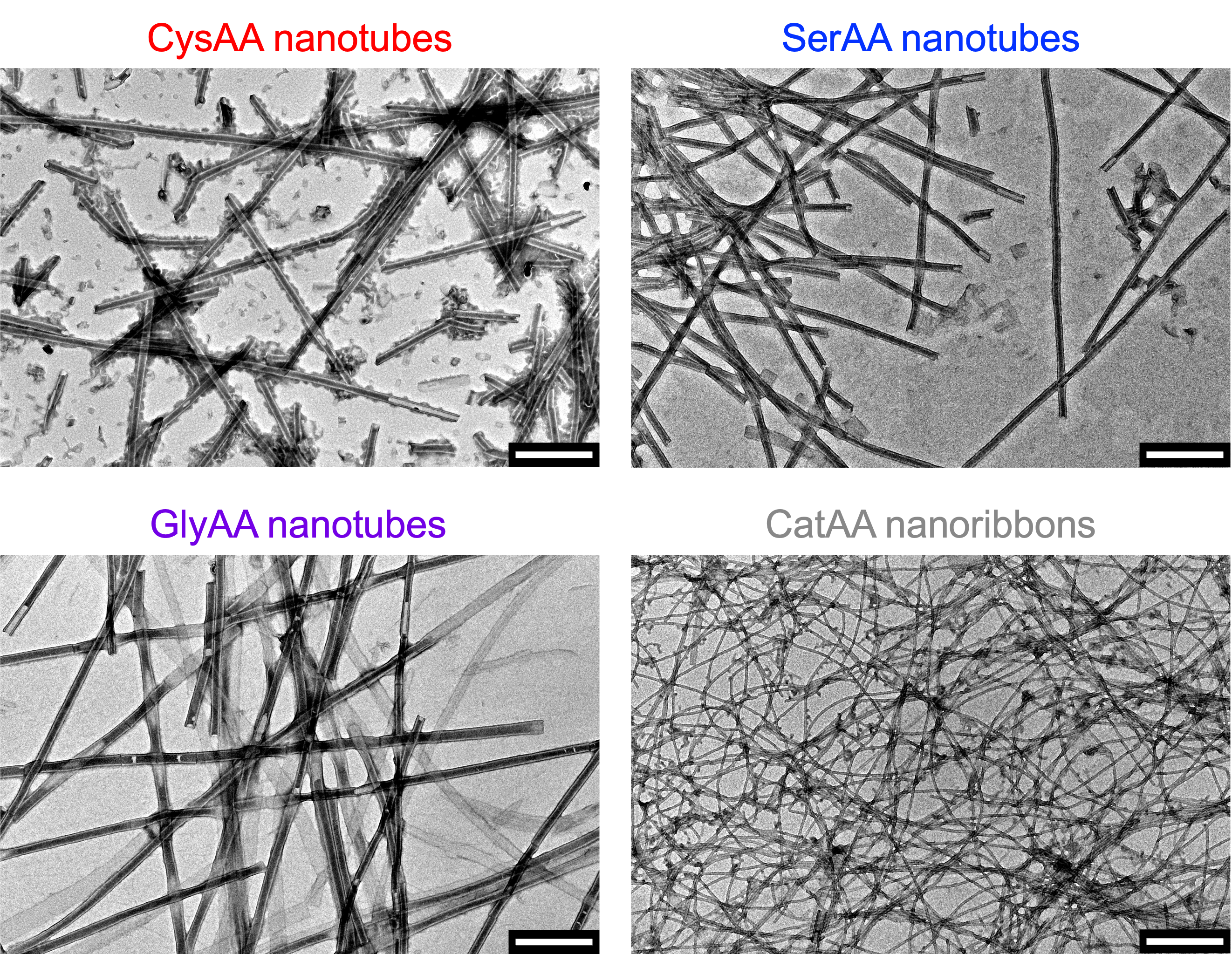
**

**Supplementary Figure 23 |** Representative conventional TEM images show the morphological difference between CysAA, SerAA, and GlyAA nanotubes and CatAA nanoribbons. Scale bars in all TEM images represent 300 nm.

**Supplementary Note 4:** Influence of temperature on CysAA self-assembly

The self-assembly morphology of CysAA nanostructures in water depends on the equilibration temperature. While the formation of both nanotubes and nanoribbons competes from room temperature to moderately elevated temperatures, CysAAs exclusively self-assemble into nanotubes at an 80°C equilibration. This trend of transition is well captured by conventional TEM (Supplementary Figure 24), and the increase in the peak at the lower wavelength in UV-vis spectra also corroborates our reported observation of AA nanostructures transitioning from nanoribbons to nanotubes (Supplementary Figure 25).^[11,12]^


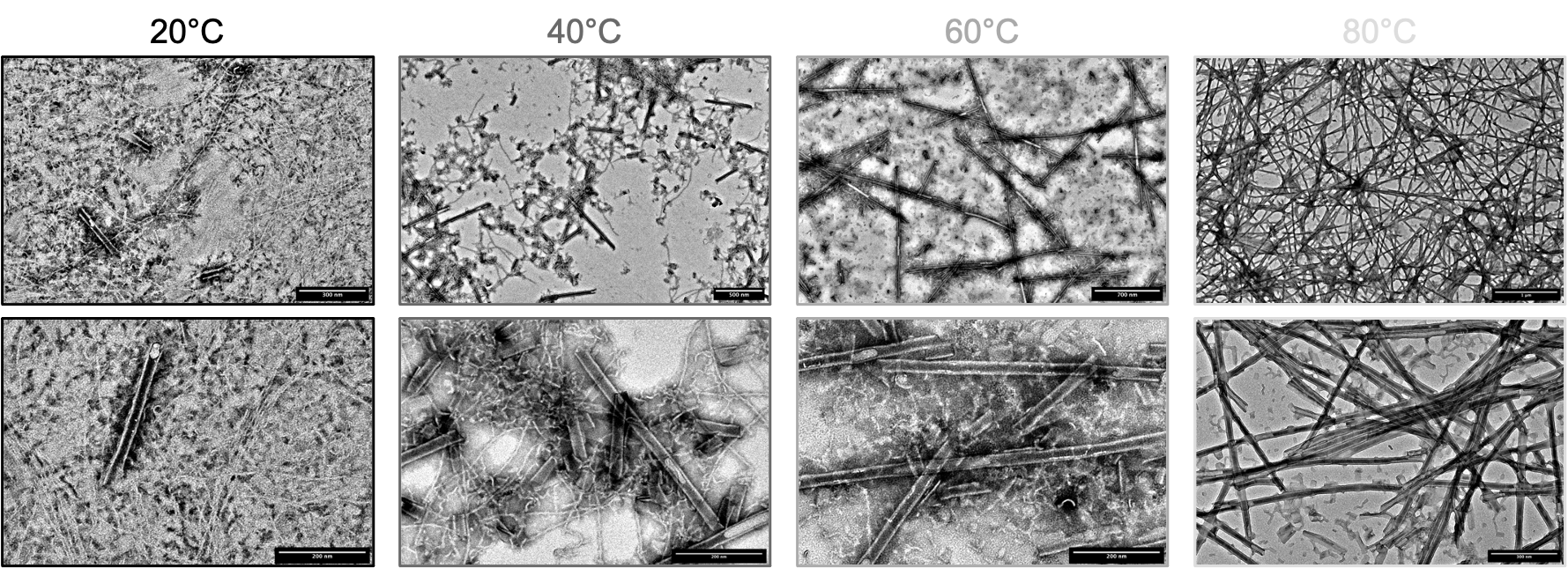


**Supplementary Figure 24 |** Representative conventional TEM images of CysAA nanostructures self-assembled at various temperatures for 24 h, with the concentration of CysAA fixed at 1 mg/mL. While CysAAs form a mixture of nanotubes and nanoribbons at 20°C, the proportion of nanotubes increases as the annealing temperature rises from 40°C to 60°C. By 80°C, the CysAAss exclusively self-assemble into nanotubes.


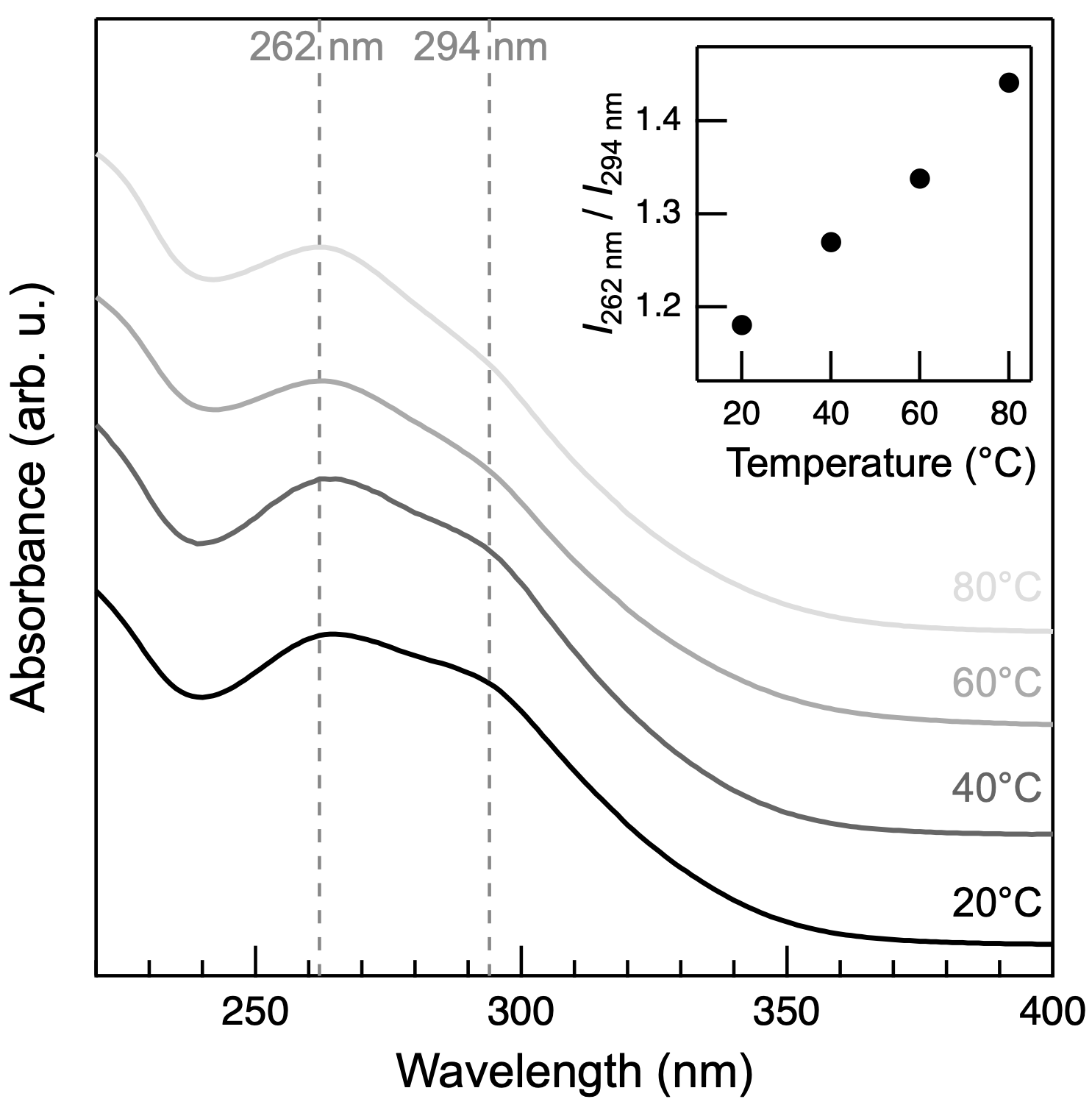


**Supplementary Figure 25 |** Temperature-dependent UV-vis spectra of CysAA nanostructures self-assembled at various temperatures for 24 h, with a consistent concentration of 1 mg/mL CysAAs. The peak at lower wavelength (262 nm) is primarily associated with molecular interactions in AA nanotubes, while the peak at higher wavelength (294 nm) primarily corresponds to interactions in AA nanoribbons. Inset: The temperature dependence of the peak intensity ratio between 262 nm and 294 nm demonstrates a linear relationship, reflecting the transition from a mixed state to predominantly nanotubes as the annealing temperature increases. All spectra are normalized based on the intensity at 294 nm.

**Supplementary Note 5:** Influence of pH on CysAA self-assembly

The self-assembly morphologies of CysAA nanostructures in water can be altered by pH conditions, especially in acidic environments. Conventional TEM images (Supplementary Figure 26), UV-vis absorption, and X-ray scattering (Supplementary Figure 27) reveal that the CysAA nanotubes unravel into nanofibers as the concentration of HCl increases.


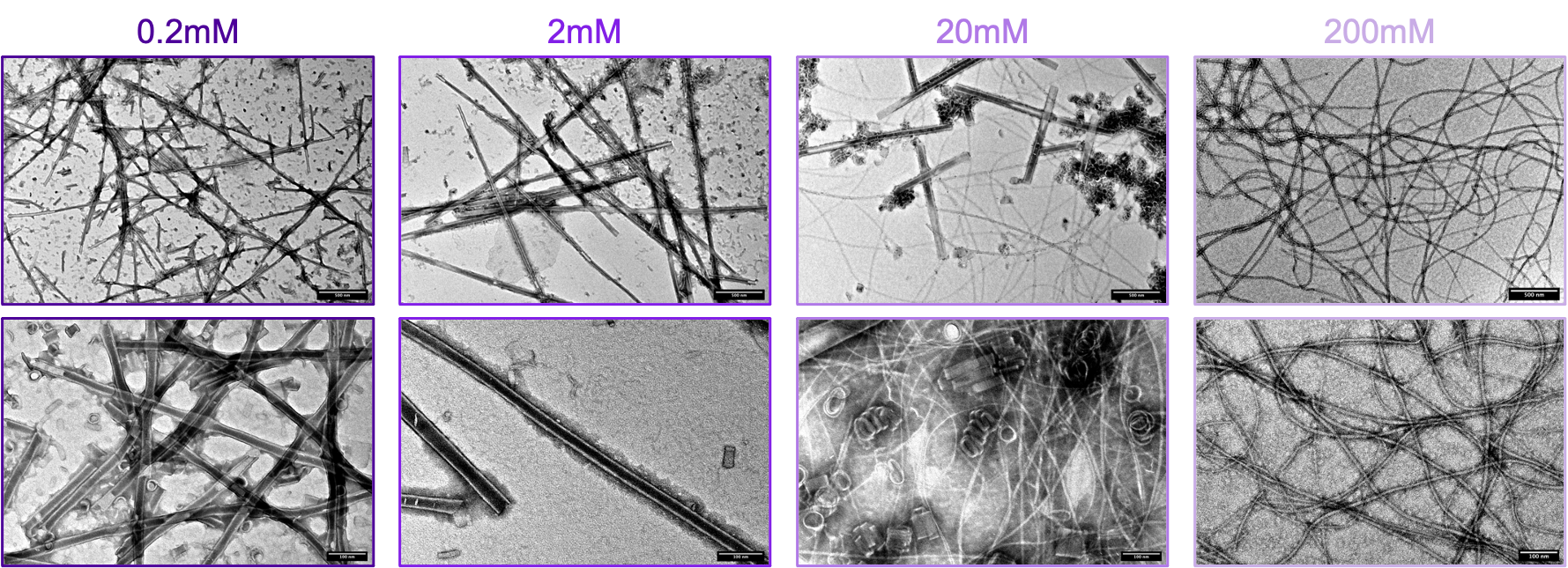


**Supplementary Figure 26 |** Representative conventional TEM images of CysAA nanostructures self-assembled in various concentration of HCl solution at 80°C for 24 h, with the concentration of CysAA fixed at 1 mg/mL. With the increasing concentration of HCl, CysAA nanotubes transition to nanofibers after equilibration. Notably, the CysAA nanotubes exhibit an intermediate state of unraveling into nanofibers at the critical concentration of a 20mM HCl solution.


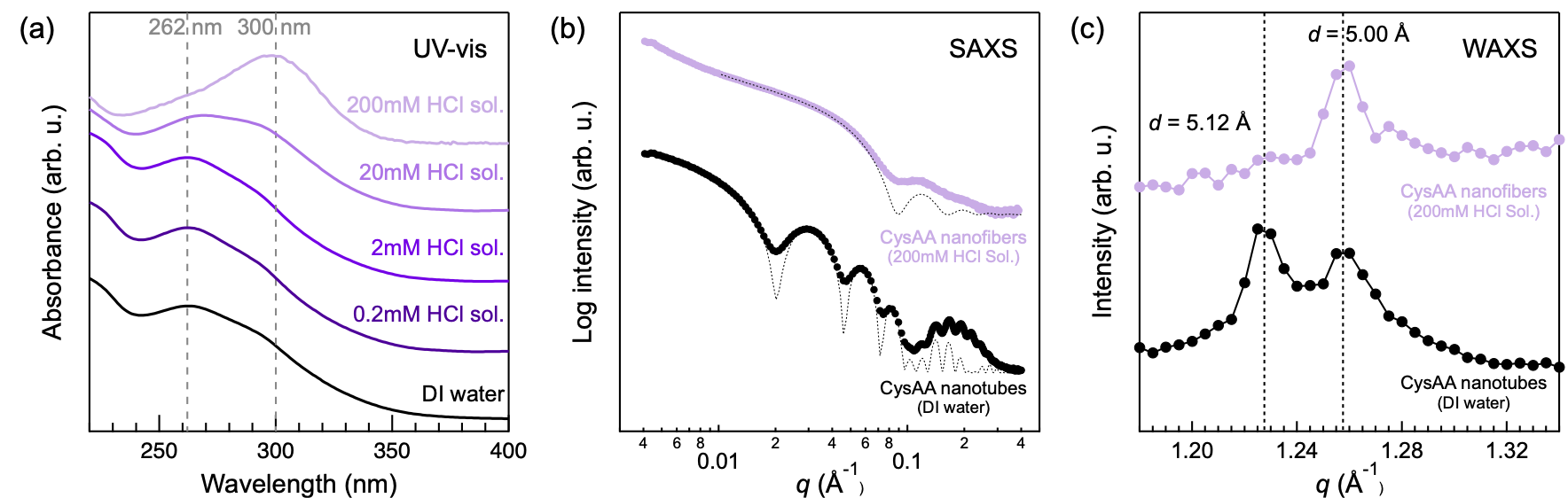


**Supplementary Figure 27 |** (a) pH dependence of UV-vis spectra for CysAA nanostructures self-assembled in various concentrations of HCl solution at 80°C for 24 h, with the concentration of CysAA fixed at 1 mg/mL. The peak primarily associated with AA nanotubes (262 nm) decreases, while the peak primarily associated with AA nanofibers (300 nm) emerges as the HCl concentration increases. (b) Small-angle X-ray scattering (SAXS) of CysAA nanotubes (prepared in DI water) and nanofibers (prepared in 200 mM HCl solution) shows distinct features of one-dimensional nanostructures. Details of the fitting are explained later in Supplementary Figure 28. (c) Wide-angle X-ray scattering (WAXS) of CysAA nanotubes and nanofibers reveals the same characteristic peak corresponding to the long-range ordering of the hydrogen bonding network (*d* = 5.00 Å). Additionally, the CysAA nanotubes present another peak at *d* = 5.12 Å, indicating a different packing arrangement compared to nanoribbons.

**Supplementary Note 6:** Influence of concentration on CysAA self-assembly

In contrast to temperature and pH, the self-assembly of CysAA nanostructures presents a subtle impact when varying the concentration of CysAA from 1 mg/mL to 10 mg/mL.


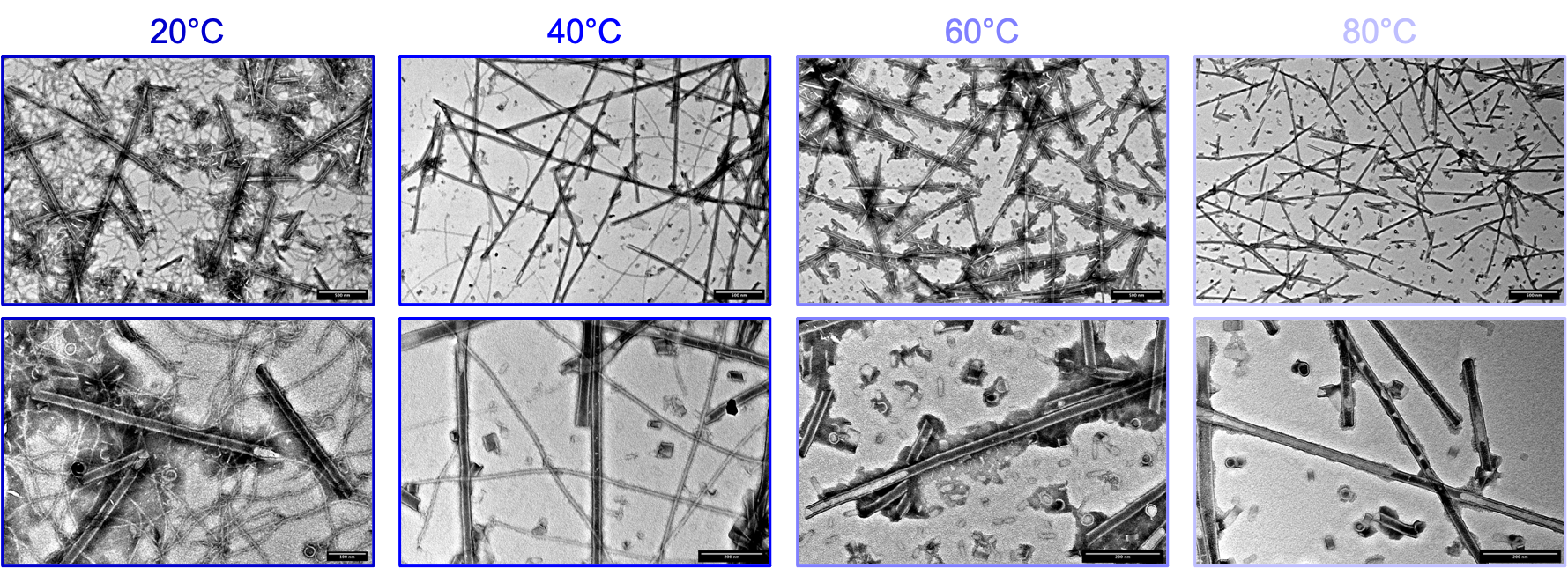


**Supplementary Figure 28 |** Representative conventional TEM images of CysAA nanostructures self-assembled at various temperatures for 24 h, with the concentration of CysAA fixed at 5 mg/mL. The observed trend, where the formation of both nanotubes and nanoribbons competes from room temperature to moderately elevated temperatures before CysAAs exclusively self-assemble into nanotubes at an 80°C equilibration, is similar to that observed in 1 mg/mL samples.


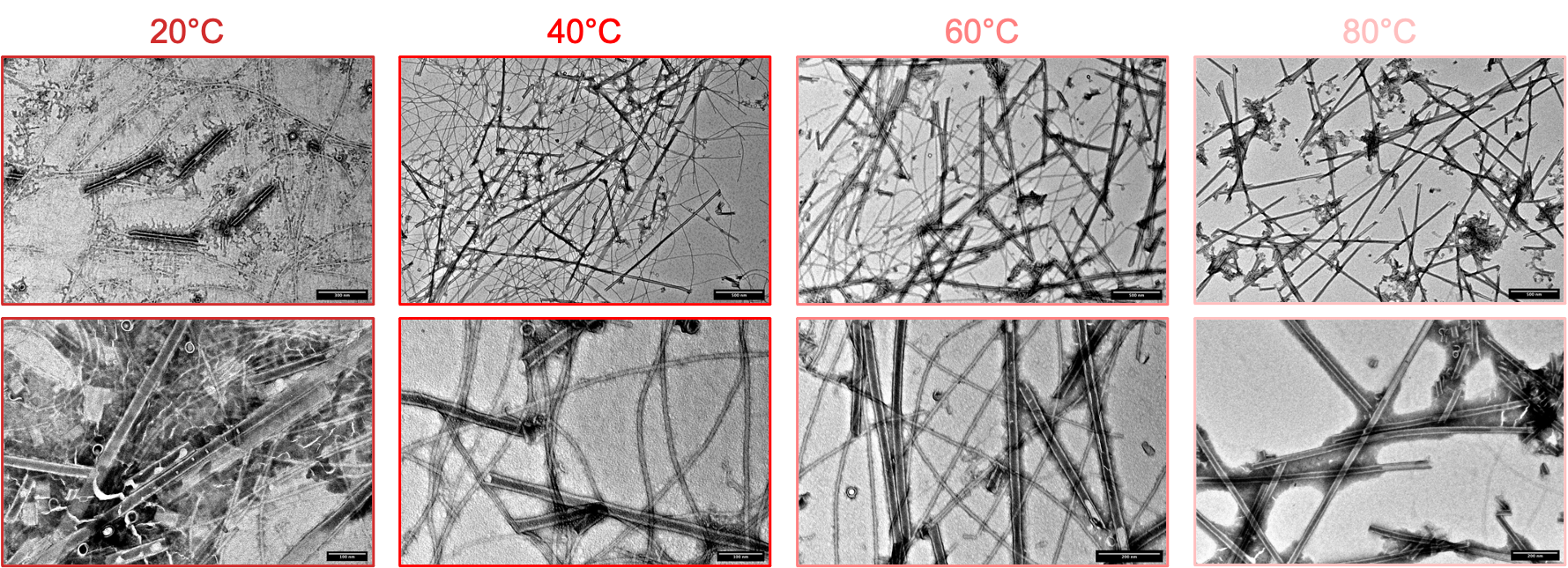


**Supplementary Figure 29 |** Representative conventional TEM images of CysAA nanostructures self-assembled at various temperatures for 24 h, with the concentration of CysAA fixed at 10 mg/mL. The observed trend, where the formation of both nanotubes and nanoribbons competes from room temperature to moderately elevated temperatures before CysAAs exclusively self-assemble into nanotubes at an 80°C equilibration, is similar to that observed in 1 mg/mL samples.


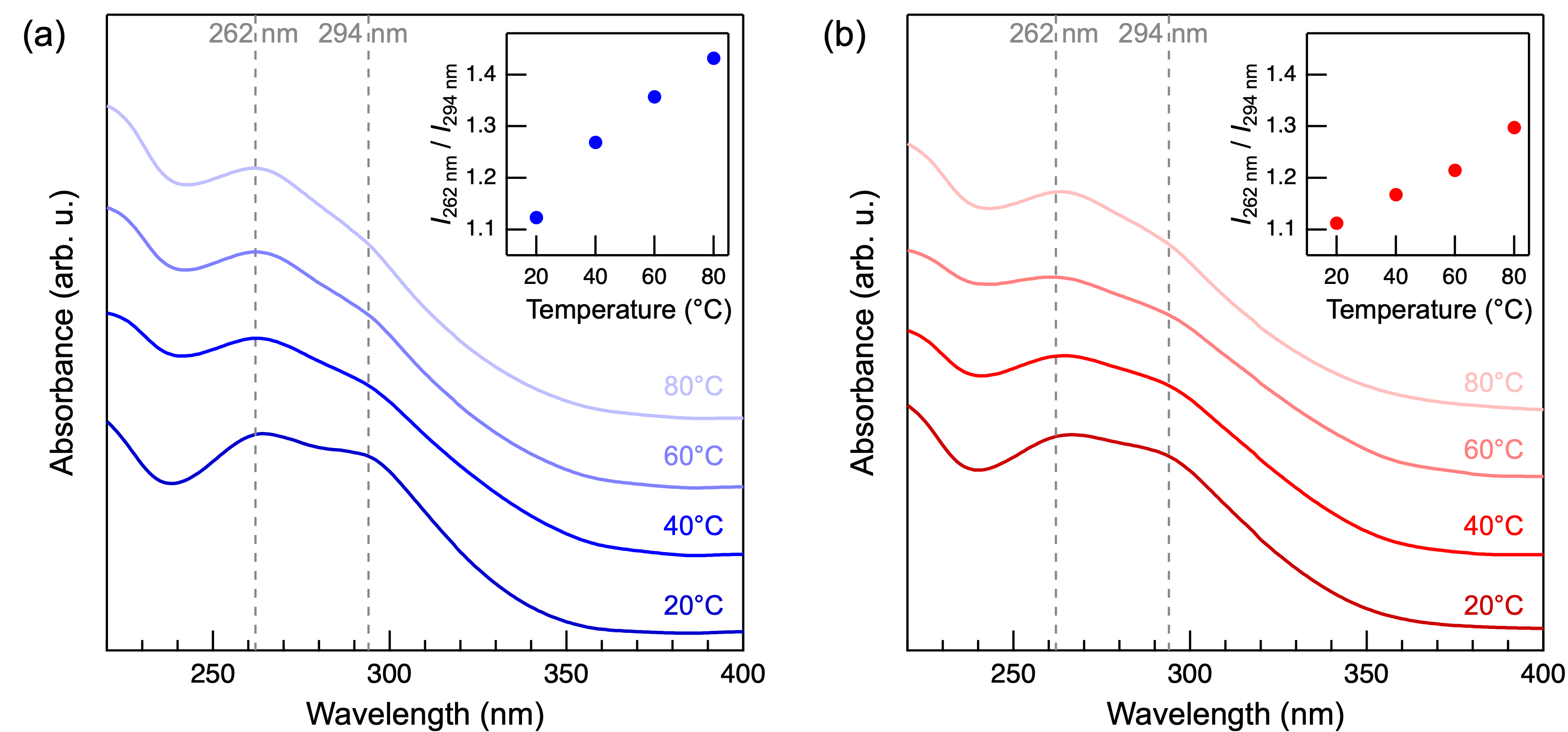


**Supplementary Figure 30 |** Temperature-dependent UV-vis spectra of CysAA nanostructures self-assembled at various temperatures for 24 h, with a consistent concentration of CysAAs at (a) 5 mg/mL, and (b) 10 mg/mL. Inset: The temperature dependence of the peak intensity ratio between 262 nm and 294 nm demonstrates a linear relationship, reflecting the transition from a mixed state to predominantly nanotubes as the annealing temperature increases. All spectra are normalized based on the intensity at 294 nm.

**Supplementary Note 7:** Influence of surface chirality on CysAA self-assembly

In this work, we investigate the influence of chirality within the cysteine derivative head group domain of CysAAs by observing the self-assembly behavior of D-CysAAs and L-CysAAs. The surface chirality appears to have a negligible influence on the self-assembly of CysAA nanotubes. This suggests that molecular packing is predominantly governed by the aramid structural domain and aliphatic tail domain, rather than the hydrophilic domain, which has relatively high flexibility.


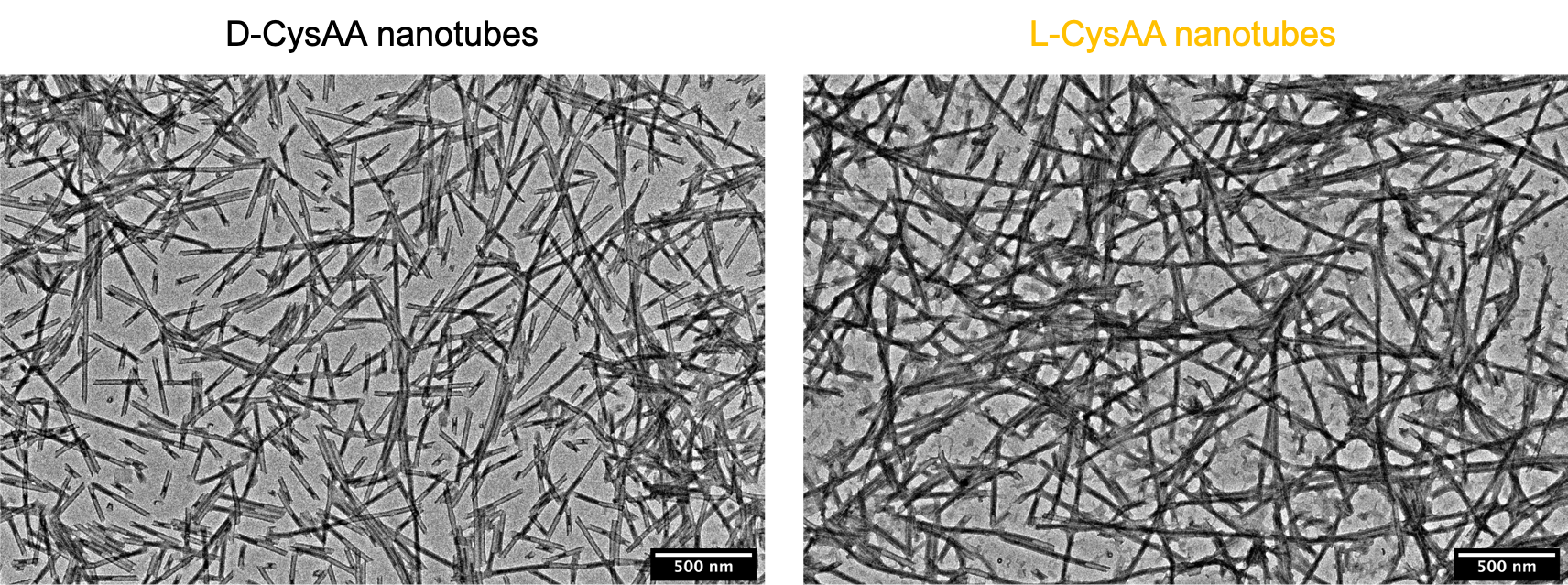


**Supplementary Figure 31 |** Representative conventional TEM images of D-CysAA and L-CysAA nanotubes self-assembled in DI water at 80°C for 24 h, with the concentration of CysAA fixed at 1 mg/mL.


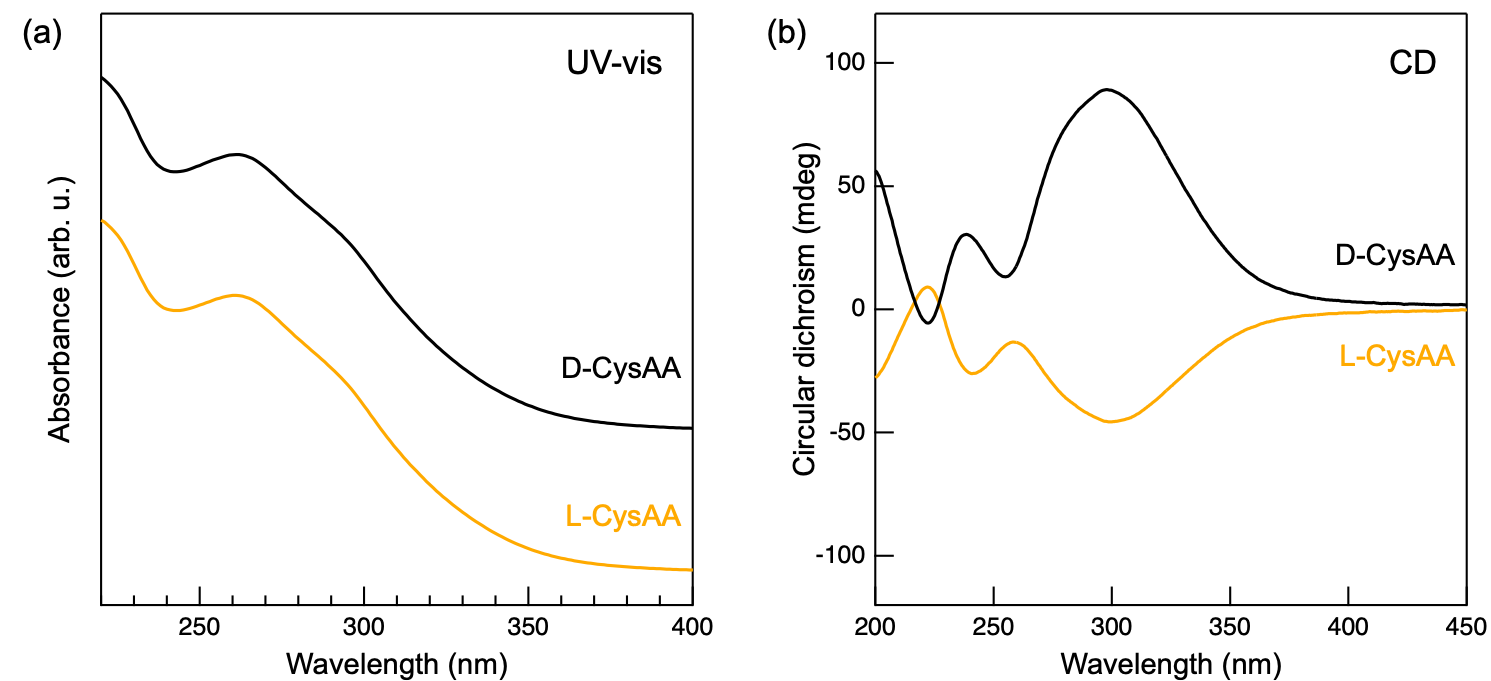


**Supplementary Figure 32 |** (a) UV-vis spectra of self-assembled D-CysAA and L-CysAA nanotubes show identical features. (b) Circular dichroism of D-CysAA and L-CysAA nanotubes shows opposite chiral signals, which are attributed solely to the chirality centers within the cysteine derivative head group domain.

**Supplementary Note 8:** cryo-TEM image of SerAA nanotubes


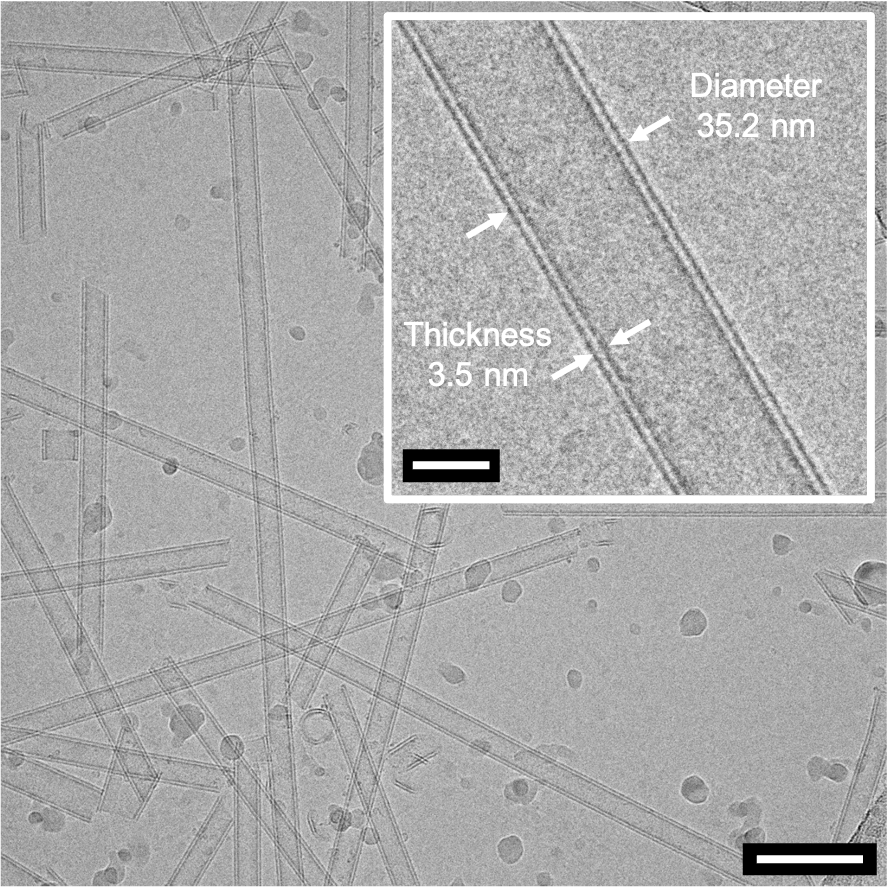


**Supplementary Figure 33 |** Representative cryo-TEM image of SerAA nanotubes in water exhibits a well-defined hollow tube structure with an exterior diameter (*D*) of 35.2 nm and a wall thickness (*t*) of 3.5 nm (scale bar, 100 nm, inset scale bar, 20 nm).

**Supplementary Note 9:** Small- and wide-angle X-ray scattering

The fitting of SAXS data was completed in SasView software to determine the morphology of several nanostructures. The profiles of CysAA and SerAA nanotubes from Figure 2c best fit a hollow cylinder model, and the profile of CysAA nanofibers from Supplementary Figure 21 best fit a flexible cylinder model. The fitting results of CatAA nanoribbons was reported in our previous work.^[9]^ The following parameters were used for the fits.

**
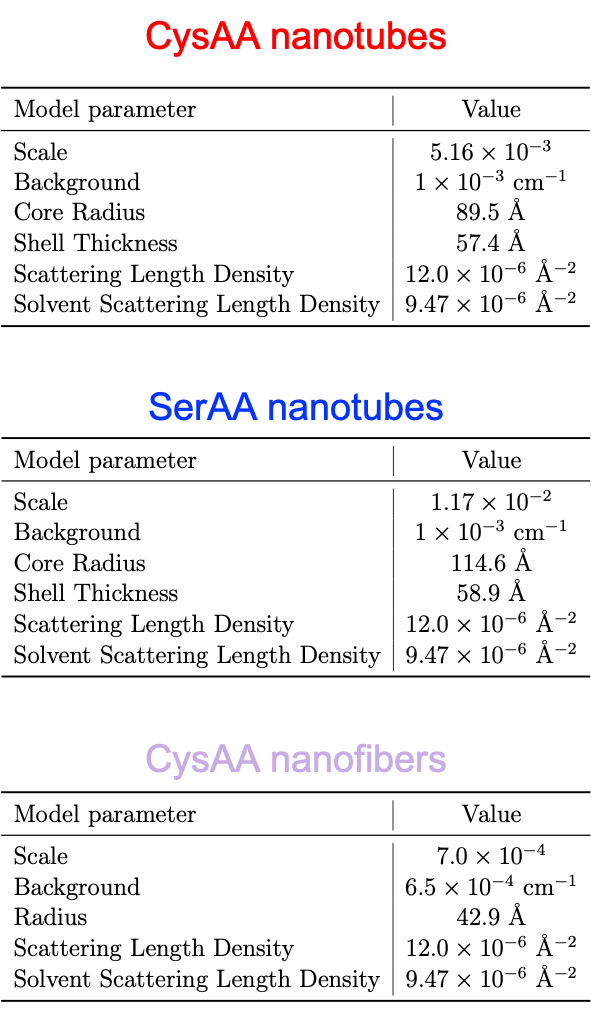
**

**Supplementary Figure 34 |** Parameters for fitting the SAXS of CysAA and SerAA nanotubes from Figure 2c to a hollow cylinder model, and the SAXS of CysAA nanofibers from Supplementary Figure 21 to a flexible cylinder model.


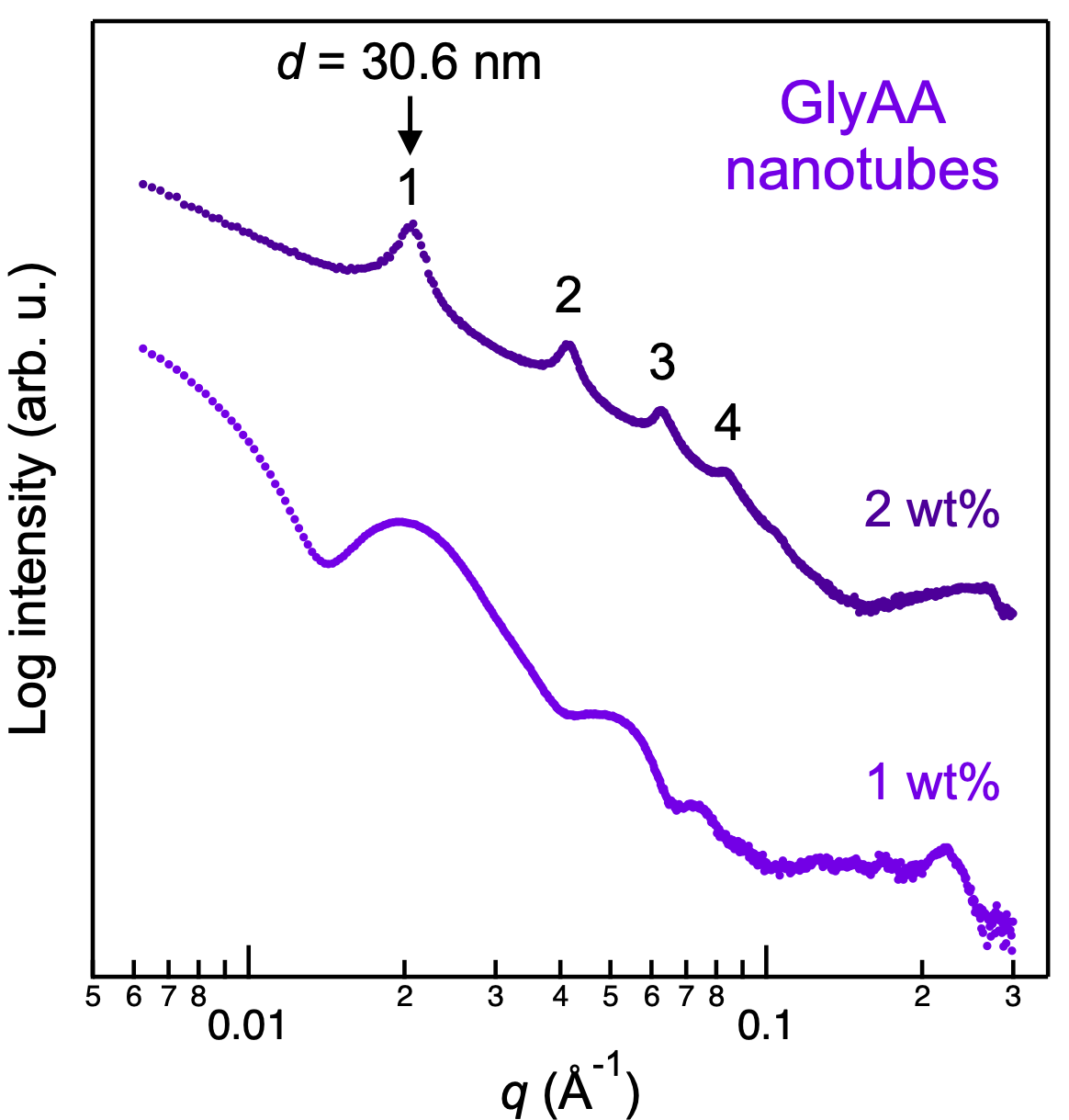


**Supplementary Figure 35 |** SAXS analysis of a 2% suspension of GlyAA nanotubes shows distinct peaks indicative of long-range ordering, likely attributed to nanotube stacking. In contrast, the 1% suspension exhibits a profile consistent with a hollow cylindrical morphology and significant flexibility.

**Supplementary Figure 36 |** WAXS of CysAA, SerAA, and GlyAA nanotubes, and CatAA nanoribbons. Distinct peaks between nanotubes and nanoribbon samples imply different molecular packing motifs with contrasting hydrogen bond networks.

**Supplementary Note 10:** Time dependence of CysAA self-assembly at 80°C


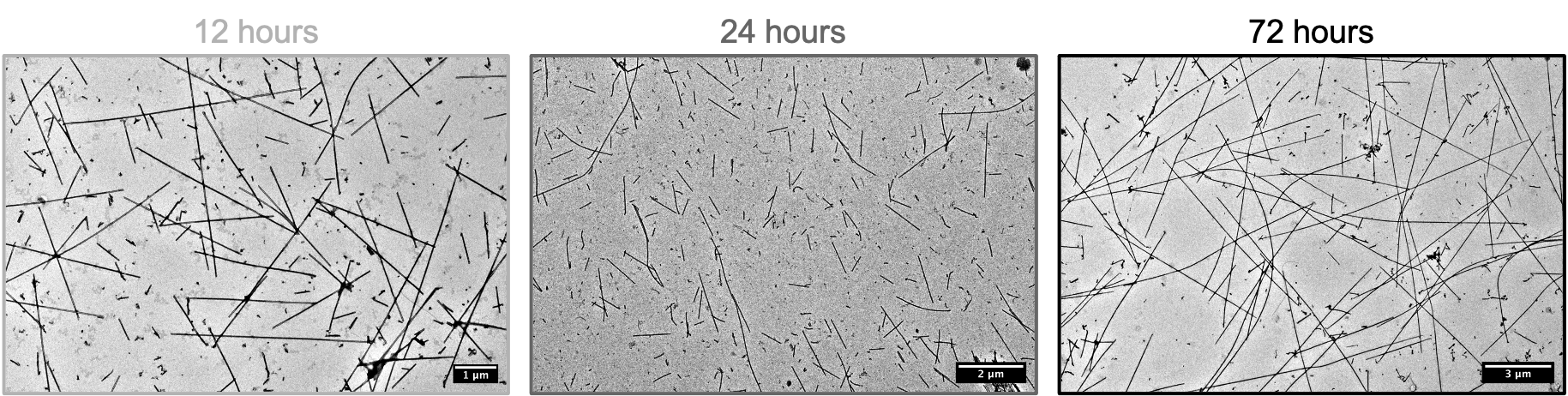


**Supplementary Figure 37 |** Representative conventional TEM images of CysAA nanotubes self-assembled at 80°C for various lengths of annealing time, with the concentration of CysAA fixed at 1 mg/mL. Statistical analysis of the length distribution was applied to the CysAA nanotubes prepared at different annealing times, with a sample size of n = 2000.

**Supplementary Table 3 |** Summary of central measures of CysAA nanotube length as a function of equilibration time. The arithmetic mean is the sum of all nanotube lengths divided by the total number of nanotubes. The geometric mean is the nth root of the product of all nanotube lengths. The median is the middle value when nanotube lengths are sorted in ascending order. The median from the log-normal distribution is obtained from the fitted equation.

**
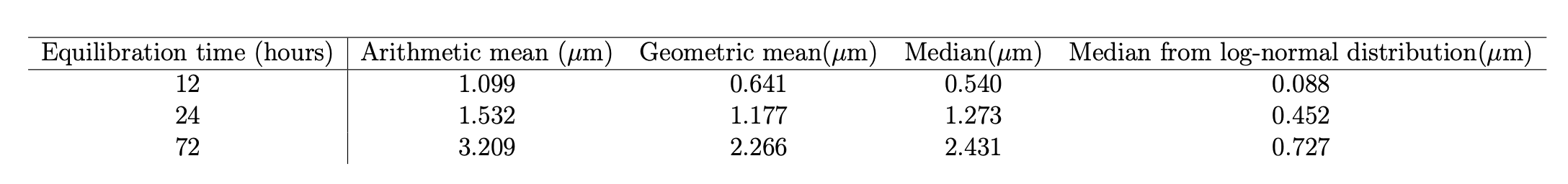
**

**
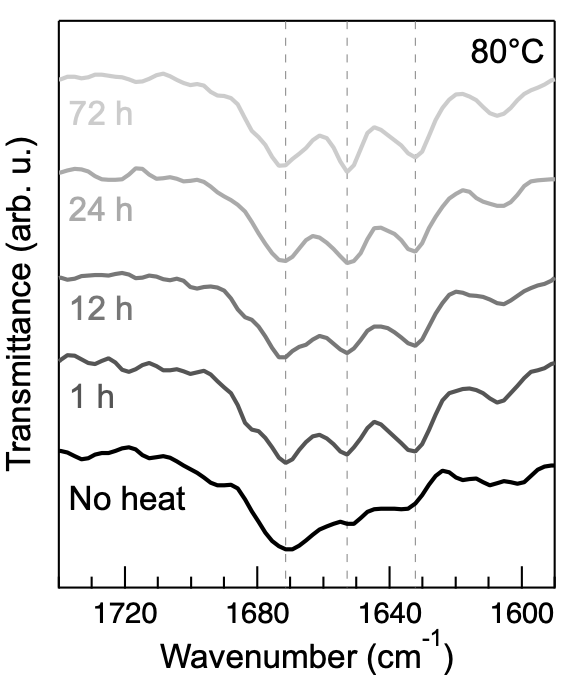
**

**Supplementary Figure 38 |** Time-dependent ATR-FTIR spectra for CysAA nanotubes at concentrations of 10 mg/mL in D_2_O. The peaks at 1653 and 1632 cm^-1^ emerge upon annealing at 80°C, consistent with the elongation of the length of CysAA nanotubes.

**Supplementary Note 11:** AFM images and mechanical analysis

Sample preparation: AA nanotube suspensions were diluted to a concentration of 45 µg/mL in DI water. Subsequently, 200 µL of the diluted nanotube suspension was deposited onto clean glass. For this purpose, we used 35-mm-diameter FluoroDish™ with a cover glass bottom (World Precision Instruments). The glass surface was initially cleaned using DI water and ethanol, followed by activation through ozone treatment. This was done prior to the application of the nanotube suspension onto the glass for a duration of 10 minutes. Afterward, the surfaces were gently rinsed with DI water and immediately subjected to analysis by atomic force microscopy (AFM) directly in the liquid.

Topological analysis: The persistence length of nanotubes was determined as described elsewhere.^[13,14]^ Briefly, we traced the contours of many nanotubes using parametric splines and used statistical mechanics to obtain the persistence length via analysis of shape fluctuations. Since the persistence lengths of our nanotubes looked higher than their contour lengths, we used the worm-like chain model to fit the mean square of the deviations *δ* from secant midpoints versus the secant length *L* according to the following equation: $<\delta^{2}> = {L^{3}}/{(48 P)}$ to obtain the persistence length *P*. To account for possible kinetic trapping of fibrils on the surface, we applied a correction factor of 1.5 to the calculated persistence length, which corresponds to calculating the midpoint fluctuations between 2D and 3D.^[12]^ The fractional dimension is then considered to be 2.5 ± 0.5, and this experimental uncertainty is propagated to the final error estimate.

**
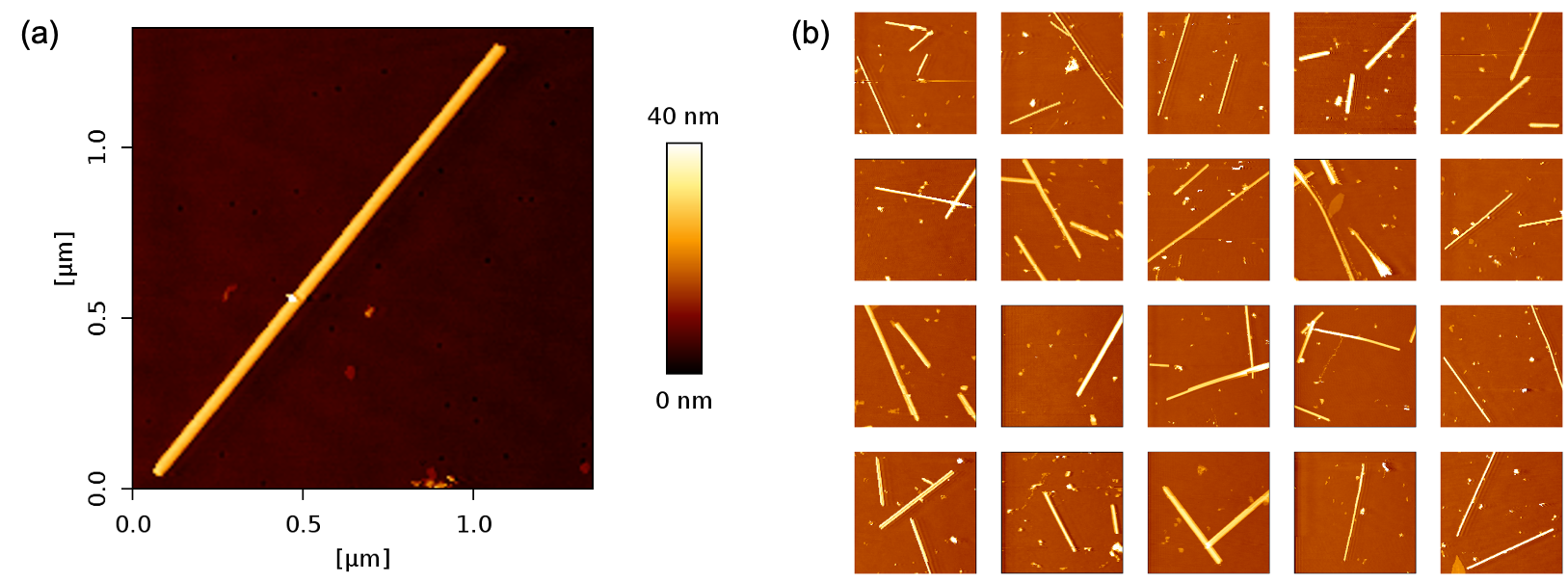
**

**Supplementary Figure 39 |** (a) A representative AFM profile of CysAA nanotubes deposited on mica indicates that the diameter of the nanotubes is approximately 30 nm, corroborating the dimensions obtained by SAXS analysis and cryo-TEM images. (b) Contour traces of AFM images of CysAA nanotubes, a representative set of which are shown here, were used for determining nanotube stiffness by statistical topographical analysis. Image dimensions are 2.5 μm × 2.5 μm.


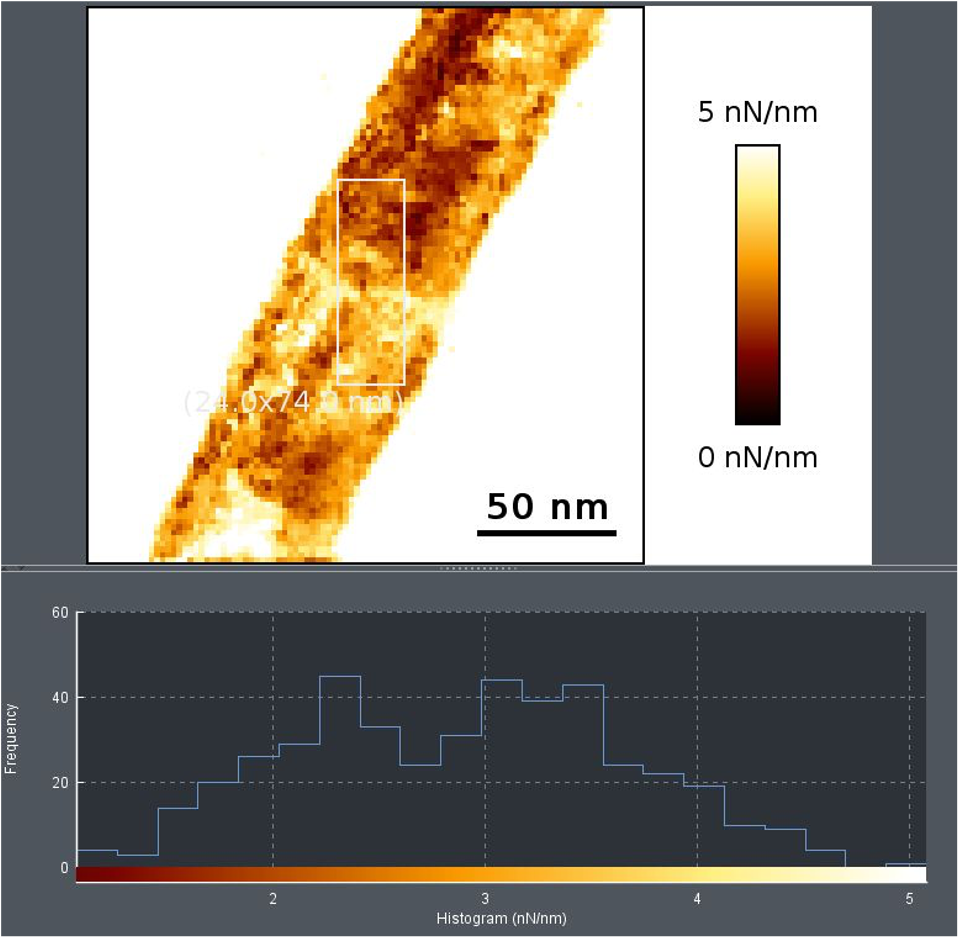


**Supplementary Figure 40 |** A representative mapping analysis of the stiffness distribution in the center of CysAA nanotubes shows an approximate average value of the stiffness to be around 3 N/m.


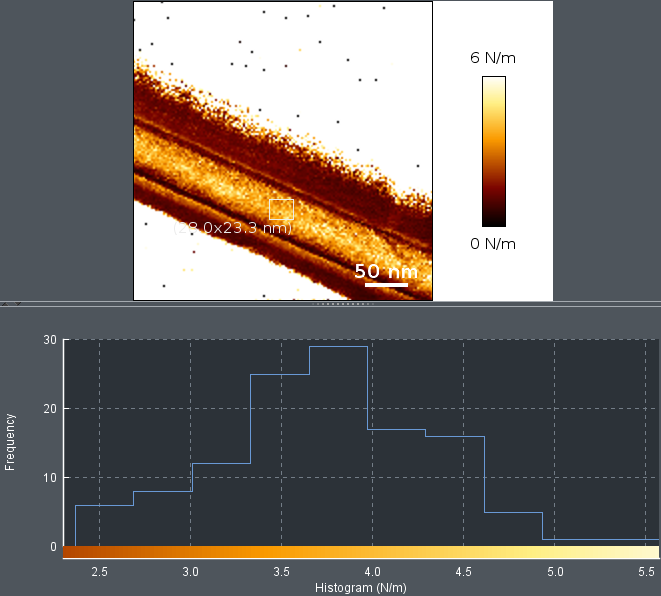


**Supplementary Figure 41 |** A representative mapping analysis of the stiffness distribution in the center of CysAA nanotubes after 2 years of storage in deionized water at room temperature, showing that the stiffness values and their distribution exhibit no noticeable changes compared to freshly prepared nanotubes.

**Supplementary Note 12:** CysAA nanotube stability under various conditions

Changes in the mechanical stiffness of CysAA nanotubes were evaluated by first depositing the nanotubes onto AFM cover glasses and then immersing the entire glass substrate in designated solutions for 24 hours. After treatment, the substrates were gently rinsed with deionized water and immediately analyzed by atomic force microscopy (AFM) directly in water.

We found that CysAA nanotubes treated with 2 M NaCl solution or absolute ethanol showed no change in stiffness. Treatment with 1 M NaOH caused a slight reduction in nanotube height and a corresponding increase in stiffness. Exposure of the nanotubes to 1 M HCl solution, tetrahydrofuran (THF), or dimethylformamide (DMF) resulted in complete removal of the CysAA nanotubes from the substrate. This removal is likely due to detachment from the glass surface in the case of HCl (supported by subsequent conventional TEM observations) and to nanotube disassembly in the case of organic solvents such as DMF.^[10]^


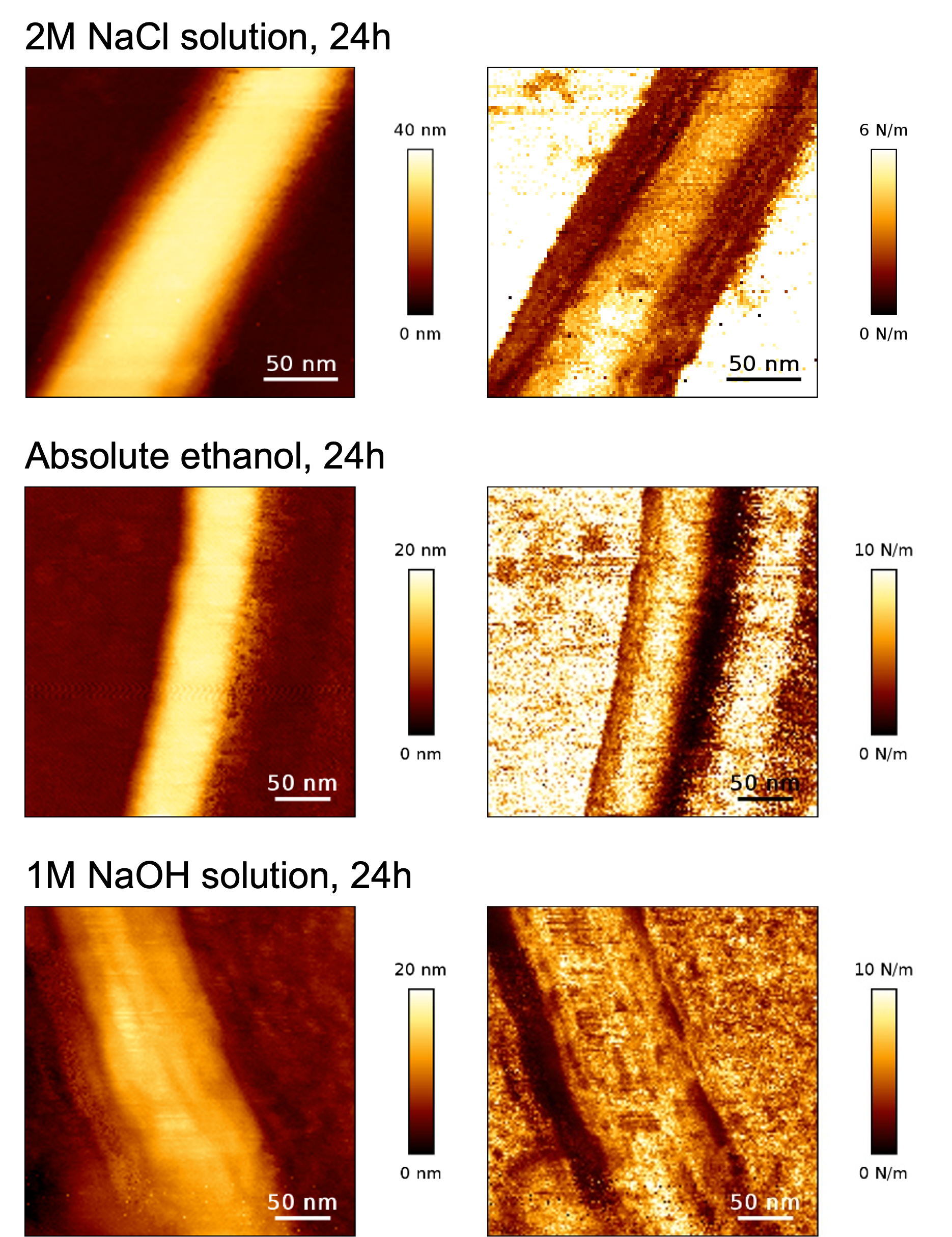


**Supplementary Figure 42 |** Representative AFM profiles and stiffness‑mapping analysis of CysAA nanotubes after treatment with 2 M NaCl, absolute ethanol, and 1 M NaOH for 24 hours.


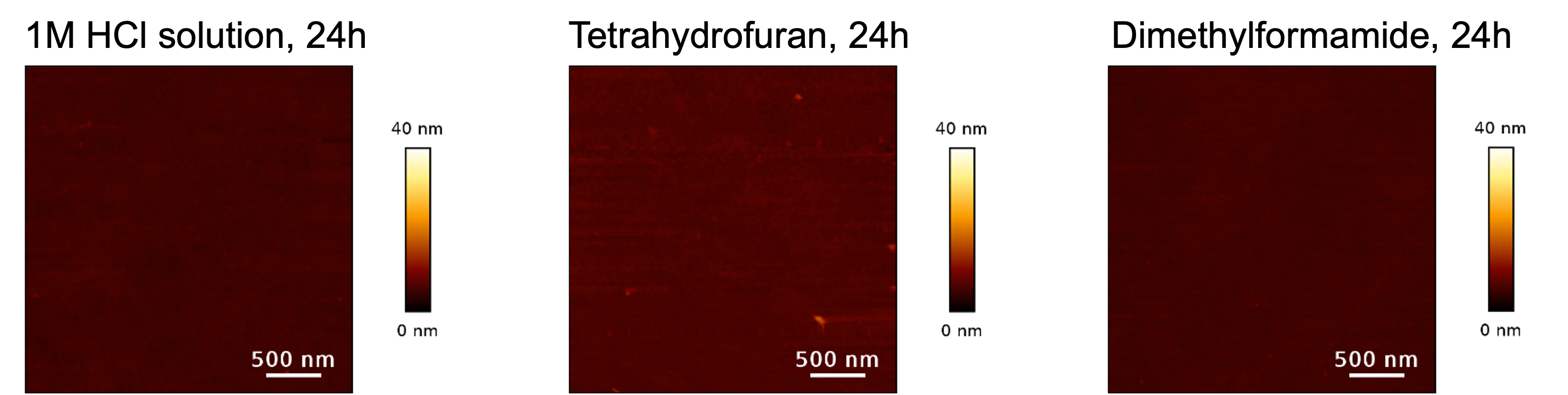


**Supplementary Figure 43 |** Representative AFM profiles of CysAA nanotubes after treatment with 1M HCl, THF, and DMF for 24 hours.

Additionally, we treated CysAA nanotube suspensions with various additives and evaluated their morphological stability using conventional TEM. We confirmed that treatment with 0.2 M HCl solution, a 1:1 molar ratio of oxidizing agent hydrogen peroxide (H₂O₂), or a 1:1 molar ratio of the typical reducing agents dithiothreitol (DTT) or tris(2‑carboxyethyl)phosphine (TCEP) did not noticeably alter the nanotube morphology.


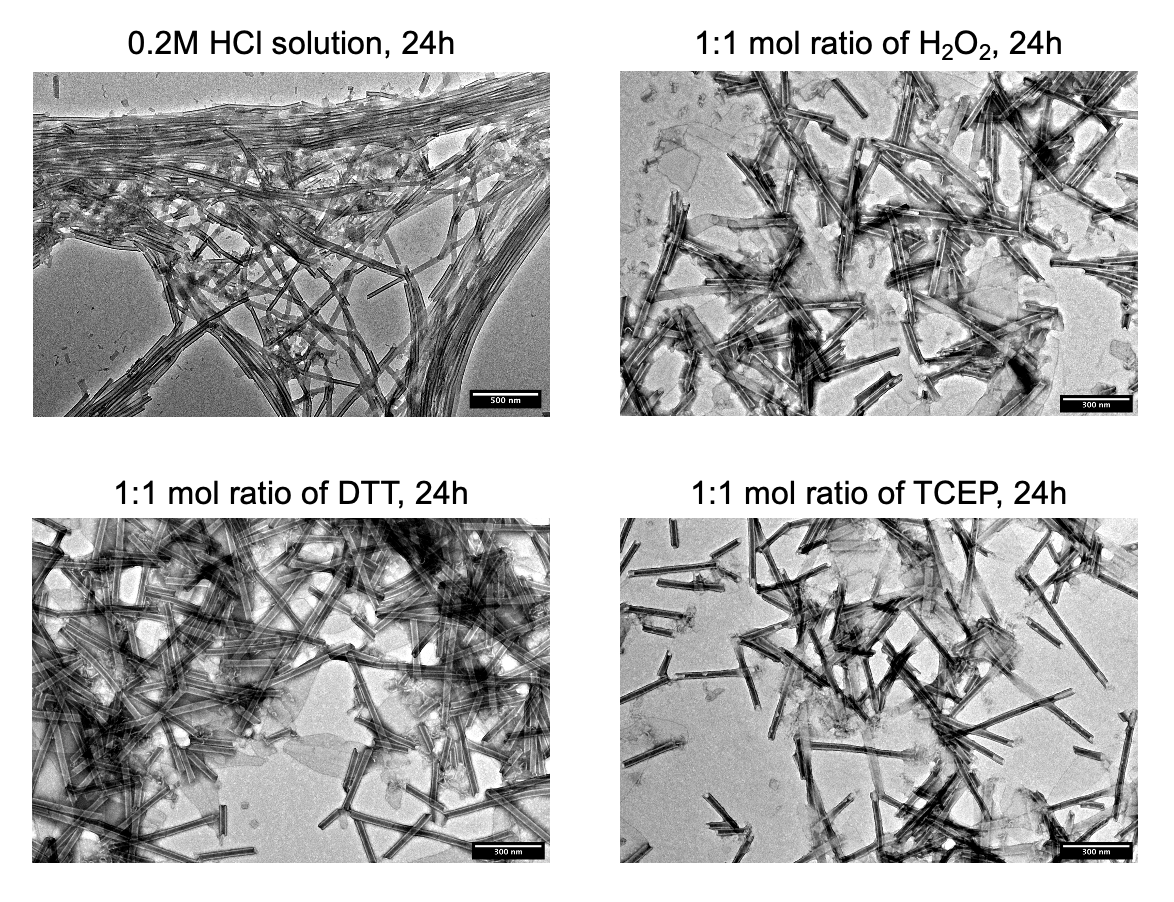


**Supplementary Figure 44 |** Representative conventional TEM images of CysAA nanotubes after treatment for 24 hours with 0.2M HCl, or with a 1:1 molar ratio of H_2_O_2_, DTT and TCEP.

**Supplementary Note 13:** AuNP aggregation on non-thiolated AA nanostructures


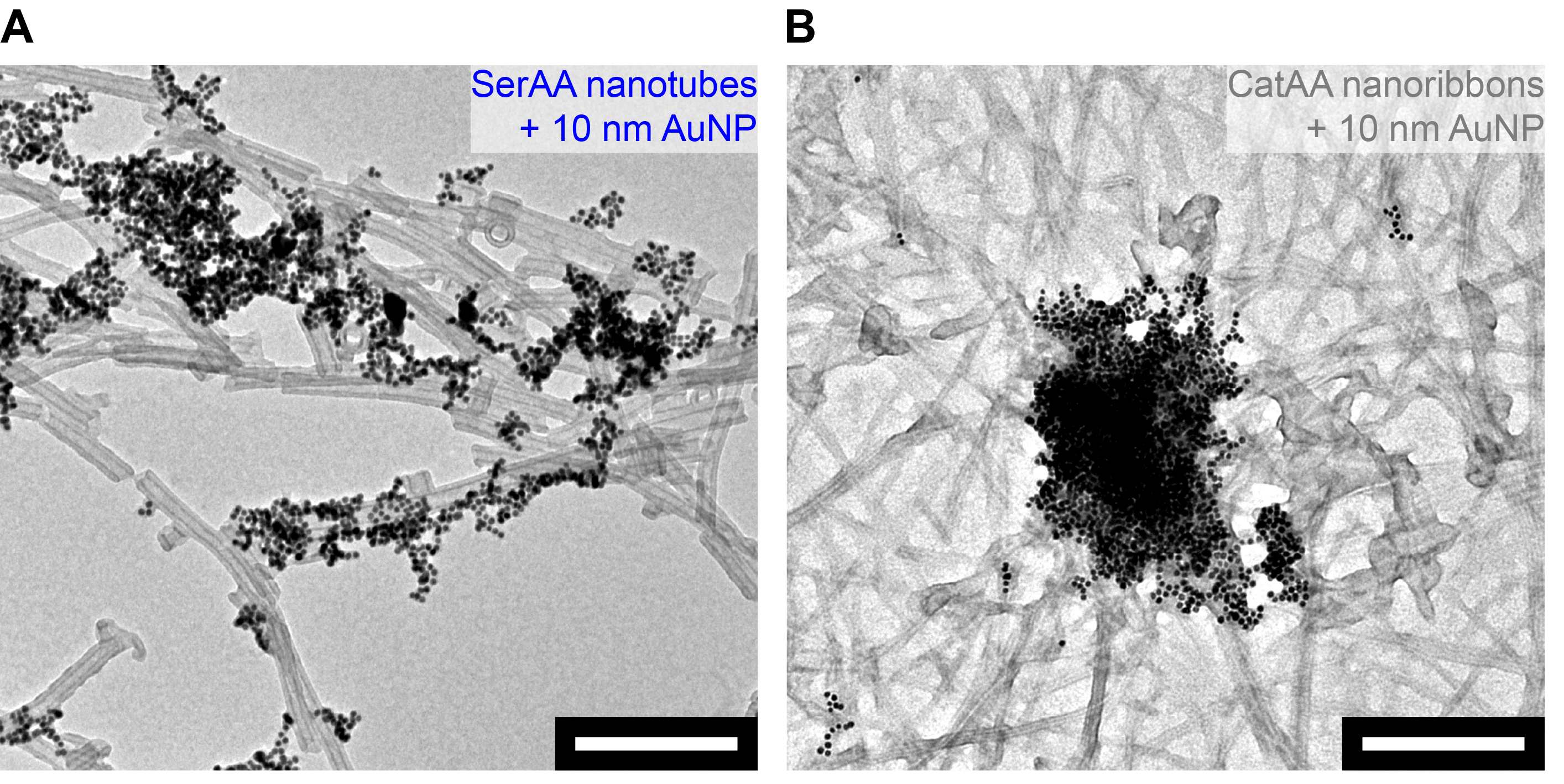


**Supplementary Figure 45 |** Representative conventional TEM images of (a) SerAA nanotubes and (e) CatAA nanoribbons, each mixed with 10 nm diameter AuNPs. Scale bars in both images represent 200 nm.

**Supplementary Note 14:** XPS correction

**Supplementary Figure 46 |** XPS spectra of the carbon C 1s peak for AuNP-functionalized AA nanostructures, with AuNP alone serving as a control. The binding energies in these spectra have been corrected using the C-C component at 284.8 eV. Peaks corresponding to O-C-O and O-C=O components are observed at 286 and 288.5 eV, respectively, across all samples.

**Supplementary Note 15:** Tunable AuNP loading on CysAA nanotube

Although it is difficult to confirm whether AuNPs are anchored to the inner surfaces of CysAA nanotubes, we suspect that some AuNPs overcome the sterically more difficult‑to‑access inner sites by diffusing through openings at the tube edges. This hypothesis is primarily based on our observation that more AuNPs appear attached along the inner side of the nanotube wall in top‑down views, rather than in the central, wall‑free regions, as highlighted by the red overlay areas in Supplementary Figure 41. Regardless, we expect that AuNPs anchored on the nanotube outer surfaces play a more critical role in catalysis than those on the inner surfaces because reactant diffusion into and product diffusion out of the nanotube interior is kinetically limited. In this respect, the inner surfaces of the nanotubes behave similarly to the inaccessible surfaces of high‑surface‑area porous catalyst supports, where liquid‑phase reactions are hindered by restricted pore accessibility and render those regions less contributive to the overall catalytic performance of the system.^[15]^

**
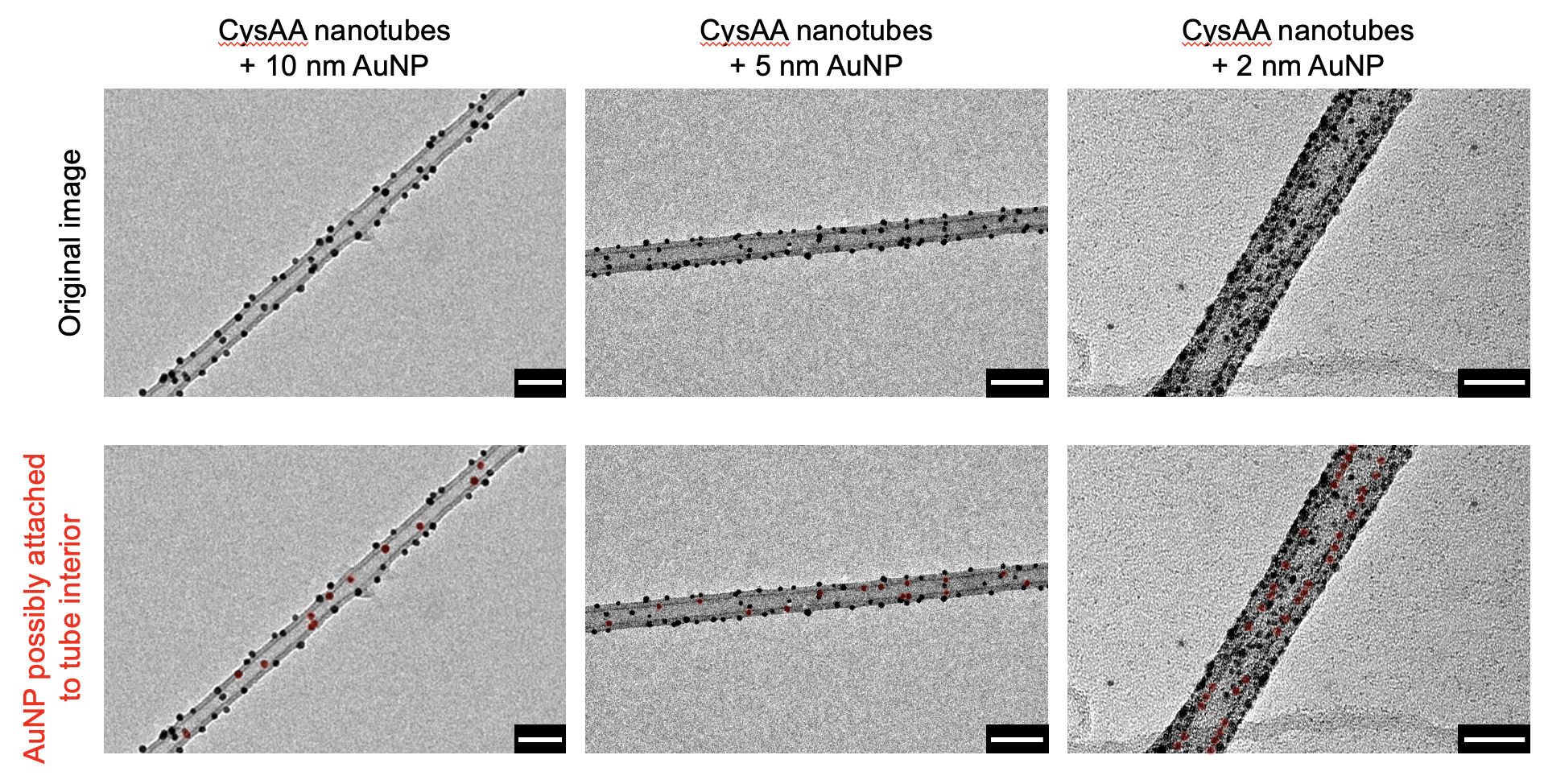
**

**Supplementary Figure 47 |** Representative conventional TEM images of CysAA nanotubes with 10, 5, and 2 nm AuNPs. AuNPs highlighted with a red overlay are those possibly attached to tube interior. Scale bars: 50 nm for 10 and 5 nm AuNP images, and 20 nm for the 2 nm AuNP images.


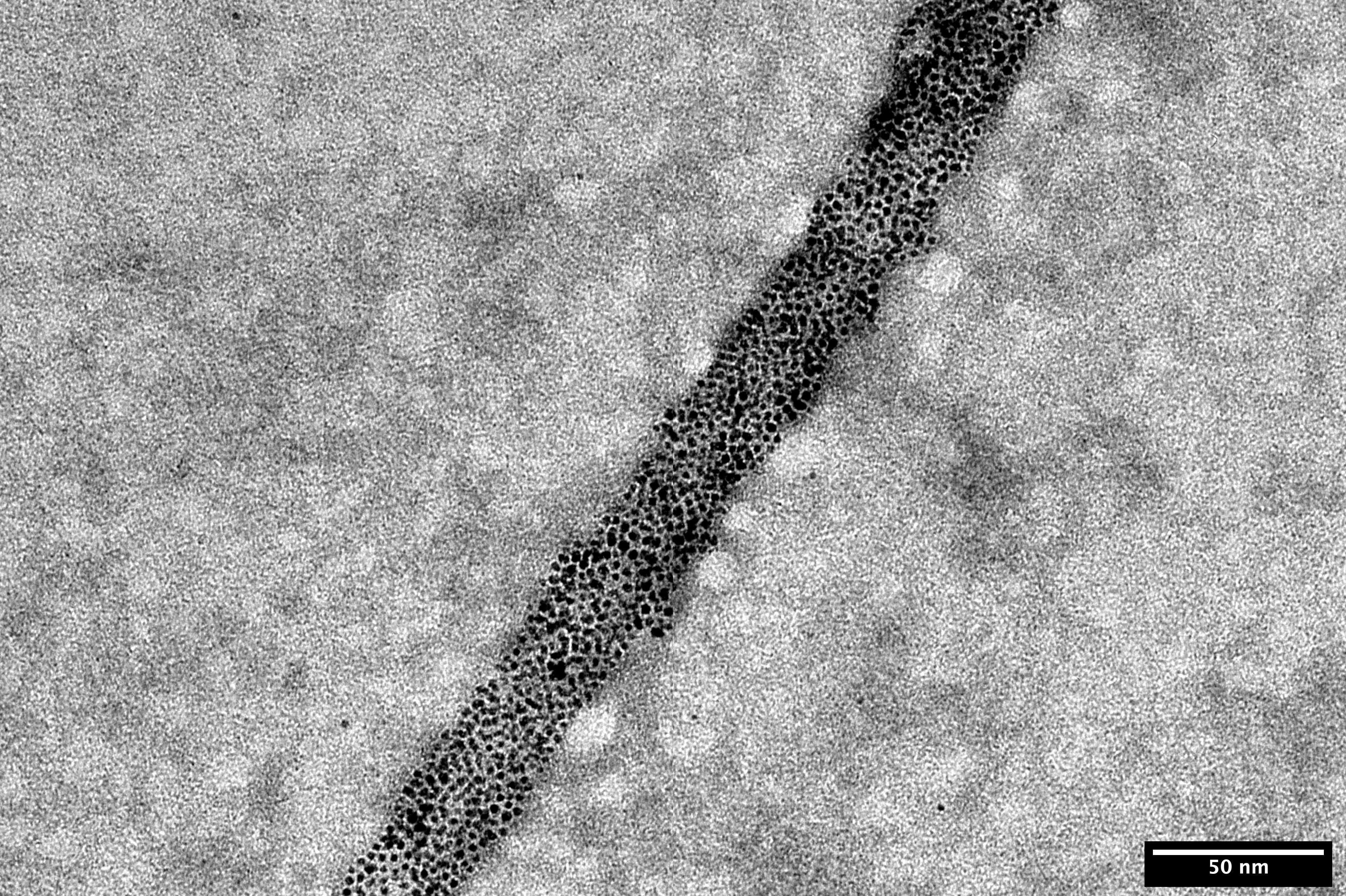


**Supplementary Figure 48 |** Representative conventional TEM images of fully loaded CysAA nanotube-supported 2 nm AuNPs. Compared to the loading conditions in the main manuscript, where the volumetric ratio of CysAA nanotubes to the AuNP solution was fixed at 1:10, this fully loaded state was achieved using a 1:1000 ratio. This condition was intentionally designed to saturate the nanotube surface with AuNPs relative to the molar availability of CysAA thiol functional groups.

Weight loading ratios and binding isotherms were determined by analyzing the residue gold concentration after AA nanotube-supported AuNPs were removed by filtration. The isotherm data were fitted using the 4-parameter Hill equation:

$$\Phi_{Bonded AuNPs}=A_{\text{base}}+ \frac{(A_{\text{max}}-A_{\text{base}})\times{C_{\text{AA}}}^{n}}{{K_{d}}^{n}+{C_{\text{AA}}}^{n}}$$

Where $\Phi_{Bonded AuNPs}$ is the fraction of AuNPs bonded on nanotubes, $A_{\text{base}}$ and $A_{\text{max}}$ are the minimum and maximum asymptote, $C_{\text{AA}}$ is the concentration of AA (in μM), $n$ is the Hill coefficient, and $K_{d}$ is the apparent dissociation constant (in μM).

**Supplementary Table 5 |** Fitted parameters for binding isotherms of CysAA nanotube suspensions (1 mg/mL) with AuNP solutions at their as-received concentrations.


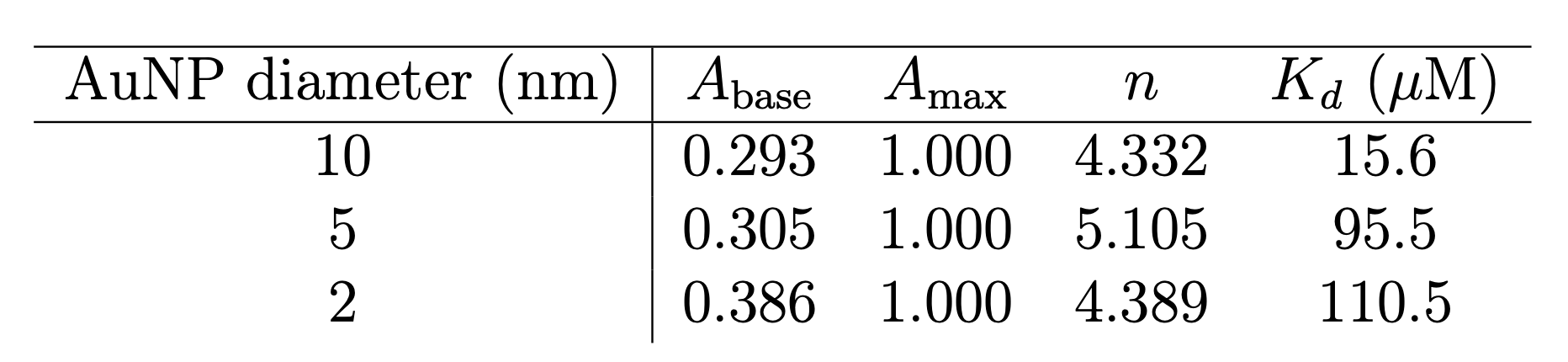


**Supplementary Note 16:** Retention of AuNP-functionalized AA nanotubes

**
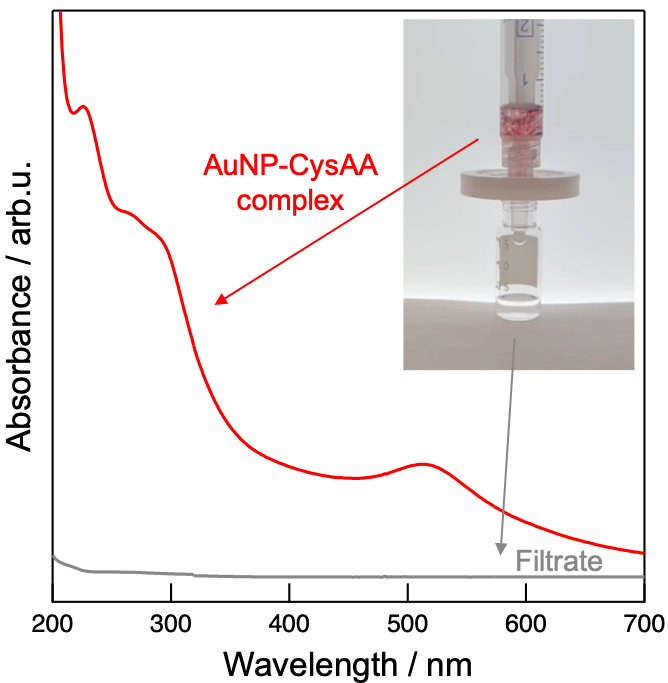
**

**Supplementary Figure 49 |** The UV-vis spectrum of the filtrate shows an absence of absorbance signals from CysAA nanotubes (260 ~ 300 nm) and AuNPs (~520 nm) when compared to the AuNP-CysAA complex. This result indicates that the majority of the AuNP-functionalized AA nanotubes are retained on the filter during filtration through a membrane with 0.2 μm pore sizes.

**Supplementary Figure 50 |** The elemental concentration of gold, as measured by ICP-MS, in each separation cycle's filtrate, is depicted. Each cycle represents the process of separating AuNP-functionalized AA nanotubes through filtration, followed by resuspending the nanotubes in DI water by backwashing the filter. Notably, gold, primarily from AuNPs, becomes nearly undetectable after the first cycle.

**
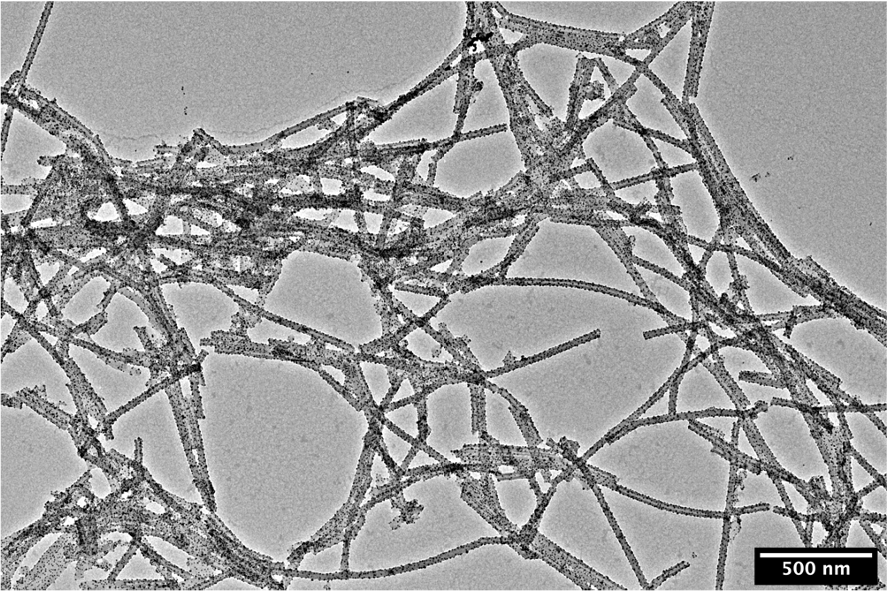
**

**Supplementary Figure 51 |** Representative conventional TEM images of AuNP-CysAA nanotubes, recovered after 10 cycles of catalyzing the conversion of 4-nitrophenol to 4-aminophenol with NaBH_4_.

**Supplementary Figure 52 |** The elemental concentration of gold, as measured by ICP-MS in each reaction cycle's filtrate, is depicted. Each cycle involves completing the reaction of 4-nitrophenol to 4-aminophenol, separating AuNP-functionalized AA nanotubes from reactant and products through filtration, and followed by resuspending the nanotubes in DI water by backwashing the filter.

**
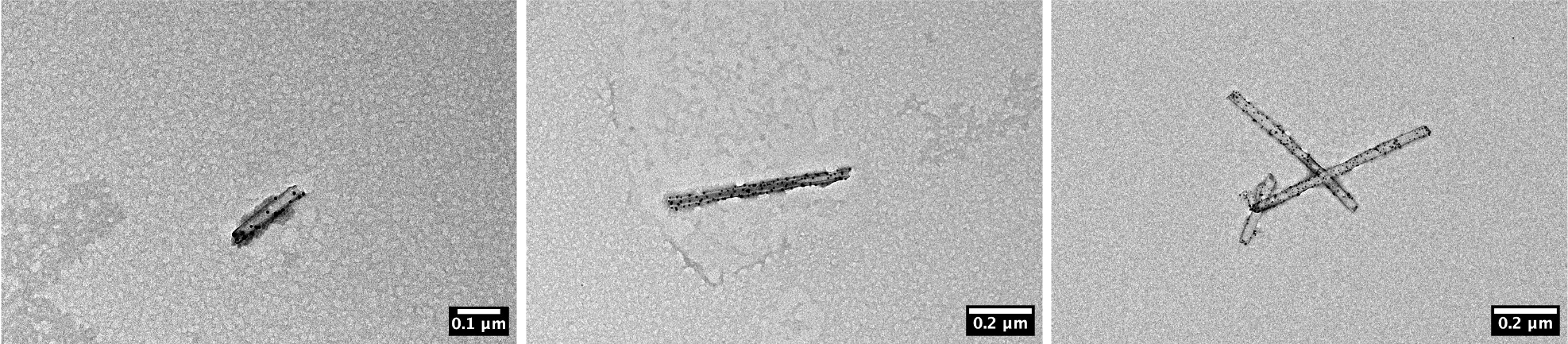
**

**Supplementary Figure 53 |** Conventional TEM images of the filtrate after 10 reaction cycles, showing occasional fragmented AuNP-functionalized AA nanotubes. The images reveal short, fragmented AuNP-functionalized AA nanotubes that have passed through the filter membrane and accumulated in the filtrate. This fragmentation may contribute to the observed decline in catalytic performance when recycling and reusing the AuNP-functionalized AA nanotubes as nanocatalysts.

**
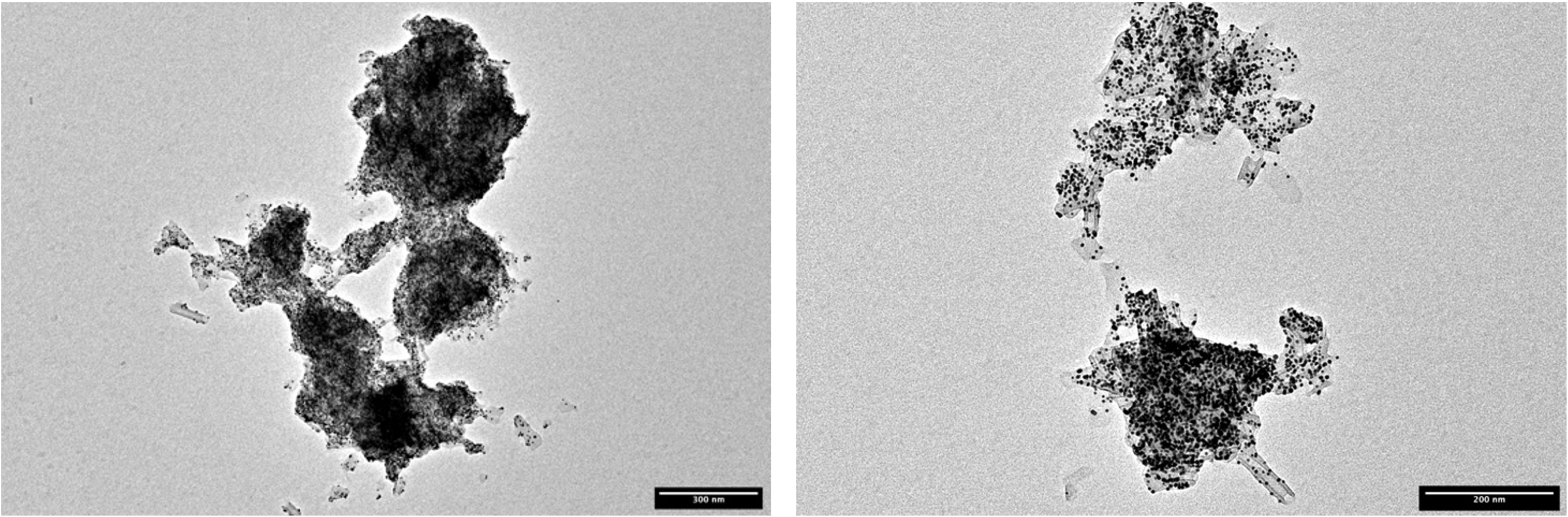
**

**Supplementary Figure 54 |** Conventional TEM images of AuNP-supported AA nanotubes following separation from solution by centrifugation. The high density of surface thiol groups on the nanotubes promotes significant agglomeration under strong centrifugal forces, likely due to close physical contact between nanotubes that facilitates inter-tube crosslinking or aggregation via residual thiol–Au interactions. Centrifugation conditions: 10,000 rpm (9,335 × g) for 3 minutes.

**Supplementary References**

1. Wang L, *et. al.*, The reformation of catalyst: From a trial-and-error synthesis to rational design, *Nano Research*, **17**, 3261-3301 (2024).

2. Harris LK and Theriot JA, Surface Area to Volume Ratio: A Natural Variable for Bacterial Morphogenesis, *Trends in Microbiology*, **26**, 815-832 (2018).

3. Sherck N, *et. al.*, End-to-End Distance Probability Distributions of Dilute Poly(ethylene oxide) in Aqueous Solution, *Journal of the American Chemical Society*, **142**, 19631-19641 (2020).

4. Doi M, Edwards S F, The Theory of Polymer Dynamics, Clarendon Press (1988).

5. Bouchiat C, *et. al.*, Estimating the Persistence Length of a Worm-Like Chain Molecule from Force-Extension Measurements, *Biophysical Journal*, **76**, 409-413 (1999).

6. Chen S, *et. al.*, Surface chemistry and catalysis of oxide model catalysts from single crystals to nanocrystals, *Surface Science Reports*, **74**, 100471 (2019).

7. Christoff-Tempesta T, *et. al.*, Interfacial dynamics mediate surface binding events on supramolecular nanostructures, *Nature Communications*, **15**, 7749 (2024).

8. Kirby, B. J., Micro- and Nanoscale Fluid Mechanics: Transport in Microfluidic Devices, Cambridge University Press (2010).

9. Christoff-Tempesta T, *et. al.*, Self-assembly of aramid amphiphiles into ultra-stable nanoribbons and aligned nanofiber threads, *Nature Nanotechnology*, **16**, 447-454 (2021).

10. Cho Y, *et. al.*, Domain-selective thermal decomposition within supramolecular nanoribbons, *Nature Communication*, **12**, 7340 (2021).

11. Kim D-Y and Christoff-Tempesta T, *et. al.*, Morphological transitions of a photoswitchable aramid amphiphile nanostructure, *Nano Letter*, **21**, 2912-2918 (2021).

12. Kasar SJ and Lew AJ, *et. al.*, Effects of molecular flexibility and head group repulsion on aramid amphiphile self-assembly, *Molecular System Design & Engineering*, **6**, 1016-1024 (2021).

13. Lamour G, *et. al.*, High Intrinsic Mechanical Flexibility of Mouse Prion Nanofibrils Revealed by Measurements of Axial and Radial Young’s Moduli, *ACS Nano*, **8**, 3851-3861 (2014).

14. Lamour G, *et. al.*, Easyworm: an open-source software tool to determine the mechanical properties of worm-like chains., *Source code for biology and medicine*, **9**, 1-6 (2014).

15. Broccoli A, *et. al.*, Accessibility study of porous materials at the single-particle level as evaluated within a microfluidic chip with fluorescence microscopy., *Chem Catalysis*, **3**, 11, 100791 (2023).
